# Hydrogen sulfide alleviates thiocyanate stress in rice seedlings via tissue-specific carbon and nitrogen metabolic reprogramming

**DOI:** 10.3389/fpls.2026.1769534

**Published:** 2026-04-27

**Authors:** Meng-Hua Chen, Hui-Lin Chen, Yu-Xi Feng, Yu-Juan Lin, Yan-Hong Li

**Affiliations:** 1 Guangxi Key Laboratory of Environmental Pollution Control Theory and Technology, Guilin University of Technology, Guilin, China; 2 College of Environmental Science and Engineering, Guilin University of Technology, Guilin, China; 3 Engineering Research Center of Watershed Protection and Green Development, University of Guangxi, Guilin University of Technology, Guilin, China; 4 Guangdong‑Hong Kong Joint Laboratory for Carbon Neutrality, Jiangmen, China; 5 Jiangmen Laboratory of Carbon Science and Technology, Jiangmen, China

**Keywords:** carbon and nitrogen metabolism, H2S, metabolic network, rice, thiocyanate

## Abstract

Thiocyanate (SCN^−^), an industrially pervasive chemical, has pronounced phytotoxicity, resulting in compromised root development, photosynthetic inhibition, and stunted growth in crops. Hydrogen sulfide (H_2_S) is recognized as a gaseous signaling molecule crucial for plant stress responses; however, the precise mechanisms by which H_2_S enhances rice tolerance to SCN^−^ stress through metabolic network reconfiguration remain elusive. By utilizing an integrated approach combining biochemical analysis, widely-targeted metabolomics, and weighted gene coexpression network analysis (WGCNA), this study systematically deciphered the regulatory role of exogenous H_2_S in reshaping the metabolic network of rice seedlings under SCN^−^ stress. Our findings reveal that H_2_S alleviates SCN^−^ toxicity via tissue-specific reprogramming of carbon (C) and nitrogen (N) metabolism. In roots, H_2_S stabilizes carbon accumulation (CA) and nitrogen accumulation (NA), with purine metabolism and riboflavin metabolism serving as core metabolic hubs that cooperatively ensure energy supply and membrane integrity, thereby establishing the foundation for systemic stress resistance in the root system. While in shoots, H_2_S maintains high levels of CA and NA, triggering extensive metabolic remodelling by driving the synthesis of flavonoids, phenolic acids and terpenoids. This increased antioxidant and detoxification capacities confirmed that shoots are the key site for H_2_S-mediated stress alleviation. H_2_S enhances rice seedling tolerance to SCN^−^ stress through systematic reconfiguration of C and N metabolism, providing a vital theoretical foundation for the ecological restoration of contaminated farmlands and the development of stress-resistant crop varieties.

## Graphical Abstract

**Figure:**
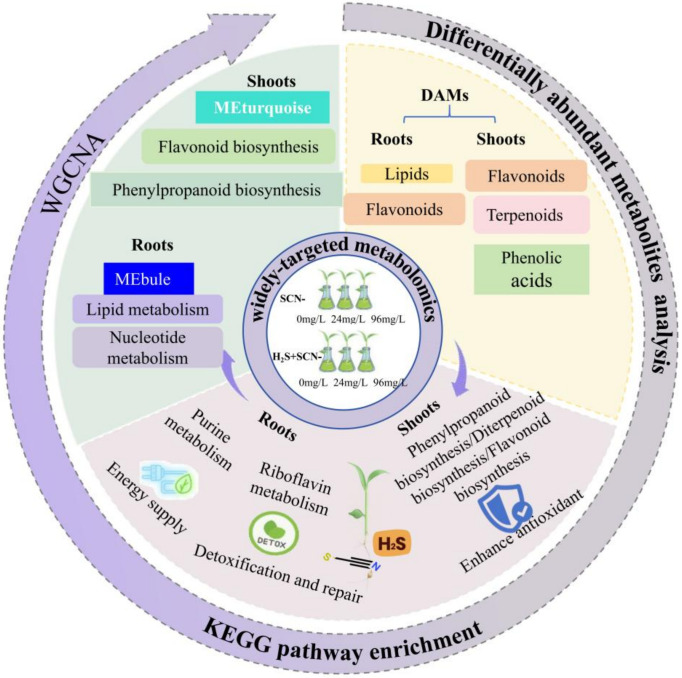

## 1 Introduction

Metabolites, which serve as the terminal products of cellular metabolism, offer a precise reflection of the cellular physiological state. Plants meticulously modulate the composition and concentration of metabolites to acclimate to environmental perturbations (Sun et al., 2023). Estimates suggest that the plant metabolome encompasses between 200,000 and 1,000,000 distinct metabolites (Maeda and Fernie, 2021). Under adverse conditions, hundreds of metabolites undergo rapid synthesis or transformation, which directly encapsulates the adaptive capacity of plants. Metabolites are integral to a myriad of key physiological processes in plants, including growth and development, maintenance of cellular integrity, energy storage, signal transduction, resource allocation, developmental regulation, and stress response (Salam et al., 2023). Research has demonstrated that under pollution stress, metabolites in plant leaves bolster the plant’s defense mechanisms by preserving the osmotic equilibrium and energy supply of the plant (Chakraborty et al., 2023; Salam et al., 2023). Moreover, plants augment the synthesis of secondary metabolites (such as phenols and flavonoids), which exhibit significant antioxidant and detoxification properties (Ni et al., 2020; Zhuang et al., 2024; Li et al., 2021). Thus, an in-depth dissection of the global reconfiguration of metabolic networks is pivotal for elucidating plant stress resistance mechanisms.

Thiocyanate (SCN^−^), an industrially ubiquitous chemical, has been extensively applied across diverse industrial domains, including insecticides, herbicides, metallurgy, electroplating, dyeing, and thiourea (Shafiei et al., 2021). Regrettably, the absence of SCN^−^ from current regulatory control indices has led to the widespread neglect of soil contamination by SCN^−^ in the vicinity of factories and mines, thereby leaving the potential threat to crop production underrecognized (Kohio et al., 2025). Empirical studies have underscored the heightened sensitivity of rice seedlings to SCN^−^ stress, with SCN^−^ exposure precipitating marked inhibition of root morphological characteristics and disruption of root development (Feng et al., 2021; Lin et al., 2021). SCN^−^ stress downregulates genes associated with photosynthesis (such as the D1 protein turnover gene) and energy metabolism pathways, culminating in diminished levels of soluble starch, sugars, and chlorophyll, which in turn stymie the overall growth of rice seedlings (Yang et al., 2021). Transcriptomic analyzes revealed that SCN^−^ exerts various toxic effects on rice seedlings by disrupting the equilibrium of endogenous plant hormones and key metabolic pathways, with the most pronounced disruptions occurring in metabolic pathways and secondary metabolite synthesis pathways (Tian et al., 2023).

Hydrogen sulfide (H_2_S), a novel gaseous signaling molecule, activates the antioxidant system and maintains cellular redox homeostasis, thereby playing a pivotal protective role in plant responses to abiotic stresses such as heavy metals, salinity, and drought (Aroca et al., 2021; Corpas, 2019; de Bont et al., 2022; Singh et al., 2022; Zhang et al., 2024). Research has shown that H_2_S enhances the repair capacity of photosystem II (PSII), upregulates the expression of genes related to secondary metabolite synthesis, metabolic pathways, and signal transduction in stem tissues, and it mediates the sulfhydration of proteins, thereby orchestrating a multidimensional regulatory response to alleviate SCN^−^ toxicity (Gotor et al., 2019; Feng et al., 2021; Tian et al., 2023; Yang et al., 2021). Herein, key questions remain regarding how H_2_S systematically reorganizes the metabolic network of rice to synergistically enhance stress resistance and whether there are differential regulatory mechanisms across different tissues.

Recently, metabolomics provides a powerful approach for elucidating the regulatory mechanisms of metabolic networks and can comprehensively dissect the response mechanisms of plants to environmental changes by studying the collection of all metabolites within cells at a specific time point (Gevi et al., 2019). In this study, to investigate the regulatory effects of H_2_S on the metabolic network of rice seedlings under SCN^−^ stress, the following steps were performed: 1) we estimated the relative growth rate (RGR) and C:N ratio (C/N) of rice seedlings under SCN^−^ stress with or without exogenous H_2_S; 2) we used widely-targeted metabolomics to analyze changes in metabolites across different tissues; and 3) Kyoto Encyclopedia of Genes and Genomes (KEGG) pathway enrichment analysis and weighted gene coexpression network analysis (WGCNA) were used to identify metabolic pathways and metabolites associated with H_2_S treatment. Overall, this study provides a scientific basis for the future development of targeted chemical regulators that act on key metabolic pathways for the ecological restoration of SCN^−^-contaminated farmlands.

## 2 Materials and methods

### 2.1 Experimental materials

Rice seeds (*Oryza sativa* L. cv. XZX 45), a regular medium-maturing indica rice variety, were used in this study (Feng et al., 2023). After surface sterilization with 3% H_2_O_2_ and several rinses in deionized water, the seeds were sown in plastic containers filled with sandy loam. Seedlings were grown in a controlled-environment chamber set at 25 ± 0.5 °C and 60 ± 2% relative humidity and irrigated thoroughly with a modified ISO 8692 nutrient solution (Zhang et al., 2021). Sixteen days after germination, uniformly sized seedlings were selected and transferred to the same nutrient mixture supplemented with potassium thiocyanate.

### 2.2 Experimental design and treatments

After a 12-h equilibration in nutrient solution, the rice seedlings were subjected to the following treatments:

(1) SCN^−^ treatment: Plants were exposed for 3 d to solutions containing 0 (C0), 24.0 (C1) or 96.0 (C2) mg SCN^−^ L^-1^ (Tian et al., 2023).

(2) H_2_S + SCN^−^ treatment: Plants were pretreated for 6 h with 100 µM NaHS (as an H_2_S donor) and then transferred for 3 d to fresh solutions containing 0 (T0), 24.0 (T1) or 96.0 (T2) mg SCN^−^ L^-1^. The concentration of 100 µM NaHS was selected based on previous studies indicating this concentration effectively induces H_2_S signaling without phytotoxicity in rice seedlings (Tian et al., 2023; Feng et al., 2021).

At the end of the stress period, the roots were rinsed, and the plants were harvested for downstream analyzes. Each treatment included four independent biological replicates (four trays, 10 seedlings per tray). For metabolomics, three randomly selected seedlings per replicate were pooled, homogenized, and analyzed in duplicate; only features with a coefficient of variation (CV) < 15% between technical replicates were retained for statistical analysis (Gevi et al., 2019).

The two SCN^−^ concentrations corresponded to the EC_20_ (24.0 mg SCN^−^ L^-1^, 20% growth inhibition) and EC_50_ (96.0 mg SCN^−^ L^-1^ 50% growth inhibition) values. The culture vessels were wrapped in aluminum foil throughout the experiment to minimize evaporation and suppress algal growth (Lin et al., 2020).

To dissect the alleviating mechanism of H_2_S on SCN^−^ stress in rice seedlings, the following paired comparative analysis was conducted:

1. Stress effect analysis: comparisons of C1/C0 and C2/C0 were established to characterize the metabolic responses induced by SCN^−^ stress alone (EC_20_ and EC_50_), thereby establishing the stress baseline;
2. H_2_S-specific effect analysis: comparisons of T0/C0 was established to dissect the metabolic effects induced by H_2_S treatment alone;
3. Alleviating effect analysis: comparisons of T1/C1 and T2/C2 were configured to elucidate the specific regulatory effects of H_2_S treatment under identical SCN^−^ stress concentrations, thereby identifying the core metabolic targets mediating H_2_S-induced stress alleviation.

### 2.3 Physiological state analysis

#### 2.3.1 Measurement of growth parameters

The RGR was analyzed to quantify the toxic effect of SCN^−^ on rice seedlings via the following Equation 1 (Yu and Zhang, 2013):

(1) $$RGR=\frac{W_{\left(F\right)}-W_{\left(I\right)}}{W_{\left(F\right)}}\times 100\%$$

where *W*_(_*_F_*_)_ and *W*_(_*_I_*_)_ represent the final and initial fresh weights of the rice seedlings, respectively.

#### 2.3.2 Measurement of C and N accumulation

After 3 days of SCN^−^ or H_2_S + SCN^−^ treatment, the rice seedlings were harvested and separated into roots and shoots. After being washed with double-distilled water, the plant materials were oven dried at 90 °C for 48 h and weighed. Then, 0.010 g of oven-dried plant material was ground into a fine powder. The total C and N (%) were measured via a vario elemental analyzer (vario EL) (Brown et al., 2007). The C/N ratio, C accumulation and N accumulation (CA and NA, mg/g *DW*) were quantified via the following Equations 2–4:

(2) $$\frac{C}{N}=\frac{C\%}{N\%}$$

(3) $$CA=\frac{DW\times C\%}{100}$$

(4) $$NA=\frac{DW\times N\%}{100}$$

where *C%* and *N%* represent the total C and N contents (%), respectively. *DW* represents the dry weight of the rice seedlings.

### 2.4 Widely-targeted metabolomic profiling

This study employed a widely targeted metabolomics approach based on UPLC-QTRAP-MS/MS with multiple reaction monitoring (MRM), combining the high-throughput coverage of untargeted metabolomics with the high sensitivity and accurate quantification advantages of targeted metabolomics.

#### 2.4.1 Sample collection and metabolite extraction

After 3 days of SCN^−^ or H_2_S + SCN^−^ treatment, the rice seedlings were harvested. The roots were gently rinsed, and each plant was separated into root and shoot fractions. The biological samples were placed in a lyophilizer (Scientz100F), vacuum freeze-dried, and then ground (30 Hz, 1.5 min) to powder form by using a grinder (MM 400, Retsch). Next, 50 mg of sample powder was weighed on an electronic balance (MS105DM) and transferred to a tube; 1200 μL of 70% methanolic aqueous internal standard extract precooled to -20 °C was added (less than 50 mg added at a rate of 1200 μL extractant per 50 mg sample). The mixture was vortexed once every 30 min for 30 sec, for a total of 6 times (Gevi et al., 2019). After centrifugation (with a rotation speed of 12000 rpm for 3 min), the supernatant was aspirated, and the sample was filtered through a microporous membrane (0.22 μm pore size) and stored in an injection vial for UPLC–tandem mass spectrometry (UPLC–MS/MS) analysis.

#### 2.4.2 UPLC conditions

The sample extracts were analyzed via a UPLC–ESI–MS–MS–MS system (UPLC, ExionLC™ AD, https://sciex.com.cn/) and a tandem mass spectrometry system (https://sciex.com.cn/). The analytical conditions were as follows: UPLC: column, Agilent SB-C18 (1.8 µm, 2.1 mm×100 mm). The mobile phase consisted of solvent A, pure water with 0.1% formic acid, and solvent B, acetonitrile with 0.1% formic acid. Sample measurements were performed with a gradient program that employed the starting conditions of 95% A, 5% B. Within 9 min, a linear gradient to 5% A, 95% B was programmed, and a composition of 5% A, 95% B was maintained for 1 min. Subsequently, a composition of 95% A, 5.0% B was adjusted within 1.1 min and maintained for 2.9 min. The flow velocity was set as 0.35 mL per minute. The column oven was set to 40 °C, and the injection volume was 2 μL (Mori et al., 2023). The effluent was alternatively connected to an ESI-triple quadrupole-linear ion trap (QTRAP)-MS.

#### 2.4.3 ESI-Q TRAP-MS/MS conditions and data acquisition

The ESI source operation parameters were as follows (Lowe et al., 2021): source temperature, 500 °C; ion spray voltage (IS), 5500 V (positive ion mode)/-4500 V (negative ion mode); ion source gas I (GSI), gas II (GSII), and curtain gas (CUR) were set at 50, 60, and 25 psi, respectively; and collision-activated dissociation (CAD) was high. QQQ scans were acquired as MRM experiments with the collision gas (nitrogen) set to medium. Declustering potential (DP) and collision energy (CE) for individual MRM transitions were performed with further DP and CE optimization. A specific set of MRM transitions was monitored for each period according to the metabolites eluted within this period. When the MRM signal exceeded the set threshold, the system automatically triggered enhanced product ion (EPI) scanning to acquire tandem mass spectrometry (MS^2^) spectra for subsequent qualitative analysis of the corresponding metabolites.

#### 2.4.4 Metabolite identification and quantification

Metabolite identification was performed based on MS² spectral information using an in-house database (>10,000 rice-specific ion pairs). Isotope signals, duplicate signals containing K^+^, Na^+^, and NH_4_^+^ adducts, as well as fragment ions originating from larger molecular weight compounds, were excluded during analysis. A spectral match score≥80 and an MS¹mass tolerance ≤ 5 ppm were required for confident identification (Shahaf et al., 2016).

For quantification, MRM data were processed using MultiQuant software. Chromatographic peaks of each metabolite were integrated to obtain peak areas as relative quantification values. Peak alignment was performed across all samples for each metabolite to ensure quantitative accuracy (Fraga et al., 2010).

To ensure data reproducibility and reliability, a comprehensive quality control (QC) analysis was performed. Total ion chromatogram (TIC) overlay analysis of pooled QC samples revealed highly consistent retention times and peak intensities, indicating excellent signal stability of the mass spectrometry detection (**Supplementary Figure S1**). Additionally, quantitative analysis with integration correction was conducted on randomly selected metabolites to validate the accuracy of peak alignment (**Supplementary Figures S2**, **S3**). The CV distribution across all samples showed that over 85% of features in QC samples exhibited CV values below 0.5, demonstrating high data stability throughout the entire analytical workflow and providing a robust foundation for subsequent differential analysis (**Supplementary Figure S4**).

### 2.5 Data analysis and bioinformatics

#### 2.5.1 Principal component analysis

Unsupervised principal component analysis (PCA) was performed via the statistics function prcomp within R (www.r-project.org). The data were subjected to unit variance scaling (UV) before unsupervised PCA. UV, also known as Z score standardization or autoscaling, is a method that standardizes data on the basis of the mean and standard deviation of the original dataset. The resulting data followed a standard normal distribution, with a mean of 0 and a standard deviation of 1 (Xu et al., 2023). Specifically, each value was transformed by subtracting the mean and then dividing by the standard deviation. The calculation formula is as follows:

(5) $$\chi^{\prime }=\frac{\chi -\mu }{\sigma }$$

where *μ* is the mean and *σ* is the standard deviation.

#### 2.5.2 Hierarchical cluster analysis

Metabolite content data were subjected to UV (Equation 5), and a heatmap was generated with the R package ComplexHeatmap to perform hierarchical cluster analysis (HCA) of metabolite accumulation patterns across samples.

#### 2.5.3 Differentially abundant metabolites selected

Differentially abundant metabolites (DAMs) were determined by variable importance in projection (VIP > 1), absolute log2 fold change (|Log2FC| ≥ 1.0) and *P* value (*P*≤ 0.05) (Ghorbanzadeh et al., 2023). VIP values were extracted from the orthogonal partial least squares discriminant analysis (OPLS-DA) results, which also contain score plots and permutation plots generated via the R package MetaboAnalystR. The data were log transformed (log2) and mean centered before OPLS-DA. To avoid overfitting, a permutation test (200 permutations) was performed (Xu et al., 2023).

#### 2.5.4 KEGG annotation and enrichment analysis

The identified metabolites were annotated via the KEGG database (http://www.kegg.jp/kegg/compound/), and the annotated metabolites were then mapped to the KEGG database (http://www.kegg.jp/kegg/pathway.html) (Ogata et al., 1999). On the basis of the DAMs, KEGG pathway enrichment analysis was performed. The rich factor is the ratio of the number of DAMs in a given pathway to the total number of metabolites annotated to that pathway; the higher the value is, the greater the enrichment. Pathways were determined by the *P* value (*P ≤* 0.05) (Ghorbanzadeh et al., 2023). The *P* value was the hypergeometric test *P* value, which was calculated as shown in Equation 6:

(6) $$P=1-\sum_{i=0}^{m-1}\frac{\left(\begin{array}{c} M\\ i \end{array}\right)\left(\begin{array}{c} N-M\\ n-i \end{array}\right)}{\left(\begin{array}{c} N\\ n \end{array}\right)}$$

where *N* is the total number of metabolites with KEGG annotations, *n* is the number of DAMs within *N*, *M* is the number of metabolites annotated to a specific KEGG pathway, and *m* is the number of DAMs in that pathway. A *P* value closer to 0 indicates greater enrichment significance.

#### 2.5.5 Weighted gene coexpression network analysis

A scale-free weighted coexpression network was constructed to uncover metabolite–metabolite relationships. Modules of highly coregulated metabolites were identified, offering functional insights and shedding light on the underlying regulatory mechanisms (Ahmad, 2024). WGCNA was performed via the WGCNA package in R. Modules associated with each trait were screened using a threshold of absolute correlation coefficient ≥ 0.3 and *P* value < 0.05.

#### 2.5.6 Statistical analysis

The experimental results are presented as the average value ± standard deviation. Four independent biological replicates were used for each treatment. The significance of differences between the treatment and control groups was studied via variance (two-way ANOVA) and Tukey’s multiple comparison tests, and values of *P* < 0.05 were considered statistically significant (Zar, 1999).

#### 2.5.7 Software for statistical analysis and visualization

Statistical analyzes were performed using SPSS 26.0 (IBM Corp., Armonk, NY, USA) and R v4.2.3 (www.r-project.org). PCA, HCA, OPLS−DA, WGCNA, and volcano plots were performed using R packages including MetaboAnalystR, ComplexHeatmap, and WGCNA. All figures were generated using Origin 2023 (OriginLab Corp., Northampton, MA, USA) and Adobe Illustrator 2024 or Microsoft PowerPoint (version 2021).

## 3 Results

### 3.1 H_2_S alleviates SCN^−^-induced growth inhibition and maintains C and N homeostasis in rice seedlings

**Figure 1:**
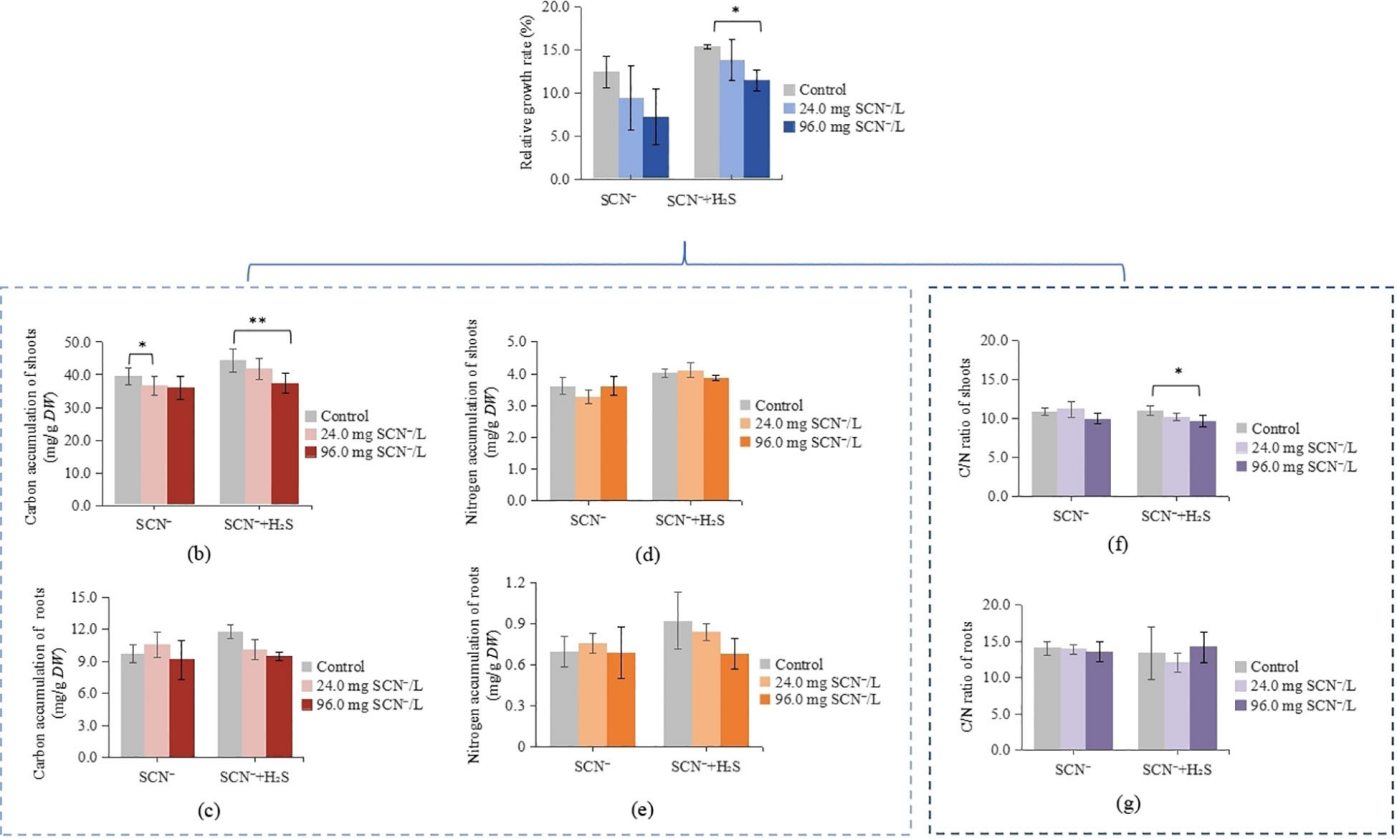
Physiological state of rice seedlings treated with SCN^-^ and H_2_S+SCN^-^. **(a)** Relative growth rate of rice seedlings. **(b)** Carbon accumulation in shoots. **(c)** Carbon accumulation in roots. **(d)** Nitrogen accumulation in shoots. **(e)** Nitrogen accumulation in roots. **(f)** C/N ratio in shoots. **(g)** C/N ratio in roots.

The RGR decreased significantly with increasing SCN^−^ concentration. Compared with that of the control (C0), the RGR decreased by 24.3% and 42.0% at the EC_20_ and EC_50_ concentrations, respectively. H_2_S+SCN^−^ treatment largely mitigated this growth inhibition, with the RGR recovering to 89.0% and 73.7% of the control level at the EC_20_ and EC_50_, respectively (**Figure 1a**). Representative phenotypic images of rice seedlings under SCN^−^ stress and H_2_S pretreatment were documented in previous studies (Feng et al., 2024), showing obvious growth recovery upon H_2_S application.

Although CA and NA in roots and shoots showed no statistically significant differences between SCN^−^ and H_2_S+SCN^−^ treatments (**Figures 1b, c, d, e**), H_2_S resulted in a recovery and relative stabilization of CA and NA compared with SCN^−^ treatments. Notably, the C/N ratio exhibited significant changes at EC_50_: H_2_S significantly decreased the C/N ratio in shoots (*P* = 0.003) while restoring that in roots compared with SCN^-^ treatment (**Figures 1f, g**). These alterations in C/N balance suggest that H_2_S may alleviate SCN^−^ stress by maintaining C and N accumulation homeostasis and restoring the C/N equilibrium.

### 3.2 H_2_S alters the metabolic profiles of rice seedlings under SCN^−^ stress

**Figure 2:**
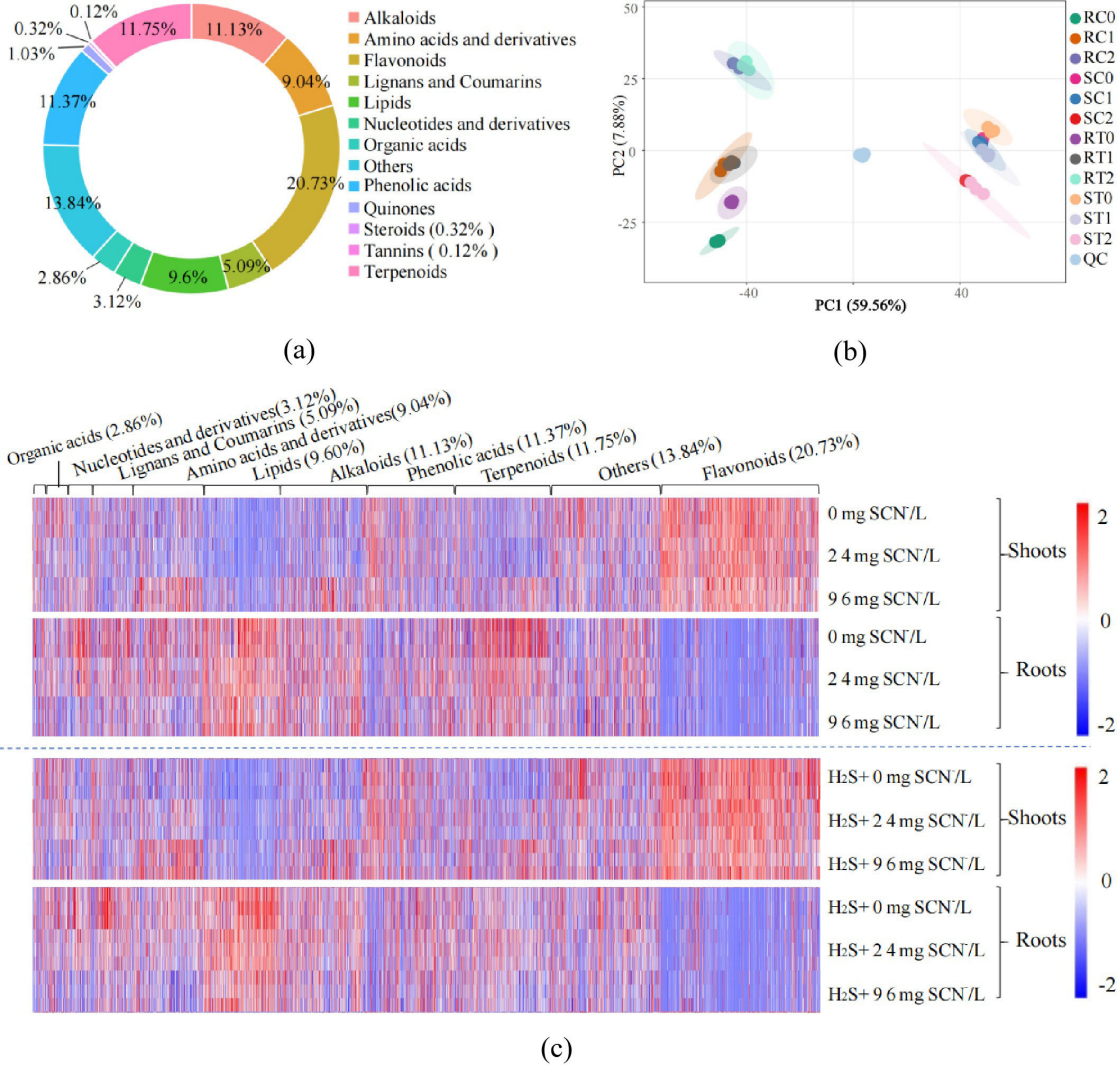
Metabolic profiles of rice seedlings treated with SCN^-^ and H_2_S+SCN^-^. **(a)** Circular chart of metabolite functions. **(b)** PCA based on all metabolites. **(c)** Heatmap of 3396 metabolites; red indicates up-regulation, blue indicates down-regulation, and white represents average expression levels. The intensity of the color reflects the magnitude of change. RC0, RC1, RC2, SC0, SC1, SC2: represent the roots and shoots of rice seedlings subjected to stress for 3 days in stress solutions with SCN⁻ concentrations of 0 (C0), 24.0 (C1), and 96.0 (C2) mg/L, respectively. RT0, RT1, RT2, ST0, ST1, ST2: represent the roots and shoots of rice seedlings that were pre-treated with exogenous H_2_S solution for 6 hours and then subjected to stress for 3 days in stress solutions with SCN^-^ concentrations of 0 (C0), 24.0 (C1), and 96.0 (C2) mg/L, respectively.

In total, 3,396 metabolites were identified, dominated by secondary metabolites—flavonoids (20.73%), phenolic acids (11.37%) and alkaloids (11.13%)—together with primary metabolites such as lipids (9.60%), amino acids and their derivatives (9.04%), underscoring the extensive metabolic activity triggered by SCN^−^ stress (**Figure 2a**). PCA clearly separated the metabolic profiles. The SCN^−^ treatment (RC0, RC1, RC2, SC0, SC1, SC2) and H_2_S+SCN^−^ treatment (RT0, RT1, RT2, ST0, ST1, ST2) were distinctly separated on PC1 axi, with PC1 explaining 59.56% of the total variance, indicating a pronounced effect of H_2_S on the metabolic landscape (**Figure 2b**). The OPLS-DA models were confirmed by high R^2^Y and Q^2^ values (≥ 0.90; **Supplementary Table S1**). Additionally, HCA highlighted tissue-specific metabolic patterns, further supporting the spatial specificity of the metabolic responses (**Figure 2c**).

### 3.3 H_2_S modifies tissue-specific changes in DAMs

#### 3.3.1 Overall changes in DAMs

**Figure 3:**
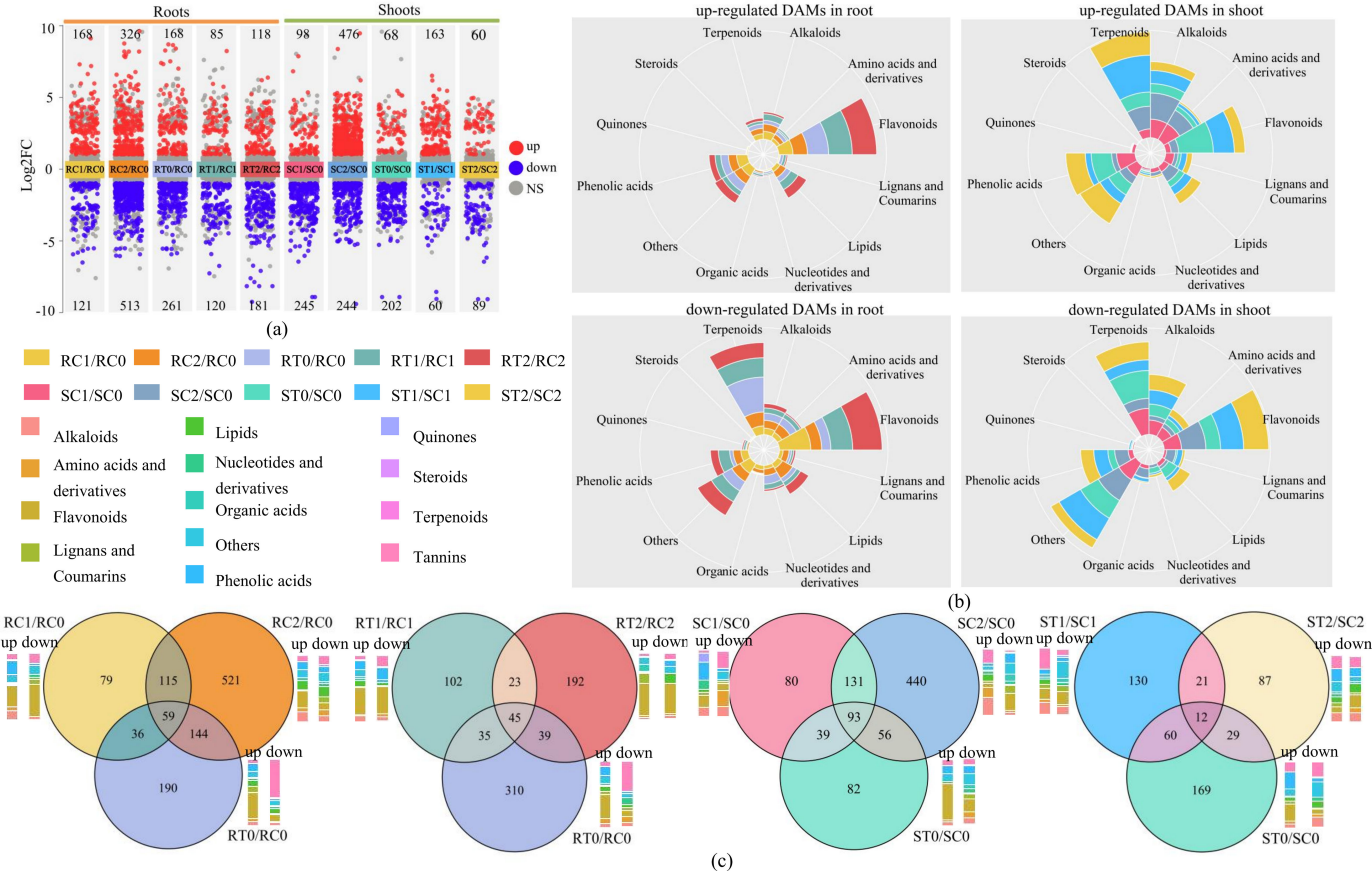
DAMs of rice seedlings treated with SCN^-^ and H_2_S+SCN^-^. **(a)** Volcano plot of DAMs. **(b)** Metabolite functions category of DAMs. **(c)** Venn diagram of DAMs. RC1/RC0: represent RC1 vs RC0, and so on for the others.

Using strict thresholds (VIP ≥ 1, P ≤ 0.05, and |log2FC| ≥ 1), we identified substantial numbers of DAMs across all comparison groups. Volcano plots visually depicted the quantity and directional changes of DAMs in roots and shoots under different treatments (**Figure 3a**).

Under SCN^−^ stress, the total number of root DAMs increased markedly with rising concentrations, from 289 (RC1/RC0, log2FC ranged from -6.5 to 10.2) to 839 (RC2/RC0, log2FC ranged from -10.8 to 10.2), with down-regulated DAMs becoming predominant in RC2/RC0 (513), reflecting severe metabolic suppression in roots under high-concentration stress. Conversely, shoot DAMs increased from 343 (SC1/SC0, log2FC ranged from -8.9 to 7.8) to 720 (SC2/SC0,log2FC ranged from -9.4 to 11.8), with up-regulated DAMs becoming dominant (476), indicating that shoots activated defensive metabolism to cope with stress.

H_2_S treatment alone predominantly induced moderate metabolic suppression in both roots (RT0/RC0, 261 down-regulated DAMs, log2FC ranged from -1.0 to -5.9) and shoots (ST0/SC0, 202 down-regulated DAMs, log2FC ranged from -1.0 to -8.9), establishing energy reserves and metabolic pre-adaptive states that laid the foundation for stress resistance.

After H_2_S alleviation treatments, total DAMs decreased substantially in both tissues. In roots, 205 DAMs were detected in the RT1/RC1 comparison (log2FC ranged from -7.2 to 8.2) and 299 in RT2/RC2 (log2FC ranged from -9.2 to 6.3). In shoots, 149 DAMs were found in ST1/SC1 (log2FC ranged from -9.0 to 11.9) and 223 in ST2/SC2 (log2FC ranged from -9.0 to 4.9). Notably, the number of down-regulated DAMs in roots decreased considerably, significantly attenuating the extreme metabolic fluctuations induced by stress and effectively reversing SCN^−^-mediated metabolic inhibition. Meanwhile, the proportion of up-regulated DAMs increased in shoots, activating defensive metabolic responses.

#### 3.3.2 Functional classification of DAMs

Further functional annotation and proportional analysis (**Figure 3b**; **Supplementary Table S2**) revealed the specific composition of DAMs.

SCN^−^ stress induced substantial down-regulation of lipids (6.6% in RC1/RC0; 13.1% in RC2/RC0), terpenoids (9.9%; 15.6%), and flavonoids (34.7%; 12.1%) in roots, while shoots exhibited predominant down-regulation of flavonoids (16.3% in SC1/SC0; 23.0% in SC2/SC0), phenolic acids (5.3%; 12.7%), and terpenoids (24.5%; 9.4%). These findings indicate that SCN^−^ severely disrupted both primary and secondary metabolism, particularly suppressing the synthesis of defense-related secondary metabolites.

H_2_S treatment alone enriched up-regulated DAMs in roots (RT0/RC0) with flavonoids (35.1%), phenolic acids (12.5%), and lipids (8.9%), notably including Tricin-4’-O-(β-guaiacylglycerol) ether-5-O-(6’’-malonyl)glucoside (log2FC=5.8, *P* = 0.016) and Isovitexin-2’’-O-xyloside (log2FC=7.2, *P* = 0.003). In shoots (ST0/SC0), up-regulated DAMs were enriched in flavonoids (32.4%), phenolic acids (19.1%), and terpenoids (13.2%), exemplified by Gardenin B (log2FC=5.1, *P* = 0.0006) (**Supplementary Table S3**). These results demonstrate that H_2_S treatment alone can activate multiple classes of defensive metabolites, establishing a preparatory state for stress response.

Under H_2_S alleviation treatment, the percentage of up-regulated lipids in roots increased from 7.1% in RC1/RC0 to 14.3% in RT1/RC1 and 11.3% in RC2/RC0 to 16.1% in RT2/RC2, respectively, while down-regulated lipids decreased from 6.6% and 13.1% to 5.0% and 10.5%; concurrently, up-regulated flavonoids rose from 23.8% and 24.2% to 39.3% and 40.7%, indicating that H_2_S enhanced lipid accumulation and fortified flavonoid-mediated defenses to meet energy demands and facilitate detoxification. Specific metabolites included the membrane lipid-related lysophosphatidylcholine (LysoPC 20:0, log2FC=4.6, *P* = 0.035), the energy metabolism-associated flavin adenine dinucleotide (FAD, log2FC=2.7, *P* = 0.009), and the flavonoid Tricin-4’-O-(syringyl alcohol) ether-7-O-glucoside (log2FC=2.6, *P* = 0.090) (**Supplementary Table S4**). The accumulation of these lipids provided direct material support for maintaining membrane stability and energy supply in roots.

In shoots, H_2_S increased the proportion of up-regulated flavonoids from 5.1% in SC1/SC0 to 19.6% in ST1/SC1 and 7.1% in SC2/SC0 to 10.0% in ST2/SC2, up-regulated phenolic acids from 4.6% in SC2/SC0 to 18.3% in ST2/SC2, and up-regulated terpenoids from 8.2% in SC1/SC0 to 34.4% in ST1/SC1, while reducing down-regulated terpenoids from 24.5% in SC1/SC0 to 10.0% in ST1/SC1. Key metabolites included Glucosyl-rhamnazin-3-O-β-D-glucoside (log2FC=11.9, *P* = 0.001), Neosakuranetin (log2FC=6.1, *P* = 0.004), Tricin-7-O-glucoside-4’-O-caffeoylglycerol (log2FC=5.4, *P* = 0.006), Mililatensol A (log2FC=2.5, *P* = 0.013), and geranyl acetate (log2FC=1.2, *P* = 0.006) (**Supplementary Table S5**). These compounds function as highly efficient reactive oxygen species (ROS) scavengers: flavonoids directly quench superoxide anions (O_2_^−^) and hydrogen peroxide (H_2_O_2_) through their phenolic hydroxyl groups, while terpenoids possess ROS-scavenging, antioxidant, and defense signaling activation properties. As critical secondary metabolites for plant responses to oxidative stress, terpenoids can activate downstream defensive metabolism including flavonoids and phenolic acids, thereby enhancing overall stress tolerance. These alterations demonstrate that H_2_S robustly stimulated the production of flavonoids, phenolic acids, and terpenoids—key stress-defensive secondary metabolites—thereby strengthening the antioxidant capacity of shoots.

#### 3.3.3 Overlap relationships and tissue-specific responses of DAMs

Venn diagram intersection analysis further dissected the overlap and specificity relationships of DAMs among different treatments (**Figure 3c**). SCN^−^ stress exhibited pronounced dose-dependency and tissue specificity. In roots, 79 and 521 DAMs were identified at EC_20_ (RC1/RC0) and EC_50_ (RC2/RC0), respectively, with 115 shared between concentrations; in shoots, 80 and 440 DAMs were detected, with 131 shared.

Regarding the H_2_S alleviating effect, roots contained 23 core DAMs (RT1/RC1 ∩ RT2/RC2) regulated by H_2_S at both concentrations, predominantly enriched in organic acids, lipids, and flavonoids, including triethyl citrate (log2FC=1.1, *P* = 0.006), 2-aminooctadecane-1,5,7,17-tetraol (log2FC=1.4, *P* = 0.002), and Tricin-4’-O-(syringyl alcohol) ether-7-O-glucoside (log2FC=3.5, *P* = 0.005). Shoots contained 21 core DAMs (ST1/SC1 ∩ ST2/SC2), primarily belonging to terpenoids and phenolic acids, including geranyl acetate (log2FC=1.2, *P* = 0.006) and benzoyl-β-D-glucoside (log2FC=4.2, *P* = 0.018) (**Table 1**). These findings indicate that H_2_S coordinately alleviates stress by stabilizing root membrane systems, ensuring energy supply and enhancing shoot antioxidant capacity.

**Table 1:** Core DAMs in the root and shoot under H_2_S+SCN^−^ treatment.

Class	Name	KeggID	ST1/SC1		ST2/SC2	
			P-value	Log2FC	P-value	Log2FC
Shoot						
Alkaloids	1-O-p-Coumaroyllysine	–	0.003	-2.45	0.009	-1.39
	6,7-Dimethoxy-2-benzoxazolinone (DMBOA)	–	0.035	-1.17	0.011	-1.14
Lignans and Coumarins	(+)-Lyoniresinol 9'-O-glucoside	–	0.000	1.41	0.005	4.01
Lipids	(E)-12-hydroxydodec-2-enoic acid	–	0.002	1.57	0.010	1.87
	Arachidonate	C00219	0.010	1.49	0.008	2.77
	Panaxytriol	C16792	0.015	-2.57	0.002	-2.68
Others	2-Hydroxy-7-carboxy-1-methyl-5-ethenyl-9,10-dihydrophenanthrene	–	0.000	-1.90	0.013	1.30
	2-Hydroxy-8-carboxy-1-methyl-5-ethenyl-9,10-dihydrophenanthrene	–	0.001	-1.92	0.004	1.36
	3-(1-hydroxyethyl)-4-methylpentane-1,4-diol O-Glucoside	–	0.028	3.41	0.002	3.93
	3'-Norspongiolactone	–	0.001	-8.57	0.007	4.17
	6-phenyl-hexan-2-ol	–	0.000	1.53	0.015	1.30
	Gentisate aldehyde	C05585	0.044	-3.25	0.041	-3.26
Phenolic acids	4-O-Feruloyl Aminogalactitol	–	0.009	-4.23	0.006	-1.23
	Benzoyl-Beta-D-Glucoside	–	0.018	4.21	0.021	2.52
	Bilobol	C10770	0.001	-9.06	0.004	4.94
Terpenoids	[(4aR,5R,8aS)-1-(hydroxymethyl)-4a-methyl-6-methylidene-5-[(2E)-3-methylpenta-2,4-dien-1-yl]-decahydronaphthalen-1-yl]methanol	–	0.046	2.74	0.013	2.96
	2,3,3a,4,5,6-Hexahydro-1,4-dimethylazulen-4-ol	–	0.000	1.60	0.001	1.79
	3,19-Epoxy-3,22-dihydroxydammara-20,24-dien-26-oic acid δ-lactone (Semialactone)	–	0.014	3.22	0.006	3.34
	3-Acetoxy-9,13-epoxy-16-hydroxy-labda-15,16-olide	–	0.049	-2.85	0.006	3.11
	Geranyl acetate	C09861	0.007	1.25	0.017	1.60
	Soyasapogenol E	C17420	0.011	2.60	0.040	-3.42
Root						
Flavonoids	4'-(beta-D-glucopyranosyloxy)-5-hydroxy-3,3',7-trimethoxyflavone	–	0.008	-2.53	0.031	-3.08
	4',5,8-Trihydroxyflavanone	–	0.032	-2.87	0.002	2.83
	Acacetin-7-O-neohesperidoside	–	0.040	1.04	0.008	1.71
	Apigenin-8-C-glucoside-7-O-Sophoroside	–	0.018	4.09	0.041	-3.93
	Isoscoparine	C05990	0.027	-2.56	0.042	-2.91
	Quercetin-sinapyl-glucoside	–	0.041	-2.98	0.045	3.21
	Tricin-4'-O-(syringyl alcohol)ether-7-O-glucoside	–	0.005	3.54	0.002	-1.24
Lignans and Coumarins	4-Hydroxycoumarin	C20414	0.036	-1.35	0.001	2.97
	Bursehernin	C21183	0.018	2.72	0.026	-2.80
	Cichoriin	C09206	0.019	3.57	0.025	4.00
	Fraxidinglucoside	–	0.018	4.06	0.002	1.81
Lipids	2-Aminooctadecane-1,16,18,18-tetraol	–	0.003	1.50	0.000	1.61
	2-Aminooctadecane-1,5,7,17-tetraol	–	0.003	1.47	0.026	-1.27
	PS(18:3(6Z,9Z,12Z)/0:0)	–	0.036	-1.94	0.000	-1.55
Nucleotides and derivatives	Thymine	C00178	0.000	-1.24	0.005	1.65
Organic acids	Triethyl citrate	–	0.006	1.19	0.025	2.91
Others	3,4-Dihydroxyphenethyl alcohol-8-O-[4-O-caffeoyl-β-D-apinosyl(1→3)-β-D-glucosyl(1→6)]-β-D-glucoside	–	0.021	2.71	0.008	3.08
	5,8-dihydroxy-1-hydroxymethylnaphtho[2,3-c]furan-4,9-dione	–	0.039	-3.30	0.025	2.75
Phenolic acids	1,5-O-dicaffeoyl-3-O-dimethylmalyl-quinic acid	–	0.042	-2.89	0.019	-2.66
	4-O-(4'-O-alpha-D-Glucopyranosyl)caffeoylquinic acid	–	0.001	-3.05	0.002	-1.64
Quinones	2,5-dihydroxy-1-methoxy-anthraquinone	–	0.000	-1.75	0.013	3.28
Terpenoids	alpha-Ionone	C12286	0.005	-2.43	0.012	2.62
	Aquilariaene F	–	0.027	3.94	0.047	2.82

Analysis of the relationship between H_2_S treatment alone and alleviating effects revealed that 35 and 39 root DAMs from the alleviating effects overlapped with H_2_S treatment alone, with 45 DAMs common to all three comparisons; shoots shared 60 and 29 DAMs, respectively, with 12 common to all three. Functional analysis showed that these shared metabolites were dominated by lipids, terpenoids, and phenolic acids in roots, and flavonoids, phenolic acids, and terpenoids in shoots (**Supplementary Table S6**), demonstrating that H_2_S priming alone can activate secondary metabolic potential.

Furthermore, H_2_S-specific alleviatory metabolites (appearing exclusively in T1/C1 or T2/C2, **Figure 3c**, unique regions of RT1/RC1 and RT2/RC2; ST1/SC1 and ST2/SC2) represented stress-induced specific defensive substances: roots were enriched in lipids and flavonoids associated with cell wall modification; shoots contained predominantly complex flavonoids, terpenoid derivatives, and phenolic acids that play pivotal roles in direct ROS scavenging. Collectively, these results demonstrate that H_2_S systematically reconstructs carbon-nitrogen metabolic networks through tissue-specific mechanisms—reinforcing energy and membrane-stabilizing metabolism in roots while driving antioxidant secondary metabolism in shoots.

### 3.4 KEGG pathway enrichment analysis reveals the core metabolic pathway affected by H_2_S

**Figure 4:**
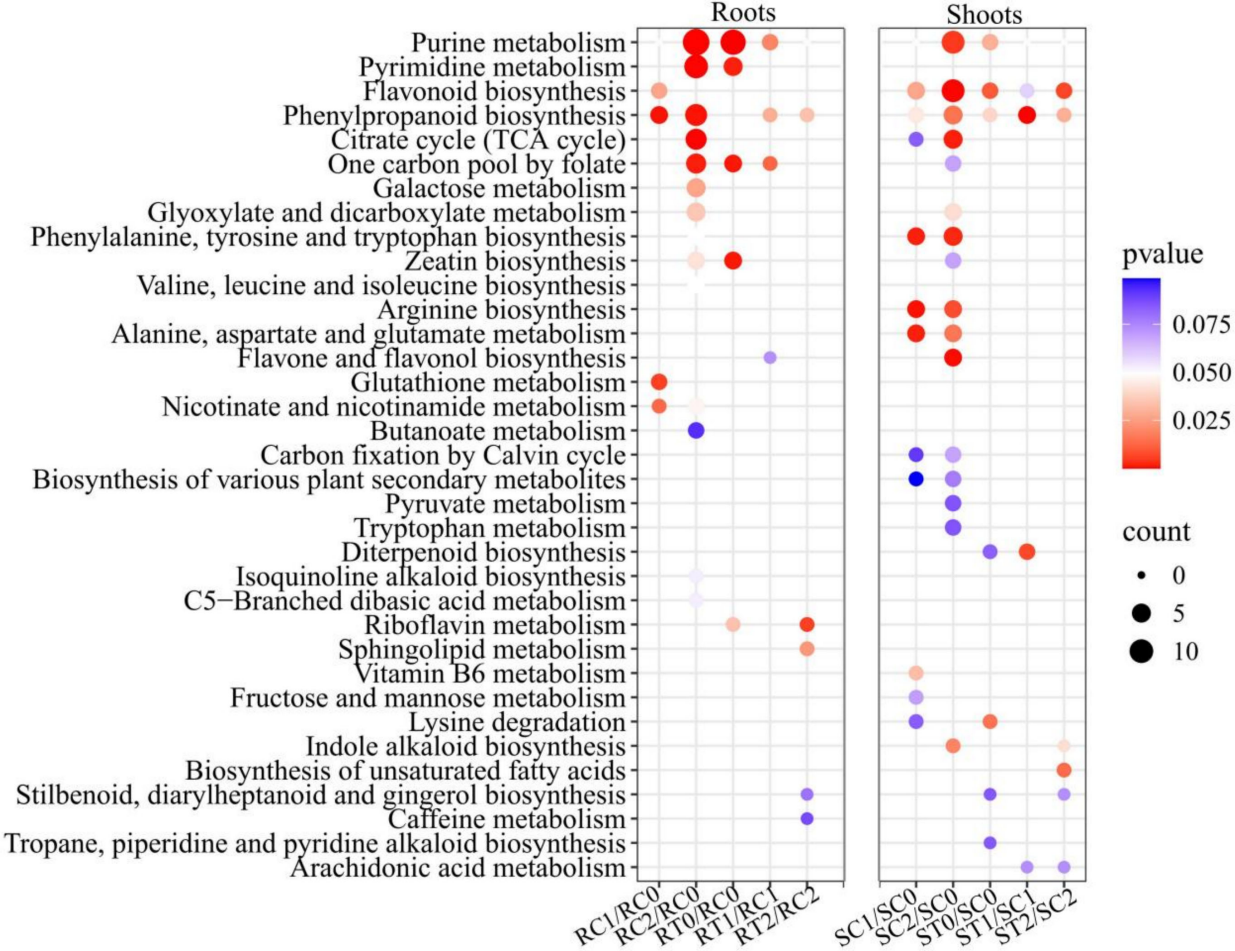
Core metabolic pathways regulated by stress effect (C1/C0 and C2/C0), H_2_S-specific effect (T0/C0) and alleviating effect (T1/C1 and T2/C2). KEGG pathway enrichment matrix, the closer the P-value is to zero (red), the more significant the enrichment. Dot size in the plot indicates the number of DAMs mapped to each pathway. C1/C0: represent C1 vs C0, and so on for the others.

KEGG enrichment analysis revealed tissue-specific metabolic networks modulated by H_2_S (**Figure 4**). In roots, SCN^−^ stress significantly enriched pathways including purine metabolism, the TCA cycle, and phenylpropanoid biosynthesis. H_2_S treatment alone (RT0/RC0) activated purine metabolism (*P* = 0.001), pyrimidine metabolism (*P* = 0.001), one-carbon pool by folate (*P* = 0.001), zeatin biosynthesis (*P* = 0.001), and riboflavin metabolism (*P* = 0.034). Under H_2_S alleviation at EC_20_ (RT1/RC1), purine metabolism (*P* = 0.019), one-carbon pool by folate (*P* = 0.012), and phenylpropanoid biosynthesis (*P* = 0.029) were significantly enriched; at EC_50_ (RT2/RC2), enrichment shifted toward riboflavin metabolism (*P* = 0.005) and sphingolipid metabolism (*P* = 0.023), with TCA cycle-associated metabolites being maintained. Riboflavin metabolism provides the essential cofactor FAD for glutathione reductase, while sphingolipid metabolism participates in membrane stability maintenance and stress signal transduction. These findings indicate that H_2_S alleviates stress in roots by ensuring energy supply and membrane stability.

In shoots, SCN^−^ stress significantly enriched defense-related pathways including flavonoid biosynthesis, phenylpropanoid biosynthesis, and amino acid biosynthesis. H_2_S treatment alone (ST0/SC0) activated purine metabolism (*P* = 0.029), flavonoid biosynthesis (*P* = 0.009), lysine degradation (*P* = 0.015) and phenylpropanoid metabolism (*P* = 0.039). Under H_2_S alleviation, phenylpropanoid biosynthesis (*P* = 0.0001) and diterpenoid biosynthesis (*P* = 0.007) were significantly enriched at EC20 (ST1/SC1); at EC_50_ (ST2/SC2), flavonoid biosynthesis (*P* = 0.006), biosynthesis of unsaturated fatty acids (*P* = 0.013), phenylpropanoid biosynthesis (*P* = 0.029), and indole alkaloid biosynthesis (*P* = 0.041) were significantly enriched. These results demonstrate that H_2_S enhances antioxidant capacity by systematically activating the defensive metabolic network in shoots.

### 3.5 WGCNA identifies H_2_S-associated C and N coexpression modules

**Figure 5:**
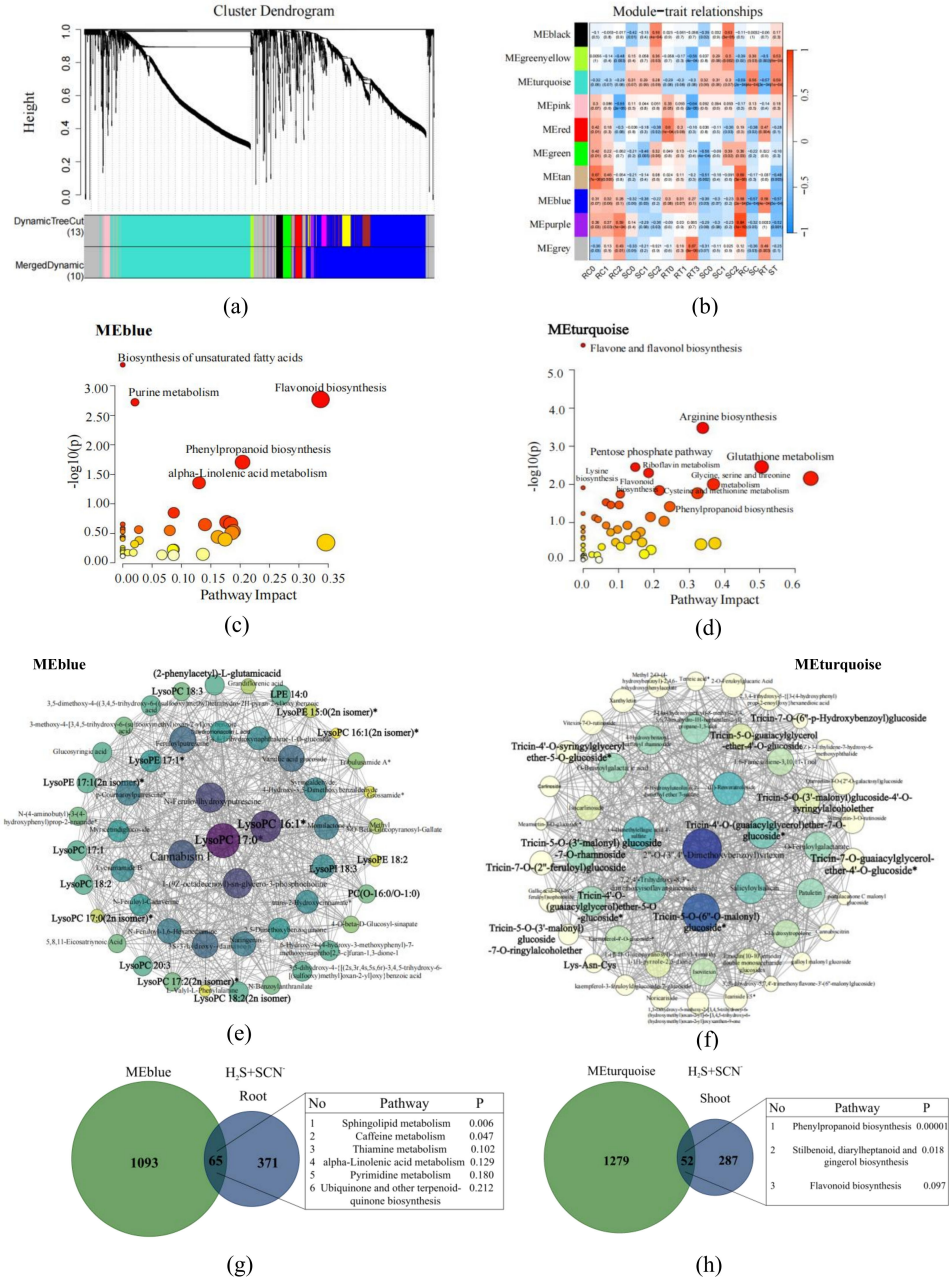
Carbon-Nitrogen co-expression metabolic modules of H_2_S regulation. **(a)** Module identification and analysis. The upper panel shows the metabolites clustering tree constructed from the dissTOM matrix based on the weighted correlation coefficients. The lower panel displays the distribution of metabolites within each module, with the same color indicating the same module. **(b)** Trait-module association heatmap. **(c, d)** KEGG pathway mapping of metabolites within the MEblue and MEturquoise modules, respectively. **(e, f)** Network of the top 50 most highly connected metabolites within the MEblue and MEturquoise modules, respectively. **(g, h)** Venn diagrams show the overlap between metabolites in the MEblue and MEturquoise modules and the root- and leaf-specific DAMs, respectively.

To examine the coordinated changes in metabolite abundance under H_2_S, we constructed a weighted gene coexpression network (WGCNA) using all the quantified metabolites. Ten distinct coexpression modules were identified (**Figures 5a, b**; **Supplementary Figure S5**; **Supplementary Tables S7**, **S8**). MEblue (1,158 metabolites) was significantly positively correlated with root samples (*r* = 0.56, *P* = 0.00038) and significantly negatively correlated with shoot samples (*r* = -0.57, *P* = 0.00029) from H_2_S+SCN^−^ treatment plants. The metabolites in this module were significantly enriched in lipid metabolism, nucleotide metabolism, and the biosynthesis of other secondary metabolites (**Figure 5c**). These findings indicate that MEblue copes with stress by modulating the energy supply and membrane stability in the roots. In contrast, MEturquoise (1,331 metabolites) exhibited the opposite pattern, with a significant positive correlation with shoots (*r* = 0.59, *P* = 0.00014) and a significant negative correlation with roots (*r* = -0.57, *P* = 0.00032). Its metabolites were significantly enriched in amino acid metabolism, carbohydrate metabolism, secondary metabolite biosynthesis and cofactor/vitamin metabolism (**Figure 5d**). MEturquoise was associated with coordinated C and N metabolism in the shoot and with the synthesis of stress-defensive secondary metabolites.

Analysis of the 50 most highly connected hub metabolites within each module (**Figures 5e, f**) further revealed these tissue-specific patterns. The MEblue hub metabolites were predominantly lipids (e.g., lysophospholipids), whereas the MEturquoise hub metabolites were primarily flavonoids (e.g., tricin derivatives). This pattern is consistent with the observed prevalence of lipid related metabolites in roots and flavonoid associated metabolites in shoots under H_2_S regulation.

By identifying the overlap between metabolites in the WGCNA modules (MEblue, MEturquoise) and the DAMs from Section 3.3, we obtained 65 metabolites common to the root-associated module and DAMs and 52 metabolites common to the shoot-associated module and DAMs. Pathway analysis revealed that the 65 root-overlapping metabolites were significantly enriched in sphingolipid metabolism (*P* = 0.006) and caffeine metabolism (*P*=0.047). The 52 shoot-overlapping metabolites were enriched in phenylpropanoid biosynthesis (*P* = 0.00001), stilbenoid, diarylheptanoid and gingerol biosynthesis (*P* = 0.018) and flavonoid biosynthesis (*P*=0.097) (**Figures 5g, h**). These overlapping metabolites and their enriched pathways represent potential functional units that are both responsive to H_2_S and exhibit coordinated abundance changes.

## 4 Discussion

### 4.1 H_2_S synergistically regulates root C and N metabolism to drive defense enhancement

Plant roots are in direct contact with the environment and serve as critical organs for the entry of pollutants into plants. Studies have shown that SCN^−^ is susceptible to absorption by roots, causing membrane structure damage and energy metabolism disorders (Lin et al., 2020). Our findings revealed that root CA and NA in rice seedlings decreased under SCN^−^ treatment, whereas both parameters remained stable at control levels under H_2_S+SCN^−^ treatment, with root C/N ratio recovering to near-control levels (**Figure 1**). These findings indicate that H_2_S alleviates stress-induced metabolic disorder by modulating C and N metabolic balance in roots. This maintenance of C and N homeostasis provides fundamental support for the normal operation of downstream primary and secondary metabolism, enabling roots to sustain membrane stability, continuous energy supply, and transmembrane substance transport under SCN^−^ stress. Specifically, the stabilization of CA supplies nonstructural carbohydrates and lipids for energy metabolism (Geigenberger et al., 2005), whereas increased NA ensures the provision of N sources for the synthesis of repair-related biomacromolecules such as nucleic acids and proteins (Xing et al., 2023). For example, H_2_S+SCN^−^ treatment significantly increased the proportion of root lipids, especially membrane components such as lysophospholipids (**Figure 3c**). Notably, LysoPC 20:0 was up-regulated 4.6-fold, indicating that H_2_S maintains membrane system integrity by promoting membrane lipid synthesis. In fact, lipids are core components of biological membranes and play dual key roles in stress resistance: on the one hand, they maintain membrane fluidity and integrity, enhancing the physical resistance of roots to adverse environments (Jin et al., 2022; Ren et al., 2025) and reducing the transmembrane transport of SCN^−^; on the other hand, lipid degradation products can enter the TCA cycle via β-oxidation, providing energy support for detoxification processes (Lu et al., 2025).

The specific activation of metabolic pathways is a crucial molecular strategy for plants to cope with stress. KEGG enrichment analysis revealed that H_2_S optimization of root metabolism is manifested, on one hand, through precise activation of energy and genetic material repair pathways. Both H_2_S treatment alone (RT0/RC0) and H_2_S alleviation treatments (RT1/RC1, RT2/RC2) were significantly enriched in purine metabolism and one-carbon pool by folate (**Figure 4**). From the perspective of C/N metabolic coupling, this represents a synergistic process. Through one-carbon pool by folate and associated pathways, plants may act as a carbon sink to consume excess photosynthetic products, thereby addressing C/N ratio imbalances (Zhou et al., 2023). As a central hub for C and N coupling, purine metabolism not only supplies nucleotide precursors for DNA repair and protein synthesis, but its metabolic intermediates (ATP, ADP, AMP) serve as critical carriers for cellular energy transduction, forming a closed-loop energy metabolic network together with the TCA cycle (Ghorbanzadeh et al., 2023; Cao et al., 2023) that collectively constitutes the metabolic foundation for root stress resistance. Concurrently, purine ring synthesis requires N atoms provided by glutamine, glycine, and other amino acids, with its metabolic activity directly reflecting the degree of C and N metabolic coupling (Coleto et al., 2016). H_2_S treatment alone enhanced purine metabolism, reserving necessary metabolic potential for energy demands and nucleic acid repair under stress, which aligns closely with its physiological performance in maintaining CA and NA in roots (**Figure 1**). On the other hand, H_2_S also reinforced the physical barrier and redox buffering capacity of the root system. H_2_S significantly up-regulated multiple membrane lipid components including LysoPC 20:0, and activated sphingolipid metabolism and riboflavin metabolism pathways. Lipids are core constituents of cell membranes, and their synthesis depends on fatty acid chains supplied by C metabolism (Jin et al., 2022). Sphingolipids serve not only as structural membrane components but also participate in stress signal perception and transduction (Rodrigues et al., 2021). More importantly, the product of riboflavin metabolism—FAD—was significantly up-regulated in the RT2/RC2 group (log2FC=2.7), functioning as an essential cofactor for glutathione reductase. C metabolism provides ribitol and aromatic ring precursors for FAD synthesis, while N metabolism contributes to the construction of its adenine moiety. The accumulation of FAD directly enhances ROS scavenging capacity in roots, thereby protecting membrane structural integrity at the subcellular level and alleviating stress-induced oxidative damage.

WGCNA further revealed the correlation between C and N metabolism. The core metabolites of the MEblue module in roots are predominantly lipids and nucleotides, which are significantly enriched in pathways such as alpha-linolenic acid metabolism and purine metabolism (**Figure 5c**). The activation of these pathways relies on acyl chains and glycolytic intermediates (e.g., phosphoenolpyruvate, PEP) provided by C metabolism, as well as amino and purine ring structures supplied by N metabolism (Wang et al., 2024a, 2024b; Zi et al., 2022). Previous studies have confirmed that H_2_S can maintain ATP levels under stress by regulating the activity of key energy metabolism enzymes (Zhu et al., 2022; Huang et al., 2023). H_2_S treatment improves the energy metabolism status of rice seedlings under salt stress by increasing the expression of ATP synthesis-related proteins (e.g., malate dehydrogenase) (Wei et al., 2021). Similarly, H_2_S pretreatment enhances the stress resistance of rice under aluminum toxicity by increasing ATP and nonstructural carbohydrate contents (Zhu et al., 2022). In this study, the direct allocation of root C and N to energy metabolism and repair-related pathways represents a specific manifestation of this mechanism in tissue-specific metabolic networks. This ultimately ensures the normal absorption of water and nutrients by maintaining root cell homeostasis, thereby providing support for aboveground growth.

### 4.2 H_2_S synergistically regulates shoot C and N metabolism to drive defense enhancement

Unlike roots, shoots serve as core sites for photosynthesis (Qin et al., 2024; Ni et al., 2020), and their metabolic reprogramming focuses more on enhancing defense functions. Under H_2_S+SCN^−^ treatment, shoots maintained relatively high CA and NA levels, preventing substantial nutrient loss, and the shoot C/N ratio decreased at the EC_50_ (**Figure 1**). This physiological change indicates a more prominent increase in NA in shoots, reflecting the preferential allocation of N resources to shoots and providing a material prerequisite for the synthesis of N-containing defense compounds. Studies have shown that SCN stress inhibits the function of rice PSII, leading to reduced C assimilation efficiency (Yang et al., 2021). Moreover, H_2_S ensures the continuous progress of photosynthetic C fixation and provides sufficient N sources for secondary metabolite synthesis by stabilizing shoot CA and NA (Zhang et al., 2024). For example, under H_2_S+SCN^−^ treatment, the proportion of flavonoids, phenolic acids, and terpenoids among DAMs in shoots increased significantly—flavonoids increased from 5.1% to 19.6%, phenolic acids from 4.6% to 18.3%, and terpenoids increased from 8.2% to 34.4%, while down-regulated terpenoids decreased from 24.5% to 10.0% (**Figure 3c**).

KEGG analysis (**Figure 4**) revealed that, compared with the SCN^−^ treatment, H_2_S treatment alone (ST0/SC0) activated purine metabolism (*P* = 0.029), flavonoid biosynthesis (*P* = 0.009), lysine degradation (*P* = 0.015) and phenylpropanoid metabolism (*P* = 0.039), laying the groundwork for subsequent stress responses. Under H_2_S alleviation, phenylpropanoid biosynthesis (*P* = 0.0001), diterpenoid biosynthesis (*P* = 0.007), flavonoid biosynthesis (*P* = 0.006), were the most significantly enriched pathways, establishing systematic activation of the defensive metabolic network. As typical stress-resistant secondary metabolites in plants, flavonoids and terpenoids exhibit strong antioxidant and detoxification activities: they not only effectively scavenge ROS to protect the photosynthetic machinery from oxidative damage but also strengthen cell wall structures and regulate signaling pathways to increase tissue stress resistance (Zhuang et al., 2024; Shamala et al., 2020; Sugimoto et al., 2021). Their synthesis relies on precursors such as phenylalanine and acetyl-CoA provided by C metabolism, as well as amino and methyl donors (e.g., S-adenosylmethionine) supplied by N metabolism (Shamala et al., 2020; Sugimoto et al., 2021). H_2_S treatment alone stabilized CA and NA in shoots, furnishing ample substrates for the operation of these pathways. Concurrently, the sustained activation of phenylpropanoid biosynthesis and flavonoid biosynthesis pathways in KEGG enrichment results directly reflects effective C flux redirection toward defensive product synthesis. The substantial increase in the proportion of up-regulated flavonoids and terpenoids shown in **Figure 3c** corroborates this C and N coordinated defense enhancement at the metabolite level.

The core metabolites of the MEturquoise module further revealed the synergistic characteristics of the shoot metabolic network (**Figures 5d, f**). Its core metabolites are predominantly flavonoids, which are significantly enriched in flavonoid biosynthesis and phenylpropanoid biosynthesis pathways. This result reveals a synergistic regulatory network of shoot C and N metabolism: intermediates generated from photosynthetic products via glycolysis and the pentose phosphate pathway in C metabolism provide C skeletons for phenylpropanoid and flavonoid synthesis (Yang et al., 2023); N metabolism supplies N elements for the modification and assembly of secondary metabolites through the synthesis of amino acids such as glutamate and aspartate (Mamta et al., 2026; Asgher et al., 2022). This defense metabolic enhancement driven by C and N synergy makes the shoot as a key site for H_2_S-mediated stress resistance. Shoots not only scavenge ROS via antioxidant compounds but also reduce SCN^−^ accumulation through detoxification metabolism, ultimately ensuring plant photosynthetic efficiency and growth vigor.

### 4.3 Limitation analysis

**Figure 6:**
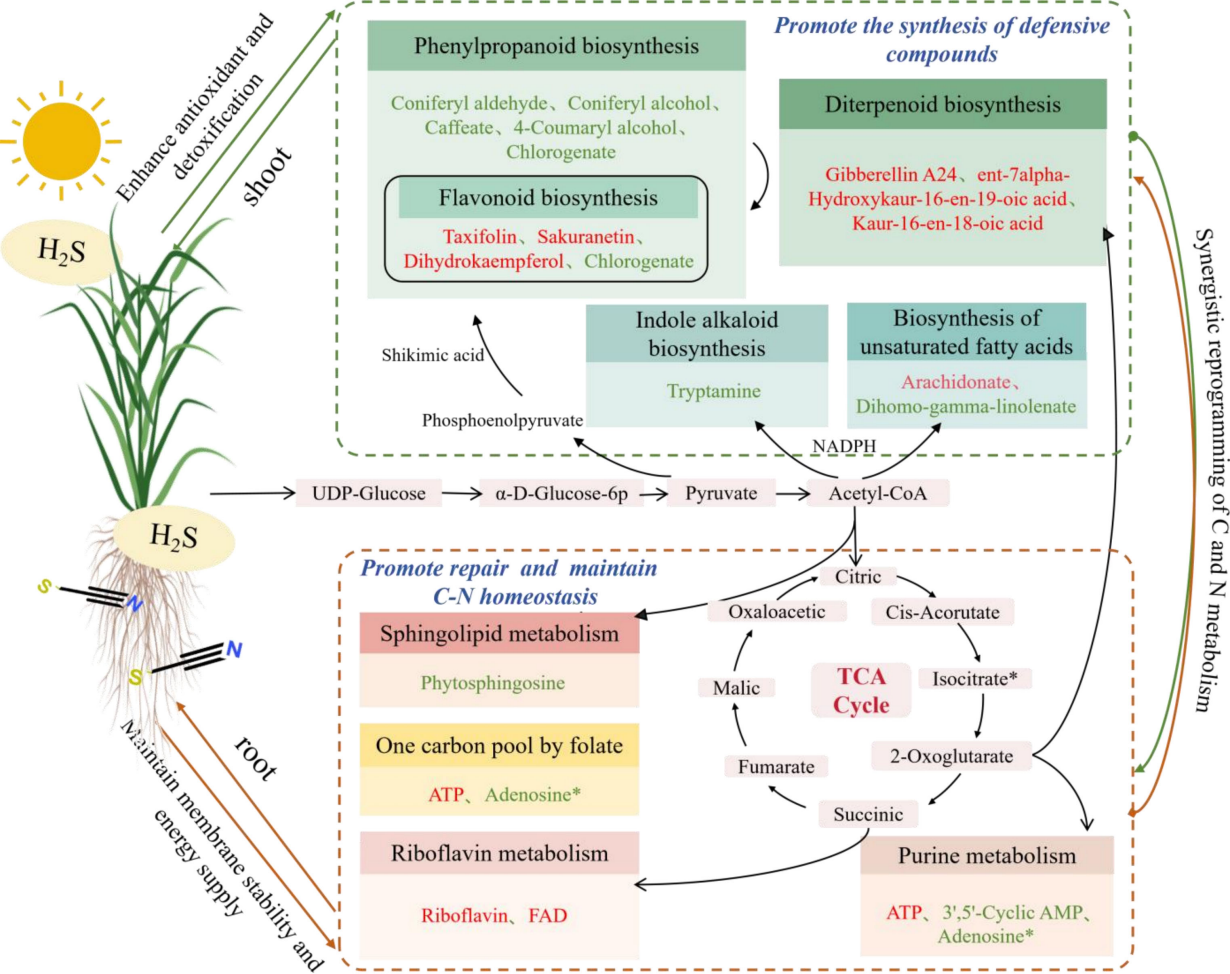
Tissue-specific reprogramming of carbon and nitrogen metabolism by exogenous H_2_S under SCN^-^ stress in rice seedlings. The mapping illustrates the core metabolic pathways regulated by H_2_S. Pathways significantly enriched in shoots are enclosed in green dashed boxes, while those in roots are in orange dashed boxes.The red text represents up-regulated DAMs, and green text represents down-regulated DAMs.

By integrating physiological, metabolomic and WGCNA, this study preliminarily elucidates the mechanism by which H_2_S regulates tissue-specific C and N metabolism to alleviate SCN stress in rice. As illustrated in **Figure 6**, we constructed a core metabolic pathway model that visually illustrates H_2_S-mediated C and N metabolic reprogramming. In this model, H_2_S functions to maintain C and N homeostasis in both roots and shoots, providing the material foundation for downstream defense responses. In roots(orange dashed box), H_2_S primarily activates an energy supply and repair network centered on purine metabolism and riboflavin metabolism. On one hand, enhanced purine metabolism furnishes N sources and C skeletons for DNA repair and ATP synthesis, cooperating with the TCA cycle to secure energy supply under stress. On the other hand, activated riboflavin metabolism provides the essential cofactor FAD for the antioxidant system, which together with up-regulated sphingolipid metabolism and lipid metabolism, collectively maintains membrane integrity, establishing physical and biochemical defense lines against SCN^−^. In shoots (green dashed box), H_2_S drives a defense network centered on phenylpropanoid biosynthesis, flavonoid metabolism and terpenoid biosynthesis. Photosynthetic products supply ample C skeletons and reducing power via C metabolism (e.g., the shikimate pathway) for the synthesis of these defensive compounds; concurrently, N metabolism contributes amino groups and methyl donors for their structural and functional modification and activation. The resulting massive synthesis of flavonoids and terpenoids cooperatively constitutes an efficient ROS scavenging system that protects photosynthetic machinery and enhances overall tissue detoxification capacity. This spatial divergence underscores how H_2_S orchestrates a systemic reconfiguration of the C and N metabolic network to increase stress tolerance in a tissue-specific manner.

However, several limitations remain to be addressed in future research. First, the causal relationship between H_2_S and the regulation of C and N metabolism requires further verification. For example, H_2_S synthesis mutants or CRISPR gene editing technology can be used to clarify regulatory pathways at the transcriptional or posttranslational level (Aroca et al., 2021). Second, the lack of direct measurements of C and N metabolic fluxes limits the precise understanding of C and N allocation efficiency and dynamic flow direction. In this study, C and N allocation was indirectly reflected by the C/N ratio and metabolite accumulation, but it was impossible to quantify the allocation rate of photosynthetic C to different metabolic pathways or the conversion efficiency of N from absorption to metabolite synthesis. Future research should combine ^13^C/^15^N dual isotope tracing technology to monitor the regulatory effects of H_2_S on C and N metabolic fluxes in real time under stress (Jung et al., 2025) and clarify the importance of C and N allocation between energy metabolism and defense metabolism. Despite these limitations, this study provides an important basis for understanding the H_2_S-mediated metabolic mechanisms of plant stress resistance. The identified potential biomarkers and tissue-specific metabolic pathways offer theoretical support for subsequent targeted regulation of C and N metabolism and breeding of stress-resistant crop varieties.

## 5 Conclusions

This study demonstrated that H_2_S enhances rice seedling tolerance to SCN^−^ stress through systematic reconfiguration of C and N metabolic networks. By integrating widely-targeted metabolomics with WGCNA, we revealed tissue-specific metabolic adjustments: roots primarily secure energy supply and membrane system stability under stress by activating an energy provision and repair network centered on purine metabolism and riboflavin metabolism, thereby laying the material foundation for the stress resistance response of the entire plant.; shoots, conversely, significantly enhance antioxidant defense capacity by driving phenylpropanoid biosynthesis, flavonoid metabolism, diterpenoid biosynthesis, biosynthesis of unsaturated fatty acids, and indole alkaloid biosynthesis. Metabolites such as lysophospholipids, tricin derivatives, and terpenoids represent potential biomarkers for stress diagnosis. Future research should employ ^13^C/^15^N isotopic flux analysis combined with genetic approaches to quantify C and N partitioning and elucidate the regulatory mechanisms controlling these metabolic processes. Such mechanistic insights will facilitate the development of H_2_S-based strategies for the remediation of SCN^−^-contaminated agricultural systems and contribute to the breeding of stress-resistant crop varieties.

## Data Availability

The original contributions presented in the study are included in the article/**Supplementary Material**. Further inquiries can be directed to the corresponding authors.
