# Supplementary material for: Hydrogen sulfide alleviates thiocyanate stress in rice seedlings via tissue-specific carbon and nitrogen metabolic reprogramming
Source: Front Plant Sci. 2026 Apr 27;17:1769534. doi: 10.3389/fpls.2026.1769534 (PMC13158110; doi:10.3389/fpls.2026.1769534)
Supplement: Supplementary file 1 [file Supplementaryfile1.docx]

***Supplementary Material for***

**Hydrogen sulfide alleviates thiocyanate stress in rice seedlings via tissue-specific carbon and nitrogen metabolic reprogramming**

Meng-Hua Chen^a^, Hui-Lin Chen^a^, Yu-Xi Feng^b*^, Yu-Juan Lin^a,c,d^, Yan-Hong Li^a,c,d*^

*^a^ College of Environmental Science and Engineering, Guilin University of Technology, Guilin 541004, China*

*^b^ Guangdong-Hong Kong Joint Laboratory for Carbon Neutrality, Jiangmen Laboratory of Carbon Science and Technology, Jiangmen 529199, China*

*^c^ Guangxi Key Laboratory of Environmental Pollution Control Theory and Technology, Guilin University of Technology, Guilin 541006, China*

*^d^ Engineering Research Center of Watershed Protection and Green Development, University of Guangxi, Guilin University of Technology, Guilin 541006, China*

**^*^Corresponding author**

Yu-Xi Feng. E-mail address: [yu-xifeng@foxmail.com](mailto:yu-xifeng@foxmail.com)

Yan-Hong Li. E-mail address: lyh1685@163.com

**Table S1:** R^2^Y and Q^2^ values of the PLS-DA

| Treatment | Comparison group | R^2^ Y | Q^2^ |
| --- | --- | --- | --- |
| SCN⁻ treatment | RC1/RC0 | 1 | 0.954 |
|  | RC2/RC0 | 1 | 0.983 |
|  | SC1/SC0 | 1 | 0.914 |
|  | SC2/SC0 | 1 | 0.960 |
| H_2_S + SCN⁻ treatment | RT0/RC0 | 1 | 0.959 |
|  | RT1/RC0 | 1 | 0.960 |
|  | RT2/RC0 | 1 | 0.984 |
|  | ST0/SC0 | 1 | 0.907 |
|  | ST1/SC0 | 1 | 0.893 |
|  | ST2/SC0 | 1 | 0.957 |

1. RC0、RC1、RC2、SC0、SC1、SC2: Represent the roots and shoots of rice seedlings subjected to stress for 3 days in stress solutions with SCN^-^ concentrations of 0 (C0), 24.0 (C1), and 96.0 (C2) mg/L, respectively.
2. RT0、RT1、RT2、ST0、ST1、ST2: Represent the roots and shoots of rice seedlings that were pre-treated with exogenous H_2_S solution for 6 hours and then subjected to stress for 3 days in stress solutions with SCN concentrations of 0 (C0), 24.0 (C1), and 96.0 (C2) mg/L, respectively.

**Table S2: Functional classification proportion of DAMs**

| Metabolite category | Upregulated metabolites | | | | | Downregulated metabolites | | | | |
| --- | --- | --- | --- | --- | --- | --- | --- | --- | --- | --- |
|  |  |  |  |  |  |  |  |  |  |  |
| **Shoot** | **SC1/SC0** | **SC2/SC0** | **ST0/SC0** | **ST1/SC1** | **ST2/SC2** | **SC1/SC0** | **SC2/SC0** | **ST0/SC0** | **ST1/SC1** | **ST2/SC2** |
| Alkaloids | 17 (17.3%) | 116 (24.4%) | 6 (8.8%) | 20 (12.3%) | 5 (8.3%) | 33 (13.5%) | 10 (4.1%) | 22 (10.9%) | 8 (13.3%) | 13 (14.6%) |
| Amino acids and derivatives | 17 (17.3%) | 70 (14.7%) | 3 (4.4%) | 5 (3.1%) | 1 (1.7%) | 25 (10.2%) | 10 (4.1%) | 11 (5.4%) | 2 (3.3%) | 6 (6.7%) |
| Flavonoids | 5 (5.1%) | 34 (7.1%) | 22 (32.4%) | 32 (19.6%) | 6 (10.0%) | 40 (16.3%) | 56 (23.0%) | 28 (13.9%) | 13 (21.7%) | 21 (23.6%) |
| Lignans and Coumarins | 8 (8.2%) | 16 (3.4%) | 2 (2.9%) | 9 (5.5%) | 3 (5.0%) | 6 (2.4%) | 21 (8.6%) | 3 (1.5%) | 3 (5.0%) | 2 (2.2%) |
| Lipids | 6 (6.1%) | 51 (10.7%) | 4 (5.9%) | 13 (8.0%) | 6 10.0%) | 10 (4.1%) | 7 (2.9%) | 19 (9.4%) | 2 (3.3%) | 10 (11.2%) |
| Nucleotides and derivatives | 4 (4.1%) | 16 (3.4%) | 0 (0.0%) | 0 (0.0%) | 1 (1.7%) | 0 (0.0%) | 7 (2.9%) | 10 (5.0%) | 1 (1.7%) | 2 (2.2%) |
| Organic acids | 2 (2.0%) | 6 (1.3%) | 1 (1.5%) | 3 (1.8%) | 1 (1.7%) | 9 (3.7%) | 21 (8.6%) | 1 (0.5%) | 2 (3.3%) | 1 (1.1%) |
| Others | 11 (11.2%) | 38 (8.0%) | 8 (11.8%) | 15 (9.2%) | 13 (21.7%) | 45 (18.4%) | 55 (22.5%) | 39 (19.3%) | 14 (23.3%) | 8 (9.0%) |
| Phenolic acids | 17 (17.3%) | 22 (4.6%) | 13 (19.1%) | 9 (5.5%) | 11 (18.3%) | 13 (5.3%) | 31 (12.7%) | 13 (6.4%) | 8 (13.3%) | 11 (12.4%) |
| Quinones | 3 (3.1%) | 4 (0.8%) | 0 (0.0%) | 1 (0.6%) | 0 (0.0%) | 3 (1.2%) | 2 (0.8%) | 2 (1.0%) | 1 (1.7%) | 0 (0.0%) |
| Steroids | 0 (0.0%) | 2 (0.4%) | 0 (0.0%) | 0 (0.0%) | 0 (0.0%) | 1 (0.4%) | 1 (0.0%) | 1 (0.5%) | 0 (0.0%) | 0 (0.0%) |
| Terpenoids | 8 (8.2%) | 101 (21.2%) | 9 (13.2%) | 56 (34.4%) | 13 (21.7%) | 60 (24.5%) | 23 (9.4%) | 53 (26.2%) | 6 (10.0%) | 15 (16.9%) |
| Tannins | 0 (0.0%) | 0 (0.0%) | 0 (0.0%) | 0 (0.0%) | 0 (0.0%) | 0 (0.0%) | 0 (0.0%) | 0 (0.0%) | 0 (0.0%) | 0 (0.0%) |
| **Root** | **RC1/RC0** | **RC2/RC0** | **RT0/RC0** | **RT1/RC1** | **RT2/RC2** | **RC1/RC0** | **RC2/RC0** | **RT0/RC0** | **RT1/RC1** | **RT2/RC2** |
| Alkaloids | 22 (13.1%) | 40 (12.3%) | 10 (6.0%) | 9 (10.7%) | 4 (3.4%) | 9 (7.4%) | 45 (8.8%) | 21 (8.0%) | 7 (5.8%) | 9 (5.0%) |
| Amino acids and derivatives | 10 (6.0%) | 25 (7.7%) | 10 (6.0%) | 2 (2.4%) | 4 (3.4%) | 7 (5.8%) | 53 (10.3%) | 22 (8.4%) | 6 (5.0%) | 3 (1.7%) |
| Flavonoids | 40 (23.8%) | 79 (24.2%) | 59 (35.1%) | 33 (39.3%) | 48 (40.7%) | 42 (34.7%) | 62 (12.1%) | 26 (10.0%) | 29 (24.2%) | 59 (32.6%) |
| Lignans and Coumarins | 3 (1.8%) | 4 (1.2%) | 8 (4.8%) | 4 (4.8%) | 3 (2.5%) | 5 (4.1%) | 34 (6.6%) | 7 (2.7%) | 3 (2.5%) | 4 (2.2%) |
| Lipids | 12 (7.1%) | 37 (11.3%) | 15 (8.9%) | 12 (14.3%) | 19 (16.1%) | 8 (6.6%) | 67 (13.1%) | 13 (5.0%) | 6 (5.0%) | 19 (10.5%) |
| Nucleotides and derivatives | 3 (1.8%) | 4 (1.2%) | 5 (3.0%) | 2 (2.4%) | 2 (1.7%) | 5(4.1%) | 38 (7.4%) | 25 (9.6%) | 7 (5.8%) | 4 (2.2%) |
| Organic acids | 2 (1.2%) | 11 (3.4%) | 4 (2.4%) | 3 (3.6%) | 2 (1.7%) | 6(5.0%) | 21 (4.1%) | 1 (0.4%) | 1 (0.8%) | 1 (0.6%) |
| Others | 27 (16.1%) | 47(14.4%) | 24 (14.3%) | 7 (8.3%) | 17 (14.4%) | 16 (13.2%) | 55 (10.7%) | 31 (11.9%) | 17 (14.2%) | 32 (17.7%) |
| Phenolic acids | 31 (18.5%) | 45 (13.8%) | 21 (12.5%) | 8 (9.5%) | 12 (10.2%) | 10 (8.3%) | 44 (8.6%) | 10 (3.8%) | 15 (12.5%) | 16 (8.8%) |
| Quinones | 3 (1.8%) | 4 (1.2%) | 0 (0.0%) | 0 (0.0%) | 0 (0.0%) | 0 (0.0%) | 9(1.8%) | 3 (1.1%) | 3(2.5%) | 3 (1.7%) |
| Steroids | 0 (0.0%) | 0 7(0.0%) | 0 (0.0%) | 0 (0.0%) | 1 (0.8%) | 1 (0.8%) | 5 (1.0%) | 0 (0.0%) | 0 (0.0%) | 0 (0.0%) |
| Terpenoids | 15 (8.9%) | 30 (9.2%) | 12 (7.1%) | 4 (4.8%) | 6 (5.1%) | 12 (9.9%) | 80 (15.6%) | 102 (39.1%) | 26 (21.7%) | 31 (17.1%) |
| Tannins | 0 (0.0%) | 0 (0.0%) | 0 (0.0%) | 0 (0.0%) | 0 (0.0%) | 0 (0.0%) | 0 (0.0%) | 0 (0.0%) | 0 (0.0%) | 0 (0.0%) |

**Table S3: Upregulated metabolites of DAMs in RT0/RC0 and ST0/SC0**

| Comparison group | Compounds | Class | VIP | P-value | Log2FC | Type |
| --- | --- | --- | --- | --- | --- | --- |
| RT0/RC0 | N5-(1-((Carboxymethyl)amino)-3-((3-(4-hydroxy-3,5-dimethoxyphenyl)allyl)thio)-1-oxopropan-2-yl)glutamine | Alkaloids | 1.28 | 0.044 | 1.67 | up |
| RT0/RC0 | 5-Methylnicotinic acid | Alkaloids | 1.35 | 0.000 | 1.07 | up |
| RT0/RC0 | N-Feruloyltyramine 4-glucoside | Alkaloids | 1.34 | 0.001 | 1.33 | up |
| RT0/RC0 | N-Feruloyloctopamine | Alkaloids | 1.25 | 0.044 | 1.49 | up |
| RT0/RC0 | Lappaconitine | Alkaloids | 1.35 | 0.005 | 3.65 | up |
| RT0/RC0 | Hypaphorine | Alkaloids | 1.34 | 0.017 | 3.23 | up |
| RT0/RC0 | Methylephedrine | Alkaloids | 1.34 | 0.034 | 2.99 | up |
| RT0/RC0 | Indolin-2-one | Alkaloids | 1.34 | 0.024 | 2.59 | up |
| RT0/RC0 | N,N-Dimethylformamide | Alkaloids | 1.35 | 0.003 | 4.90 | up |
| RT0/RC0 | N-Hydroxydecanoyl nornicotine glucoside | Alkaloids | 1.33 | 0.027 | 2.88 | up |
| RT0/RC0 | Methionine | Amino acids and derivatives | 1.34 | 0.003 | 1.07 | up |
| RT0/RC0 | N-Acetyl-L-leucine | Amino acids and derivatives | 1.10 | 0.020 | 1.36 | up |
| RT0/RC0 | Glu-Cys-Glu | Amino acids and derivatives | 1.27 | 0.007 | 1.42 | up |
| RT0/RC0 | L-Lysine-Butanoic Acid | Amino acids and derivatives | 1.34 | 0.024 | 4.03 | up |
| RT0/RC0 | N-Methyl-α-aminoisobutyric acid | Amino acids and derivatives | 1.34 | 0.024 | 4.03 | up |
| RT0/RC0 | Glu-Ser | Amino acids and derivatives | 1.34 | 0.024 | 4.03 | up |
| RT0/RC0 | Pro-Leu-Glu | Amino acids and derivatives | 1.34 | 0.013 | 2.95 | up |
| RT0/RC0 | Met-Abu-OH | Amino acids and derivatives | 1.31 | 0.001 | 1.02 | up |
| RT0/RC0 | PyroGlu-Ile | Amino acids and derivatives | 1.31 | 0.001 | 1.22 | up |
| RT0/RC0 | N6-(L-1,3-Dicarboxypropyl)-L-lysine | Amino acids and derivatives | 1.32 | 0.047 | 2.86 | up |
| RT0/RC0 | Isovitexin-2''-O-xyloside | Flavonoids | 1.35 | 0.003 | 7.29 | up |
| RT0/RC0 | Tricin-4'-O-(β-guaiacylglycerol)ether-5-O-(6''-malonyl)glucoside | Flavonoids | 1.35 | 0.017 | 5.82 | up |
| RT0/RC0 | 5-Desmethylsinensetin | Flavonoids | 1.35 | 0.022 | 5.25 | up |
| RT0/RC0 | Andrographidine D aglycone | Flavonoids | 1.34 | 0.050 | 5.22 | up |
| RT0/RC0 | Gardenin B | Flavonoids | 1.34 | 0.046 | 5.21 | up |
| RT0/RC0 | 5,7-Dihydroxy-6,3',4',5'-tetramethoxyflavone (Arteanoflavone) | Flavonoids | 1.35 | 0.011 | 5.06 | up |
| RT0/RC0 | Isovitexin-2''-O-rhamnoside (2''-O-alpha-L-Rhamnopyranosyl-isovitexin) | Flavonoids | 1.34 | 0.034 | 4.65 | up |
| RT0/RC0 | Vitexin 2''-O-beta-L-rhamnoside | Flavonoids | 1.34 | 0.034 | 4.65 | up |
| RT0/RC0 | Luteolin 7-O-(6''-malonylglucoside) | Flavonoids | 1.35 | 0.007 | 3.95 | up |
| RT0/RC0 | Prudomenin | Flavonoids | 1.35 | 0.005 | 3.88 | up |
| RT0/RC0 | Chiirirhamnin | Flavonoids | 1.35 | 0.000 | 3.79 | up |
| RT0/RC0 | 5-Methoxyluteolinidin | Flavonoids | 1.34 | 0.028 | 3.75 | up |
| RT0/RC0 | Vicenin-2 | Flavonoids | 1.33 | 0.048 | 3.65 | up |
| RT0/RC0 | Hispidulin-8-C-(2''-O-glucosyl)glucoside | Flavonoids | 1.34 | 0.025 | 3.47 | up |
| RT0/RC0 | 8-Methoxyluteolin-8-glucoside | Flavonoids | 1.34 | 0.030 | 3.25 | up |
| RT0/RC0 | kaempferol-3-caffeoyldiglucoside | Flavonoids | 1.34 | 0.014 | 3.11 | up |
| RT0/RC0 | 3,9-Dihydroxypterocarpan | Flavonoids | 1.35 | 0.010 | 3.04 | up |
| RT0/RC0 | Orientin-2''-O-rhamnoside | Flavonoids | 1.34 | 0.031 | 3.04 | up |
| RT0/RC0 | Kaempferol-3-O-(2''-(E)-feruloylgalactosyl-(1→4)-glucoside) | Flavonoids | 1.32 | 0.048 | 3.04 | up |
| RT0/RC0 | Tricin-5,7-O-diglucoside | Flavonoids | 1.34 | 0.019 | 2.99 | up |
| RT0/RC0 | Vicenin-3 | Flavonoids | 1.32 | 0.045 | 2.97 | up |
| RT0/RC0 | Nepetin-7-O-glucoside(Nepitrin) | Flavonoids | 1.33 | 0.037 | 2.90 | up |
| RT0/RC0 | Morin | Flavonoids | 1.34 | 0.016 | 2.89 | up |
| RT0/RC0 | Artocarpanone | Flavonoids | 1.34 | 0.016 | 2.89 | up |
| RT0/RC0 | 2',3,4,4',6'-Peptahydroxychalcone 4'-O-glucoside | Flavonoids | 1.33 | 0.027 | 2.81 | up |
| RT0/RC0 | Isorhamnetin 3-O-glucoside | Flavonoids | 1.34 | 0.023 | 2.81 | up |
| RT0/RC0 | Tricin-4'-O-syringylglyceryl ether-7-O-glucoside | Flavonoids | 1.33 | 0.034 | 2.80 | up |
| RT0/RC0 | Tricin-7-O-(6''-O-acetyl)glucoside | Flavonoids | 1.33 | 0.038 | 2.66 | up |
| RT0/RC0 | Tomentin | Flavonoids | 1.34 | 0.015 | 2.62 | up |
| RT0/RC0 | 5-Demethylnobiletin; 5-Hydroxy-6,7,8,3',4'-Pentamethoxyflavone | Flavonoids | 1.31 | 0.001 | 2.58 | up |
| RT0/RC0 | Kaempferol-3-O-robinobioside(Biorobin) | Flavonoids | 1.34 | 0.014 | 2.53 | up |
| RT0/RC0 | methyl 4,8-dihydroxy-6-methoxy-9-oxo-9H-xanthene-3-carboxylate | Flavonoids | 1.34 | 0.011 | 2.50 | up |
| RT0/RC0 | Gossypetin 3,7,8,3',4'-pentamethyl ether | Flavonoids | 1.34 | 0.000 | 2.49 | up |
| RT0/RC0 | Isovitexin-8-O-xyloside | Flavonoids | 1.26 | 0.046 | 2.48 | up |
| RT0/RC0 | Acacetin-6-C-glucoside | Flavonoids | 1.34 | 0.004 | 2.38 | up |
| RT0/RC0 | Tricin-4'-O-(guaiacylglycerol)ether-5-O-arabinoside | Flavonoids | 1.20 | 0.032 | 1.99 | up |
| RT0/RC0 | 6-C-Glucopyranosylpilloin | Flavonoids | 1.21 | 0.004 | 1.81 | up |
| RT0/RC0 | Swertisin 8-methyl ether | Flavonoids | 1.21 | 0.004 | 1.81 | up |
| RT0/RC0 | Isoorientin 7,3'-dimethyl ether | Flavonoids | 1.30 | 0.037 | 1.71 | up |
| RT0/RC0 | Gardenin C malonyl glucoside | Flavonoids | 1.27 | 0.022 | 1.59 | up |
| RT0/RC0 | 3,5,7-trihydroxy-dihydroflavone-6-C-glucoside-8-C-arabinoside | Flavonoids | 1.23 | 0.013 | 1.55 | up |
| RT0/RC0 | Vitexin-7-O-(6''-p-coumaroyl)glucoside | Flavonoids | 1.18 | 0.015 | 1.47 | up |
| RT0/RC0 | Kaempferol-3-O-(2''-p-Coumaroyl)galactoside | Flavonoids | 1.34 | 0.000 | 1.44 | up |
| RT0/RC0 | Poncirin | Flavonoids | 1.34 | 0.000 | 1.44 | up |
| RT0/RC0 | 5-Hydroxy-7,8,2',6'-Tetramethoxyflavone (Altisin) | Flavonoids | 1.26 | 0.003 | 1.39 | up |
| RT0/RC0 | Tribuloside | Flavonoids | 1.33 | 0.004 | 1.37 | up |
| RT0/RC0 | Didymin (Isosakuranetin-7-O-rutinoside) | Flavonoids | 1.33 | 0.004 | 1.37 | up |
| RT0/RC0 | Tricin-7-O-syringylalcohol | Flavonoids | 1.28 | 0.004 | 1.37 | up |
| RT0/RC0 | Tricin-4'-O-syringyl alcohol | Flavonoids | 1.28 | 0.004 | 1.37 | up |
| RT0/RC0 | 2-Hydroxy-2,3-dihydrogenistein | Flavonoids | 1.34 | 0.000 | 1.35 | up |
| RT0/RC0 | Narirutin | Flavonoids | 1.33 | 0.019 | 1.28 | up |
| RT0/RC0 | Naringin | Flavonoids | 1.34 | 0.008 | 1.24 | up |
| RT0/RC0 | 2-hydroxynaringenin | Flavonoids | 1.34 | 0.000 | 1.20 | up |
| RT0/RC0 | Tangeretin | Flavonoids | 1.27 | 0.014 | 1.14 | up |
| RT0/RC0 | 3',4',5',5,7-Pentamethoxyflavone | Flavonoids | 1.27 | 0.014 | 1.14 | up |
| RT0/RC0 | Hesperidin | Flavonoids | 1.32 | 0.006 | 1.11 | up |
| RT0/RC0 | Glabridin | Flavonoids | 1.29 | 0.003 | 1.05 | up |
| RT0/RC0 | Nobiletin | Flavonoids | 1.28 | 0.031 | 1.03 | up |
| RT0/RC0 | 3',4',5,5',6,7-Hexamethoxyflavone | Flavonoids | 1.27 | 0.026 | 1.01 | up |
| RT0/RC0 | [(1R,2S)-1-(1,3-benzodioxol-5-yl)-2-methyl-3-oxobutyl]4-hydroxy-3-methoxybenzoate | Lignans and Coumarins | 1.29 | 0.003 | 1.09 | up |
| RT0/RC0 | Decursinol | Lignans and Coumarins | 1.24 | 0.031 | 1.13 | up |
| RT0/RC0 | 6-Hydroxymethylherniarin | Lignans and Coumarins | 1.35 | 0.007 | 3.23 | up |
| RT0/RC0 | 3,4-Divanillyltetrahydrofuran | Lignans and Coumarins | 1.16 | 0.019 | 1.25 | up |
| RT0/RC0 | 8-Hydroxycoumarin | Lignans and Coumarins | 1.34 | 0.016 | 3.61 | up |
| RT0/RC0 | Divanillyltetrahydrofuran | Lignans and Coumarins | 1.35 | 0.002 | 4.85 | up |
| RT0/RC0 | Aesculetin-6-O-β-D-apiofuranosyl-(1→6)-O-β-glucopyranoside | Lignans and Coumarins | 1.13 | 0.031 | 1.05 | up |
| RT0/RC0 | (1R,2S)-2-[4-[(3R,3aR,6R,6aS)-3-(4-Hydroxy-3,5-dimethoxyphenyl)-1,3,3a,4,6,6a-hexahydrofuro[3,4-c]furan-6-yl]-2,6-dimethoxyphenoxy]-1-(4-hydroxy-3,5-dimethoxyphenyl)propane-1,3-diol | Lignans and Coumarins | 1.34 | 0.000 | 1.05 | up |
| RT0/RC0 | 2-Aminotetradecane-1,11,13-triol | Lipids | 1.23 | 0.016 | 1.44 | up |
| RT0/RC0 | 2-Aminohexadecane-1,5,15-triol | Lipids | 1.28 | 0.002 | 1.36 | up |
| RT0/RC0 | 2-Aminotetradecane-1,5,13-triol | Lipids | 1.21 | 0.036 | 1.35 | up |
| RT0/RC0 | 2-Aminohexadecane-1,5,6-triol | Lipids | 1.31 | 0.001 | 1.04 | up |
| RT0/RC0 | LysoPC 16:2 | Lipids | 1.25 | 0.010 | 1.08 | up |
| RT0/RC0 | 1-(9Z-Octadecenoyl)-2-(9-oxo-nonanoyl)-sn-glycero-3-phosphocholine | Lipids | 1.34 | 0.022 | 2.67 | up |
| RT0/RC0 | LysoPC 19:2 | Lipids | 1.31 | 0.018 | 1.04 | up |
| RT0/RC0 | [(2R)-2-(8-carboxyoctanoyloxy)-3-hexadecanoyloxypropyl] 2-(trimethylazaniumyl)ethyl phosphate | Lipids | 1.33 | 0.009 | 1.08 | up |
| RT0/RC0 | 2-(2,3-dihydroxypropoxy)-3-(((2-(dimethylamino)ethoxy)(hydroxy)phosphoryl)oxy)propan-2-yl (Z)-14-Octadecenoic Acid | Lipids | 1.34 | 0.000 | 1.15 | up |
| RT0/RC0 | (R)-2-Hydroxystearate | Lipids | 1.33 | 0.004 | 1.32 | up |
| RT0/RC0 | Sphinganine 1-phosphate | Lipids | 1.34 | 0.007 | 1.70 | up |
| RT0/RC0 | DL-2-hydroxystearic acid | Lipids | 1.35 | 0.001 | 1.24 | up |
| RT0/RC0 | 19,20-DiHDPA | Lipids | 1.35 | 0.002 | 8.62 | up |
| RT0/RC0 | 12-Hydroxyoctadecanoic acid | Lipids | 1.34 | 0.011 | 2.49 | up |
| RT0/RC0 | PA(18:2/0:0) | Lipids | 1.35 | 0.017 | 8.24 | up |
| RT0/RC0 | AICA ribonucleotide | Nucleotides and derivatives | 1.31 | 0.001 | 1.13 | up |
| RT0/RC0 | ATP | Nucleotides and derivatives | 1.32 | 0.041 | 1.98 | up |
| RT0/RC0 | FAD | Nucleotides and derivatives | 1.31 | 0.013 | 1.17 | up |
| RT0/RC0 | 2-Deoxy-D-ribose 5-phosphate | Nucleotides and derivatives | 1.35 | 0.006 | 2.44 | up |
| RT0/RC0 | 2'-Deoxyuridine 5'-monophosphate | Nucleotides and derivatives | 1.28 | 0.029 | 1.12 | up |
| RT0/RC0 | Dimethylallyl diphosphate | Organic acids | 1.13 | 0.049 | 1.04 | up |
| RT0/RC0 | Phosphoenolpyruvate | Organic acids | 1.25 | 0.005 | 1.20 | up |
| RT0/RC0 | Litchioside C | Organic acids | 1.34 | 0.025 | 3.19 | up |
| RT0/RC0 | Citric Acid glucuronide | Organic acids | 1.28 | 0.029 | 1.12 | up |
| RT0/RC0 | 4-hydroxyphenyl acrylaldehyde | Others | 1.27 | 0.007 | 1.05 | up |
| RT0/RC0 | Riboflavin | Others | 1.30 | 0.011 | 1.65 | up |
| RT0/RC0 | 3-Hydroxytropolone | Others | 1.32 | 0.006 | 1.20 | up |
| RT0/RC0 | Aplidiasphingosine | Others | 1.30 | 0.029 | 1.17 | up |
| RT0/RC0 | 2,4,6-Trihydroxybenzophenone | Others | 1.34 | 0.031 | 4.35 | up |
| RT0/RC0 | Sporormielloside | Others | 1.34 | 0.022 | 2.92 | up |
| RT0/RC0 | Butyl beta-D-glucoside | Others | 1.35 | 0.010 | 3.79 | up |
| RT0/RC0 | 1-(3-Methoxy-4-hydroxyphenyl)-7-(3,5-dimethoxy-4-hydroxyphenyl)-4,6-heptadiene-3-one | Others | 1.34 | 0.034 | 5.78 | up |
| RT0/RC0 | 4-Ethynylbenzaldehyde | Others | 1.34 | 0.015 | 3.17 | up |
| RT0/RC0 | Thiamine | Others | 1.35 | 0.020 | 7.37 | up |
| RT0/RC0 | 2,4-Dinitrophenol | Others | 1.31 | 0.000 | 1.69 | up |
| RT0/RC0 | Phenyl acetate | Others | 1.30 | 0.040 | 1.38 | up |
| RT0/RC0 | D-Threonate | Others | 1.27 | 0.004 | 1.15 | up |
| RT0/RC0 | 3,4-dihydroxybenzaldehyde-xylose-glucoside | Others | 1.21 | 0.028 | 1.08 | up |
| RT0/RC0 | 4,6-Dimethoxy-5-methyl-2H-Pyran-2-one | Others | 1.15 | 0.022 | 1.19 | up |
| RT0/RC0 | Coniferin | Others | 1.35 | 0.001 | 3.55 | up |
| RT0/RC0 | 2-Hydroxy-7-carboxy-1-methyl-5-ethenyl-9,10-dihydrophenanthrene | Others | 1.35 | 0.002 | 5.50 | up |
| RT0/RC0 | 2-Hydroxy-8-carboxy-1-methyl-5-ethenyl-9,10-dihydrophenanthrene | Others | 1.35 | 0.002 | 5.50 | up |
| RT0/RC0 | 4-Hydroxyphenylethanol | Others | 1.34 | 0.024 | 3.59 | up |
| RT0/RC0 | Resveratroloside | Others | 1.33 | 0.048 | 3.10 | up |
| RT0/RC0 | 3'-Norspongiolactone | Others | 1.35 | 0.007 | 7.49 | up |
| RT0/RC0 | 4-methylbenzenesulfonic (11E,14E)-octadeca-11,14,17-trienoic anhydride | Others | 1.35 | 0.002 | 8.67 | up |
| RT0/RC0 | Dambonitol | Others | 1.35 | 0.004 | 4.14 | up |
| RT0/RC0 | 3-Nitrophenol | Others | 1.33 | 0.039 | 2.92 | up |
| RT0/RC0 | 4-O-Feruloyl Aminogalactitol | Phenolic acids | 1.33 | 0.039 | 3.36 | up |
| RT0/RC0 | 4-O-Galactosyl-D-Xylose | Phenolic acids | 1.30 | 0.001 | 1.23 | up |
| RT0/RC0 | Protocatechuic acid glucosyl xyloside | Phenolic acids | 1.31 | 0.004 | 1.54 | up |
| RT0/RC0 | 5-{[2-O-(beta-d-apiofuranosyl)-beta-d-glucopyranosyl]oxy}-2-hydroxybenzoic acid | Phenolic acids | 1.31 | 0.014 | 1.69 | up |
| RT0/RC0 | 3-O-(3-Methoxy-4-hydroxybenzoyl)-4-O-caffeoylquinic acid | Phenolic acids | 1.31 | 0.003 | 1.16 | up |
| RT0/RC0 | Feruloylcaffeoyltartaric acid | Phenolic acids | 1.30 | 0.016 | 1.03 | up |
| RT0/RC0 | Apiosylglucosyl 4-hydroxybenzoate | Phenolic acids | 1.21 | 0.028 | 1.08 | up |
| RT0/RC0 | 2-(3,4-dihydroxyphenethoxy)6-mustardyl-O-glucosyl-D-xylose | Phenolic acids | 1.32 | 0.048 | 2.88 | up |
| RT0/RC0 | Bilobol | Phenolic acids | 1.34 | 0.001 | 8.38 | up |
| RT0/RC0 | Furanofructosyl-α-D-(6-mustard acyl)glucoside | Phenolic acids | 1.34 | 0.015 | 2.95 | up |
| RT0/RC0 | Benzoyl-Beta-D-Glucoside | Phenolic acids | 1.30 | 0.025 | 1.28 | up |
| RT0/RC0 | 6-O-Glucosyl-caffeoylbenzoic acid | Phenolic acids | 1.35 | 0.014 | 3.62 | up |
| RT0/RC0 | 4-O-(6'-O-Glucosylcaffeoylglucosyl)-4-hydroxybenzyl alcohol | Phenolic acids | 1.35 | 0.008 | 3.00 | up |
| RT0/RC0 | 3-Spinoyl-6'-acetyl-sucrose | Phenolic acids | 1.34 | 0.023 | 2.70 | up |
| RT0/RC0 | Psoralenoside | Phenolic acids | 1.32 | 0.049 | 3.02 | up |
| RT0/RC0 | 3-O-Feruloyl Methylglucaric Acid | Phenolic acids | 1.32 | 0.044 | 2.94 | up |
| RT0/RC0 | Isosalicin | Phenolic acids | 1.33 | 0.038 | 2.94 | up |
| RT0/RC0 | 3,4-dihydroxy-β-phenylethoxy-O-α-L-rhamnopyranosyl(1→3)-6-O-caffeoyl(cis)-β-D-glucopyranoside | Phenolic acids | 1.34 | 0.020 | 2.72 | up |
| RT0/RC0 | 5-O-p-Coumaroylgalactaric acid | Phenolic acids | 1.32 | 0.048 | 3.04 | up |
| RT0/RC0 | 1-O-Feruloyl Ranunculin | Phenolic acids | 1.34 | 0.021 | 3.27 | up |
| RT0/RC0 | 1-O-Caffeoyl-β-D-xylose | Phenolic acids | 1.34 | 0.010 | 2.58 | up |
| RT0/RC0 | 2-Hydroxy-Pimara-5,15-dien-19-oic acid | Terpenoids | 1.33 | 0.047 | 3.73 | up |
| RT0/RC0 | Norflickinflimiod F | Terpenoids | 1.35 | 0.015 | 4.01 | up |
| RT0/RC0 | Grasshopper ketone | Terpenoids | 1.32 | 0.043 | 3.00 | up |
| RT0/RC0 | (6R,9R)-3-Oxo-α-ionol-β-D-malonyl-glucoside | Terpenoids | 1.35 | 0.010 | 4.45 | up |
| RT0/RC0 | Farnesyl acetone | Terpenoids | 1.35 | 0.008 | 6.98 | up |
| RT0/RC0 | Flamvelutpenoid C | Terpenoids | 1.14 | 0.034 | 1.00 | up |
| RT0/RC0 | Geranyl 3-O-xylopyranosyl-glucopyranoside | Terpenoids | 1.32 | 0.015 | 1.17 | up |
| RT0/RC0 | (1S,4aS,7aR)-7-(3-hydroxybutyl)-4-((((2R,3R,4S,5S,6R)-3,4,5-trihydroxy-6-(hydroxymethyl)tetrahydro-2H-pyran-2-yl)oxy)methyl)-1,4a,5,6,7,7a-hexahydrocyclopenta[c]pyran-1-yl acetate | Terpenoids | 1.34 | 0.019 | 2.88 | up |
| RT0/RC0 | 6-O-Sinapoylajugol | Terpenoids | 1.33 | 0.038 | 2.79 | up |
| RT0/RC0 | Asperulosidic acid | Terpenoids | 1.33 | 0.046 | 4.23 | up |
| RT0/RC0 | 3,4-Open loop-lupine-4(23),20(29)-diene-24-hydroxy-3-carboxylic acid | Terpenoids | 1.34 | 0.023 | 3.60 | up |
| RT0/RC0 | 3-Acetoxy-9,13-epoxy-16-hydroxy-labda-15,16-olide | Terpenoids | 1.34 | 0.026 | 3.68 | up |
| ST0/SC0 | 4-[2-(1-methylethyl)aminoethyl]phenol | Alkaloids | 1.53 | 0.005 | 1.28 | up |
| ST0/SC0 | L-Carnitine | Alkaloids | 1.53 | 0.005 | 2.74 | up |
| ST0/SC0 | 2,3-dihydro-6-hydroxyltryptophan N-Glucoside | Alkaloids | 1.49 | 0.005 | 1.23 | up |
| ST0/SC0 | Lappaconitine | Alkaloids | 1.53 | 0.002 | 2.38 | up |
| ST0/SC0 | 2-Amino-4-dihydroxy octadecyl galactoside | Alkaloids | 1.53 | 0.008 | 2.97 | up |
| ST0/SC0 | 2-Glucosyloxy-4-hydroxybenzeneacetonitrile | Alkaloids | 1.53 | 0.001 | 2.98 | up |
| ST0/SC0 | N-(E-4-coumaroyl)-aspartate | Amino acids and derivatives | 1.52 | 0.022 | 3.05 | up |
| ST0/SC0 | (4-methyl-2-(tyrosyloxy)pentanoyl)glutamine | Amino acids and derivatives | 1.52 | 0.026 | 2.77 | up |
| ST0/SC0 | L-2-Aminoadipate 6-semialdehyde | Amino acids and derivatives | 1.54 | 0.000 | 5.23 | up |
| ST0/SC0 | Andrographidine D aglycone | Flavonoids | 1.53 | 0.004 | 5.44 | up |
| ST0/SC0 | Gardenin B | Flavonoids | 1.53 | 0.001 | 5.11 | up |
| ST0/SC0 | 5-Hydroxyauranetin | Flavonoids | 1.53 | 0.002 | 4.48 | up |
| ST0/SC0 | 5-Hydroxy-4',6,7-trimethoxyflavone (Salvigenin) | Flavonoids | 1.53 | 0.002 | 4.35 | up |
| ST0/SC0 | Gossypetin 3,7,8,3',4'-pentamethyl ether | Flavonoids | 1.53 | 0.002 | 4.03 | up |
| ST0/SC0 | 5,7-Dihydroxy-6,3',4',5'-tetramethoxyflavone (Arteanoflavone) | Flavonoids | 1.53 | 0.000 | 3.92 | up |
| ST0/SC0 | 5-Demethylnobiletin; 5-Hydroxy-6,7,8,3',4'-Pentamethoxyflavone | Flavonoids | 1.53 | 0.002 | 3.91 | up |
| ST0/SC0 | Quercetin 3,5,7,3,4-pentamethyl ether | Flavonoids | 1.53 | 0.019 | 3.80 | up |
| ST0/SC0 | 5-Hydroxy-3,7,3',4'-tetramethoxyflavone (Retusin) | Flavonoids | 1.51 | 0.002 | 3.76 | up |
| ST0/SC0 | 5-Hydroxy-7,8,2',6'-Tetramethoxyflavone (Altisin) | Flavonoids | 1.53 | 0.007 | 3.59 | up |
| ST0/SC0 | 5-Desmethylsinensetin | Flavonoids | 1.51 | 0.000 | 3.46 | up |
| ST0/SC0 | Petunidin-3-O-arabinoside | Flavonoids | 1.51 | 0.045 | 2.85 | up |
| ST0/SC0 | 2,6,7,4'-Tetrahydroxyisoflavanone | Flavonoids | 1.52 | 0.031 | 2.72 | up |
| ST0/SC0 | Oxygenated Xanthohumol | Flavonoids | 1.52 | 0.021 | 2.68 | up |
| ST0/SC0 | Dihydromyricetin | Flavonoids | 1.45 | 0.011 | 2.12 | up |
| ST0/SC0 | Tangeretin | Flavonoids | 1.50 | 0.000 | 2.07 | up |
| ST0/SC0 | 3',4',5',5,7-Pentamethoxyflavone | Flavonoids | 1.50 | 0.000 | 2.07 | up |
| ST0/SC0 | Viscumneoside IV(Rhamnazin-3-O-(6''-hydroxymethylglutaryl)glucoside) | Flavonoids | 1.44 | 0.012 | 1.41 | up |
| ST0/SC0 | Syringetin-7-O-glucoside | Flavonoids | 1.52 | 0.000 | 1.29 | up |
| ST0/SC0 | 5,7,8,3',4',5'-hexamethoxyflavone | Flavonoids | 1.39 | 0.028 | 1.20 | up |
| ST0/SC0 | 3',4',5,5',6,7-Hexamethoxyflavone | Flavonoids | 1.39 | 0.029 | 1.18 | up |
| ST0/SC0 | Nobiletin | Flavonoids | 1.43 | 0.034 | 1.15 | up |
| ST0/SC0 | [(1R,2S)-1-(1,3-benzodioxol-5-yl)-2-methyl-3-oxobutyl]4-hydroxy-3-methoxybenzoate | Lignans and Coumarins | 1.51 | 0.001 | 1.82 | up |
| ST0/SC0 | Murraol | Lignans and Coumarins | 1.52 | 0.040 | 3.77 | up |
| ST0/SC0 | LysoPC 20:5 | Lipids | 1.53 | 0.010 | 3.36 | up |
| ST0/SC0 | 1-Oleoyl-Sn-Glycerol | Lipids | 1.52 | 0.021 | 2.59 | up |
| ST0/SC0 | Leukotriene F4 | Lipids | 1.27 | 0.019 | 1.12 | up |
| ST0/SC0 | 19,20-DiHDPA | Lipids | 1.49 | 0.003 | 2.07 | up |
| ST0/SC0 | Triethyl citrate | Organic acids | 1.47 | 0.008 | 1.14 | up |
| ST0/SC0 | 3-(2-hydroxyethyl)-5,7-dimethoxy-4-methyl-2H-1-benzopyran-2-one | Others | 1.53 | 0.009 | 2.57 | up |
| ST0/SC0 | 5,8-dihydroxy-1-hydroxymethylnaphtho[2,3-c]furan-4,9-dione | Others | 1.52 | 0.039 | 3.39 | up |
| ST0/SC0 | 3,4-dihydroxybenzaldehyde-xylose-glucoside | Others | 1.15 | 0.040 | 1.30 | up |
| ST0/SC0 | 4-Hydroxy-3,5-dimethoxybenzyl alcohol | Others | 1.54 | 0.000 | 2.36 | up |
| ST0/SC0 | (3E,5E,8Z,11Z)-10,13,15-trimethylheptadeca-3,5,8,11-tetraenoic acid | Others | 1.53 | 0.011 | 2.59 | up |
| ST0/SC0 | Benzyl alcohol xylopyranosyl-(1-6)-glucopyranoside | Others | 1.53 | 0.025 | 3.87 | up |
| ST0/SC0 | 4-methylbenzenesulfonic octadec-17-enoic anhydride | Others | 1.53 | 0.009 | 2.56 | up |
| ST0/SC0 | [(2S,3R,4S,5S,6R)-3,4,5-trihydroxy-6-(hydroxymethyl)oxan-2-yl](2E,4E)-5-[(1R,3S,5S,8S)-3,8-dihydroxy-1,5-dimethyl-6-oxabicyclo[3.2.1]octan-8-yl]-3-methylpenta-2,4-dienoate | Others | 1.54 | 0.000 | 2.41 | up |
| ST0/SC0 | 5-hydroxy-l,7-bis(4-hydroxyphenyl)hept-1-en-3-one | Phenolic acids | 1.52 | 0.030 | 3.15 | up |
| ST0/SC0 | 4-O-Galactosyl-D-Xylose | Phenolic acids | 1.53 | 0.001 | 1.74 | up |
| ST0/SC0 | 3-O-p-Coumaroylquinic acid | Phenolic acids | 1.51 | 0.000 | 1.12 | up |
| ST0/SC0 | Protocatechuic acid glucosyl xyloside | Phenolic acids | 1.51 | 0.004 | 1.92 | up |
| ST0/SC0 | 5-{[2-O-(beta-d-apiofuranosyl)-beta-d-glucopyranosyl]oxy}-2-hydroxybenzoic acid | Phenolic acids | 1.43 | 0.004 | 1.27 | up |
| ST0/SC0 | 1,3-O-Di-p-Coumaroylglycerol | Phenolic acids | 1.53 | 0.002 | 1.22 | up |
| ST0/SC0 | Apiosylglucosyl 4-hydroxybenzoate | Phenolic acids | 1.15 | 0.040 | 1.30 | up |
| ST0/SC0 | Plantamajoside | Phenolic acids | 1.48 | 0.002 | 1.81 | up |
| ST0/SC0 | 1-O-Galloyl-4-O-p-Coumaroyl-β-D-glucose | Phenolic acids | 1.53 | 0.002 | 2.78 | up |
| ST0/SC0 | Vnilloylcaffeoyltartaric acid | Phenolic acids | 1.53 | 0.025 | 4.14 | up |
| ST0/SC0 | 2-(3,4-dihydroxyphenylmethyl)-6-feruloyl-O-glucosyl-D-xylose | Phenolic acids | 1.52 | 0.035 | 3.76 | up |
| ST0/SC0 | 4-Hydroxybenzoic acid 4-(6-O-sulfo)glucopyranoside | Phenolic acids | 1.53 | 0.009 | 2.57 | up |
| ST0/SC0 | 1-O-Feruloyl-3-O-caffeoylglycerol | Phenolic acids | 1.53 | 0.031 | 4.23 | up |
| ST0/SC0 | Rubellacrn E | Terpenoids | 1.52 | 0.019 | 3.30 | up |
| ST0/SC0 | 8(14),15-Isopimaradiene-1,9-diol | Terpenoids | 1.53 | 0.003 | 2.45 | up |
| ST0/SC0 | Abietal | Terpenoids | 1.50 | 0.047 | 2.89 | up |
| ST0/SC0 | methyl 1,4a-dimethyl-6-methylidene-5-(3-oxobutyl)-3,4,5,7,8,8a-hexahydro-2H-naphthalene-1-carboxylate | Terpenoids | 1.51 | 0.031 | 2.81 | up |
| ST0/SC0 | 12,13-Dihydroxy-7-Oxo-8(14)-Abieten-18-Oic Acid | Terpenoids | 1.50 | 0.047 | 2.89 | up |
| ST0/SC0 | 3-hydroxy-1-(hydroxymethyl)-1,7-dimethyl-7-vinyl-2,3,4,4a,4b,5,6,7,10,10a-decahydrophenanthren-9(1H)-one | Terpenoids | 1.53 | 0.009 | 2.57 | up |
| ST0/SC0 | 13-O-Feruloylplumieride | Terpenoids | 1.53 | 0.014 | 4.26 | up |
| ST0/SC0 | Communic Acid | Terpenoids | 1.51 | 0.002 | 2.53 | up |
| ST0/SC0 | Hydroxygeraniol apiosylglucoside | Terpenoids | 1.53 | 0.005 | 3.70 | up |

**Table S4: Upregulated metabolites of DAMs in RT1/RC1 and RT2/RC2**

| Comparison group | Compounds | Class | VIP | P-value | Log2FC | Type |
| --- | --- | --- | --- | --- | --- | --- |
| RT1/RC1 | Quinidine | Alkaloids | 1.52 | 0.006 | 4.69 | up |
| RT1/RC1 | N1-Methyl-2-pyridone-5-carboxamide | Alkaloids | 1.51 | 0.039 | 4.48 | up |
| RT1/RC1 | tryptamineisovalerate | Alkaloids | 1.52 | 0.006 | 4.18 | up |
| RT1/RC1 | Fuzitine | Alkaloids | 1.51 | 0.039 | 3.62 | up |
| RT1/RC1 | Salicylamide | Alkaloids | 1.52 | 0.008 | 3.32 | up |
| RT1/RC1 | 4-Hydroxyaniline | Alkaloids | 1.51 | 0.031 | 3.15 | up |
| RT1/RC1 | Creatinine | Alkaloids | 1.52 | 0.003 | 2.86 | up |
| RT1/RC1 | N-Hydroxydecanoyl nornicotine glucoside | Alkaloids | 1.51 | 0.021 | 2.72 | up |
| RT1/RC1 | (2R,3S,4S,5R,6S)-2-(hydroxymethyl)-6-[2-[(4R)-4-(hydroxymethyl)-4,5-dihydro-1,3-oxazol-2-yl]phenoxy]oxane-3,4,5-triol | Alkaloids | 1.52 | 0.000 | 2.52 | up |
| RT1/RC1 | Pro-Val-Leu | Amino acids and derivatives | 1.52 | 0.006 | 3.41 | up |
| RT1/RC1 | Met-Lys-Gly | Amino acids and derivatives | 1.52 | 0.012 | 2.59 | up |
| RT1/RC1 | Chrysoeriol feruloyl glucosyl glucoside | Flavonoids | 1.52 | 0.001 | 5.71 | up |
| RT1/RC1 | Tricin-7-O-(6'-O-glucoside)glucuronicacid | Flavonoids | 1.52 | 0.022 | 4.95 | up |
| RT1/RC1 | Tricin-4'-O-(guaiacylglycerol)ether-5-O-arabinoside | Flavonoids | 1.52 | 0.029 | 4.92 | up |
| RT1/RC1 | Luteolin-8-C-arabinoside | Flavonoids | 1.52 | 0.028 | 4.86 | up |
| RT1/RC1 | Isovitexin-2''-O-xyloside | Flavonoids | 1.52 | 0.001 | 4.42 | up |
| RT1/RC1 | Tricin-7-O-(2''-O-glucosyl)glucoside | Flavonoids | 1.52 | 0.013 | 4.42 | up |
| RT1/RC1 | Apigenin-8-C-glucoside-7-O-Sophoroside | Flavonoids | 1.52 | 0.018 | 4.09 | up |
| RT1/RC1 | Dihydrokaempferide | Flavonoids | 1.51 | 0.035 | 3.79 | up |
| RT1/RC1 | Tricin-4'-O-(syringyl alcohol)ether-7-O-glucoside | Flavonoids | 1.52 | 0.005 | 3.54 | up |
| RT1/RC1 | Kaempferol-3-O-(2''-(E)-feruloylgalactosyl-(1→4)-glucoside) | Flavonoids | 1.52 | 0.008 | 3.46 | up |
| RT1/RC1 | 3'-Methoxydaidzin | Flavonoids | 1.52 | 0.004 | 3.20 | up |
| RT1/RC1 | 5-Hydroxy-4',6,7-trimethoxyflavone (Salvigenin) | Flavonoids | 1.51 | 0.022 | 3.08 | up |
| RT1/RC1 | Chrysin-5-O-glucoside (Toringin) | Flavonoids | 1.50 | 0.045 | 3.05 | up |
| RT1/RC1 | Tricin-4'-O-(β-guaiacylglycerol)ether-7-O-(6''-malonyl)glucoside | Flavonoids | 1.49 | 0.049 | 2.95 | up |
| RT1/RC1 | Tricin-7-O-syringylalcoholether-4'-O-glucoside | Flavonoids | 1.50 | 0.031 | 2.86 | up |
| RT1/RC1 | Quercetin-3',4'-dimethyl ether | Flavonoids | 1.51 | 0.017 | 2.73 | up |
| RT1/RC1 | Spinosin | Flavonoids | 1.51 | 0.021 | 2.71 | up |
| RT1/RC1 | Nepetin-7-O-alloside | Flavonoids | 1.51 | 0.019 | 2.69 | up |
| RT1/RC1 | Tricin-4'-O-((6'''-O-sinacyl)-6''-O-glucoside)glucuronide | Flavonoids | 1.51 | 0.017 | 2.68 | up |
| RT1/RC1 | Psiadiarabicin | Flavonoids | 1.51 | 0.017 | 2.58 | up |
| RT1/RC1 | Tricin-4'-O-(syringyl alcohol)ether-5-O-glucoside | Flavonoids | 1.51 | 0.030 | 2.58 | up |
| RT1/RC1 | 3,9-dihydroeucomnalin glucoside | Flavonoids | 1.52 | 0.010 | 2.49 | up |
| RT1/RC1 | Isovitexin 2''-O-beta-D-glucoside | Flavonoids | 1.52 | 0.000 | 2.33 | up |
| RT1/RC1 | 5,6,7-Trihydroxyflavone 7-(6''-malonylglucoside) | Flavonoids | 1.45 | 0.008 | 1.68 | up |
| RT1/RC1 | Apigenin 7-(6''-malonylglucoside) | Flavonoids | 1.48 | 0.021 | 1.46 | up |
| RT1/RC1 | Phellodensin E | Flavonoids | 1.48 | 0.021 | 1.46 | up |
| RT1/RC1 | Monohydroxy-trimethoxyflavone-O-(6''-malonyl)glucoside | Flavonoids | 1.44 | 0.044 | 1.40 | up |
| RT1/RC1 | Tricin-5-O-Glucoside | Flavonoids | 1.28 | 0.013 | 1.34 | up |
| RT1/RC1 | Apigenin 5-(6''-malonylglucoside) | Flavonoids | 1.46 | 0.014 | 1.28 | up |
| RT1/RC1 | Tricin-7-O-(2''-feruloyl)glucoside | Flavonoids | 1.36 | 0.043 | 1.14 | up |
| RT1/RC1 | 3,9-Dihydroxypterocarpan | Flavonoids | 1.46 | 0.001 | 1.13 | up |
| RT1/RC1 | Acacetin-7-O-neohesperidoside | Flavonoids | 1.32 | 0.040 | 1.04 | up |
| RT1/RC1 | 4,4'-Dihydroxy-2,6-dimethoxydihydrochalcone | Flavonoids | 1.50 | 0.000 | 1.04 | up |
| RT1/RC1 | Fraxidinglucoside | Lignans and Coumarins | 1.52 | 0.018 | 4.06 | up |
| RT1/RC1 | Cichoriin | Lignans and Coumarins | 1.52 | 0.019 | 3.57 | up |
| RT1/RC1 | Bursehernin | Lignans and Coumarins | 1.51 | 0.018 | 2.72 | up |
| RT1/RC1 | dihydrodehydrodiconiferyl alcohol-9-O-β-D-xylopyranoside | Lignans and Coumarins | 1.28 | 0.013 | 1.34 | up |
| RT1/RC1 | PA(18:2/0:0) | Lipids | 1.52 | 0.022 | 8.20 | up |
| RT1/RC1 | 13-Hydroperoxy-9Z,11E-octadecadienoic acid | Lipids | 1.52 | 0.002 | 3.29 | up |
| RT1/RC1 | 2-Aminooctadecane-1,16,18,18-tetraol | Lipids | 1.50 | 0.003 | 1.50 | up |
| RT1/RC1 | 2-Aminooctadecane-1,5,7,17-tetraol | Lipids | 1.50 | 0.003 | 1.47 | up |
| RT1/RC1 | C16 phytosphingosine | Lipids | 1.52 | 0.001 | 1.46 | up |
| RT1/RC1 | 2-Aminohexadecane-1,5,6-triol | Lipids | 1.51 | 0.000 | 1.37 | up |
| RT1/RC1 | 2-Aminohexadecane-1,16,16-triol | Lipids | 1.52 | 0.000 | 1.36 | up |
| RT1/RC1 | LysoPC 19:2(2n isomer) | Lipids | 1.36 | 0.036 | 1.28 | up |
| RT1/RC1 | 20-Carboxyarachidonic Acid | Lipids | 1.51 | 0.001 | 1.27 | up |
| RT1/RC1 | [(2R)-2-(8-carboxyoctanoyloxy)-3-hexadecanoyloxypropyl] 2-(trimethylazaniumyl)ethyl phosphate | Lipids | 1.47 | 0.013 | 1.06 | up |
| RT1/RC1 | Sphinganine 1-phosphate | Lipids | 1.30 | 0.018 | 1.04 | up |
| RT1/RC1 | 2-Aminohexadecane-1,5,15-triol | Lipids | 1.28 | 0.030 | 1.02 | up |
| RT1/RC1 | dGTP | Nucleotides and derivatives | 1.45 | 0.025 | 1.06 | up |
| RT1/RC1 | ATP | Nucleotides and derivatives | 1.43 | 0.011 | 1.01 | up |
| RT1/RC1 | Mevalonic Acid Glucoside | Organic acids | 1.52 | 0.016 | 3.99 | up |
| RT1/RC1 | Dimethylglyceric Acid Glucoside | Organic acids | 1.50 | 0.043 | 2.67 | up |
| RT1/RC1 | Triethyl citrate | Organic acids | 1.37 | 0.006 | 1.19 | up |
| RT1/RC1 | 3-ethyl-7-hydroxy-5,6-dimethoxyphthalide | Others | 1.51 | 0.038 | 3.38 | up |
| RT1/RC1 | Aquilegiolide | Others | 1.50 | 0.042 | 2.94 | up |
| RT1/RC1 | 3,4-Dihydroxyphenethyl alcohol-8-O-[4-O-caffeoyl-β-D-apinosyl(1→3)-β-D-glucosyl(1→6)]-β-D-glucoside | Others | 1.51 | 0.021 | 2.71 | up |
| RT1/RC1 | Methyl 5-phenyl-4-pentynoate | Others | 1.52 | 0.002 | 2.54 | up |
| RT1/RC1 | 3-(1-hydroxyethyl)-4-methylpentane-1,4-diol O-Glucoside | Others | 1.45 | 0.002 | 1.13 | up |
| RT1/RC1 | 2,2-dimethylchromene-6-carboxylic acid | Others | 1.40 | 0.041 | 1.03 | up |
| RT1/RC1 | Pyrocatechol monoglucoside | Others | 1.48 | 0.019 | 1.02 | up |
| RT1/RC1 | 2-O-Caffeoylmalic acid | Phenolic acids | 1.51 | 0.046 | 3.68 | up |
| RT1/RC1 | Mono(2-ethyl-5-hydroxyhexyl) phthalate | Phenolic acids | 1.51 | 0.024 | 3.67 | up |
| RT1/RC1 | Sinapate | Phenolic acids | 1.51 | 0.028 | 3.55 | up |
| RT1/RC1 | 3-Spinoyl-6'-acetyl-sucrose | Phenolic acids | 1.52 | 0.013 | 2.96 | up |
| RT1/RC1 | Protocatechuic Acid Methyl Ester | Phenolic acids | 1.51 | 0.019 | 2.69 | up |
| RT1/RC1 | 4-Hydroxybenzoyl acetyl glucoside | Phenolic acids | 1.50 | 0.041 | 2.66 | up |
| RT1/RC1 | Salicin 6'-Acetate | Phenolic acids | 1.48 | 0.005 | 1.19 | up |
| RT1/RC1 | Vanilloyl Galloylglucose | Phenolic acids | 1.32 | 0.026 | 1.04 | up |
| RT1/RC1 | 3,4-Dimethylellagic acid 4'-sulfate | Tannins | 1.49 | 0.014 | 1.20 | up |
| RT1/RC1 | tripterifordin | Terpenoids | 1.52 | 0.000 | 5.92 | up |
| RT1/RC1 | Aquilariaene F | Terpenoids | 1.51 | 0.027 | 3.94 | up |
| RT1/RC1 | 9β-hydroxy-(-)-arnebinol B-1-O-β-d-glucopyranoside | Terpenoids | 1.52 | 0.012 | 3.87 | up |
| RT1/RC1 | Eucommioside | Terpenoids | 1.52 | 0.018 | 3.55 | up |
| RT2/RC2 | N1-Methyl-2-pyridone-5-carboxamide | Alkaloids | 1.49 | 0.006 | 4.57 | up |
| RT2/RC2 | Ipratropium | Alkaloids | 1.48 | 0.010 | 4.14 | up |
| RT2/RC2 | Usaramine | Alkaloids | 1.47 | 0.032 | 3.91 | up |
| RT2/RC2 | 4-[2-(1-methylethyl)aminoethyl]phenol | Alkaloids | 1.49 | 0.001 | 1.76 | up |
| RT2/RC2 | Ser-Ile-Asn | Amino acids and derivatives | 1.49 | 0.001 | 4.14 | up |
| RT2/RC2 | Phe-arg | Amino acids and derivatives | 1.47 | 0.045 | 3.56 | up |
| RT2/RC2 | 4-amino-5-(butylamino)-5-oxopentanoic acid | Amino acids and derivatives | 1.46 | 0.035 | 2.85 | up |
| RT2/RC2 | L-Asparaginyl-L-tryptophan | Amino acids and derivatives | 1.31 | 0.009 | 1.54 | up |
| RT2/RC2 | 6-Methoxyquercetin-3-O-Xyloside | Flavonoids | 1.48 | 0.013 | 6.17 | up |
| RT2/RC2 | Chrysin-7-O-glucoside | Flavonoids | 1.48 | 0.012 | 5.19 | up |
| RT2/RC2 | 3',4'-Dihydroxy-7,5'-dimethoxyflavone | Flavonoids | 1.48 | 0.009 | 4.60 | up |
| RT2/RC2 | Chrysoeriol-6,8-di-C-glucoside-4'-O-glucoside | Flavonoids | 1.48 | 0.026 | 4.26 | up |
| RT2/RC2 | methyl 4,8-dihydroxy-6-methoxy-9-oxo-9H-xanthene-3-carboxylate | Flavonoids | 1.47 | 0.046 | 4.24 | up |
| RT2/RC2 | 8-C-xylopyranosylchrysoeriol 2''-O-glucoside | Flavonoids | 1.47 | 0.035 | 4.08 | up |
| RT2/RC2 | Vitexin -4''-O-glucoside | Flavonoids | 1.47 | 0.035 | 4.08 | up |
| RT2/RC2 | Kaempferol-3-O-glucuronide | Flavonoids | 1.46 | 0.050 | 3.39 | up |
| RT2/RC2 | Robinetin | Flavonoids | 1.48 | 0.010 | 3.36 | up |
| RT2/RC2 | Quercetin-sinapyl-glucoside | Flavonoids | 1.47 | 0.045 | 3.21 | up |
| RT2/RC2 | 4',5,8-Trihydroxyflavanone | Flavonoids | 1.49 | 0.002 | 2.83 | up |
| RT2/RC2 | Morin | Flavonoids | 1.46 | 0.048 | 2.71 | up |
| RT2/RC2 | Artocarpanone | Flavonoids | 1.46 | 0.048 | 2.71 | up |
| RT2/RC2 | Retusin 5-O-Glucoside | Flavonoids | 1.46 | 0.047 | 2.70 | up |
| RT2/RC2 | Genistein 7-gentiobioside | Flavonoids | 1.47 | 0.021 | 2.67 | up |
| RT2/RC2 | Tricin-4'-O-(syringyl alcohol)ether-7-O-glucoside | Flavonoids | 1.48 | 0.012 | 2.62 | up |
| RT2/RC2 | Kaempferol-3-O-sambubioside | Flavonoids | 1.48 | 0.013 | 2.60 | up |
| RT2/RC2 | Acerosin | Flavonoids | 1.49 | 0.000 | 2.59 | up |
| RT2/RC2 | Acacetin-7-O-rutinoside (Linarin) | Flavonoids | 1.43 | 0.047 | 2.56 | up |
| RT2/RC2 | Luteolin 7-O-beta-D-glucoside | Flavonoids | 1.48 | 0.016 | 2.55 | up |
| RT2/RC2 | Homobutein 4-glucoside | Flavonoids | 1.48 | 0.016 | 2.55 | up |
| RT2/RC2 | kaempferol-3-caffeoyldiglucoside | Flavonoids | 1.48 | 0.005 | 2.42 | up |
| RT2/RC2 | 4,8,10-trihydroxy-2-methoxy-1h,2h-furo[3,2-a]xanthen-11-one | Flavonoids | 1.49 | 0.001 | 2.36 | up |
| RT2/RC2 | Nepetin (5,7,3',4'-Tetrahydroxy-6-methoxyflavone) | Flavonoids | 1.49 | 0.001 | 2.36 | up |
| RT2/RC2 | Ferreirin | Flavonoids | 1.42 | 0.009 | 2.23 | up |
| RT2/RC2 | Peonidin-3-O-(6''-O-Acetyl)glucoside | Flavonoids | 1.48 | 0.005 | 2.22 | up |
| RT2/RC2 | Chrysin-5-O-glucoside (Toringin) | Flavonoids | 1.29 | 0.014 | 2.02 | up |
| RT2/RC2 | Acacetin-7-O-neohesperidoside | Flavonoids | 1.35 | 0.008 | 1.71 | up |
| RT2/RC2 | Nepetin-8-C-[glucosyl-(1-2)]-glucoside | Flavonoids | 1.25 | 0.008 | 1.69 | up |
| RT2/RC2 | Chrysoeriol | Flavonoids | 1.47 | 0.000 | 1.55 | up |
| RT2/RC2 | Kaempferide | Flavonoids | 1.47 | 0.000 | 1.55 | up |
| RT2/RC2 | Cirsiliol-8-C-glucoside | Flavonoids | 1.25 | 0.028 | 1.35 | up |
| RT2/RC2 | 3'-methylorobol feruloyl glucoside | Flavonoids | 1.44 | 0.005 | 1.32 | up |
| RT2/RC2 | Acacetin-7-O-galactoside | Flavonoids | 1.48 | 0.000 | 1.22 | up |
| RT2/RC2 | Biochanin A-beta-D-glucoside | Flavonoids | 1.48 | 0.000 | 1.22 | up |
| RT2/RC2 | Apigenin-4'-O-glucoside | Flavonoids | 1.32 | 0.010 | 1.20 | up |
| RT2/RC2 | Genistein-7-O-galactoside | Flavonoids | 1.32 | 0.010 | 1.20 | up |
| RT2/RC2 | Apigenin 7-O-beta-D-glucoside | Flavonoids | 1.32 | 0.010 | 1.20 | up |
| RT2/RC2 | Kaempferol-7-O-glucoside | Flavonoids | 1.39 | 0.009 | 1.17 | up |
| RT2/RC2 | Neosakuranin | Flavonoids | 1.39 | 0.009 | 1.17 | up |
| RT2/RC2 | Luteolin-4'-O-glucoside | Flavonoids | 1.39 | 0.009 | 1.17 | up |
| RT2/RC2 | Acacetin-7-O-glucoside (Tilianin) | Flavonoids | 1.47 | 0.014 | 1.15 | up |
| RT2/RC2 | Prunetin-5-O-glucoside | Flavonoids | 1.48 | 0.000 | 1.15 | up |
| RT2/RC2 | Apigenin-7-O-rutinoside (Isorhoifolin) | Flavonoids | 1.39 | 0.012 | 1.11 | up |
| RT2/RC2 | sophorabioside | Flavonoids | 1.40 | 0.022 | 1.10 | up |
| RT2/RC2 | Viscumneoside IV(Rhamnazin-3-O-(6''-hydroxymethylglutaryl)glucoside) | Flavonoids | 1.35 | 0.041 | 1.08 | up |
| RT2/RC2 | Quercetin-3-O-(2''-O-galactosyl)glucoside | Flavonoids | 1.31 | 0.014 | 1.06 | up |
| RT2/RC2 | Herbacetin-3-O-glucuronide | Flavonoids | 1.46 | 0.019 | 1.06 | up |
| RT2/RC2 | Fraxidinglucoside | Lignans and Coumarins | 1.48 | 0.025 | 4.00 | up |
| RT2/RC2 | Bursehernin | Lignans and Coumarins | 1.49 | 0.001 | 2.97 | up |
| RT2/RC2 | Decursinol | Lignans and Coumarins | 1.27 | 0.032 | 1.18 | up |
| RT2/RC2 | LysoPC 20:0 | Lipids | 1.48 | 0.035 | 4.63 | up |
| RT2/RC2 | 2-Aminooctadecane-1,5,17-triol | Lipids | 1.48 | 0.007 | 2.02 | up |
| RT2/RC2 | 2-Aminooctadecane-1,16,18,18-tetraol | Lipids | 1.48 | 0.002 | 1.81 | up |
| RT2/RC2 | 2-AminoicoSane-1,5,7,19-tetraol | Lipids | 1.47 | 0.000 | 1.81 | up |
| RT2/RC2 | 2-aminodocoSane-1,6,19,20,21-pentaol | Lipids | 1.45 | 0.003 | 1.71 | up |
| RT2/RC2 | Myristoyl Ethanolamide | Lipids | 1.47 | 0.002 | 1.64 | up |
| RT2/RC2 | 2-Aminooctadecane-1,5,7,17-tetraol | Lipids | 1.46 | 0.000 | 1.61 | up |
| RT2/RC2 | Hexadecylsphingosine | Lipids | 1.46 | 0.003 | 1.48 | up |
| RT2/RC2 | 2-Aminohexadecane-1,4-diol | Lipids | 1.48 | 0.001 | 1.47 | up |
| RT2/RC2 | 2-Aminohexadecane-1,15-diol | Lipids | 1.48 | 0.000 | 1.46 | up |
| RT2/RC2 | 2-aminohexadec-4-ene-1,3-diol | Lipids | 1.47 | 0.001 | 1.38 | up |
| RT2/RC2 | 2-Aminotetradecane-1,5,13-triol | Lipids | 1.28 | 0.019 | 1.37 | up |
| RT2/RC2 | 2-Aminohexadecane-1,5,15-triol | Lipids | 1.40 | 0.010 | 1.34 | up |
| RT2/RC2 | 2-Aminotetradecan-1-ol* | Lipids | 1.48 | 0.001 | 1.21 | up |
| RT2/RC2 | 2-AminoicoSane-1,6,18,19,20-pentaol | Lipids | 1.42 | 0.014 | 1.18 | up |
| RT2/RC2 | 2-Aminohexadecane-1,5,6-triol | Lipids | 1.41 | 0.002 | 1.12 | up |
| RT2/RC2 | 2-Amino-7-methyltridecan-1-ol | Lipids | 1.47 | 0.001 | 1.06 | up |
| RT2/RC2 | 2-AminoicoSane-1,5,19-triol | Lipids | 1.25 | 0.027 | 1.06 | up |
| RT2/RC2 | [(2R)-2-(8-carboxyoctanoyloxy)-3-hexadecanoyloxypropyl] 2-(trimethylazaniumyl)ethyl phosphate | Lipids | 1.48 | 0.002 | 1.03 | up |
| RT2/RC2 | 1,7-Dimethylxanthine | Nucleotides and derivatives | 1.47 | 0.034 | 3.64 | up |
| RT2/RC2 | FAD | Nucleotides and derivatives | 1.25 | 0.010 | 2.18 | up |
| RT2/RC2 | Ftaxilide | Organic acids | 1.46 | 0.042 | 2.97 | up |
| RT2/RC2 | Triethyl citrate | Organic acids | 1.47 | 0.005 | 1.65 | up |
| RT2/RC2 | 2,4,6-Trihydroxybenzophenone | Others | 1.48 | 0.049 | 4.69 | up |
| RT2/RC2 | 2-(Butoxycarbonyl)benzoic acid | Others | 1.46 | 0.049 | 4.08 | up |
| RT2/RC2 | Resveratroloside | Others | 1.49 | 0.003 | 3.94 | up |
| RT2/RC2 | 3'-Norspongiolactone | Others | 1.49 | 0.003 | 3.85 | up |
| RT2/RC2 | 2-Hydroxy-7-carboxy-1-methyl-5-ethenyl-9,10-dihydrophenanthrene | Others | 1.47 | 0.037 | 3.64 | up |
| RT2/RC2 | 2-Hydroxy-8-carboxy-1-methyl-5-ethenyl-9,10-dihydrophenanthrene | Others | 1.49 | 0.001 | 3.41 | up |
| RT2/RC2 | Imperanene glucoside | Others | 1.48 | 0.023 | 3.34 | up |
| RT2/RC2 | 5,8-dihydroxy-1-hydroxymethylnaphtho[2,3-c]furan-4,9-dione | Others | 1.48 | 0.008 | 3.08 | up |
| RT2/RC2 | 3,4-Dihydroxyphenethyl alcohol-8-O-[4-O-caffeoyl-β-D-apinosyl(1→3)-β-D-glucosyl(1→6)]-β-D-glucoside | Others | 1.47 | 0.025 | 2.91 | up |
| RT2/RC2 | 1-Octen-3-ol-3-o-beta-D-xylopyranosyl(1->6)-beta-D-glucopyranoside | Others | 1.48 | 0.004 | 2.48 | up |
| RT2/RC2 | tenuiflorin C | Others | 1.49 | 0.001 | 2.36 | up |
| RT2/RC2 | Riboflavin | Others | 1.46 | 0.000 | 1.58 | up |
| RT2/RC2 | Lauryldiethanolamine | Others | 1.48 | 0.001 | 1.54 | up |
| RT2/RC2 | Neoligustilide | Others | 1.34 | 0.039 | 1.51 | up |
| RT2/RC2 | Phylloquinone | Others | 1.43 | 0.011 | 1.44 | up |
| RT2/RC2 | 2-(hydroxymethyl)-6-[(3-methoxy-6a,11a-dihydro-6H-[1]benzofuro[3,2-c]chromen-9-yl)oxy]oxane-3,4,5-triol | Others | 1.32 | 0.010 | 1.20 | up |
| RT2/RC2 | 4-Cresol | Others | 1.40 | 0.045 | 1.16 | up |
| RT2/RC2 | Bilobol | Phenolic acids | 1.49 | 0.017 | 6.35 | up |
| RT2/RC2 | 3,5-Dihydroxyphenyl1-O-(6-O-Galloyl-β-D-Glucopyranoside) | Phenolic acids | 1.48 | 0.013 | 3.91 | up |
| RT2/RC2 | 3-(3-Hydroxyphenyl)-3-hydroxypropanoic acid | Phenolic acids | 1.49 | 0.001 | 3.90 | up |
| RT2/RC2 | 4-Hydroxybenzoyl-1-O-(6''-O-galloyl)glucoside | Phenolic acids | 1.49 | 0.002 | 3.74 | up |
| RT2/RC2 | Ningposide A | Phenolic acids | 1.49 | 0.003 | 3.29 | up |
| RT2/RC2 | 3-O-p-Coumaroylquinic acid-O-glucoside | Phenolic acids | 1.48 | 0.009 | 3.00 | up |
| RT2/RC2 | 1-O-Caffeoyl-(6-O-glucosyl)-β-D-glucose | Phenolic acids | 1.49 | 0.002 | 2.91 | up |
| RT2/RC2 | 1,5-O-dicaffeoyl-3-O-dimethylmalyl-quinic acid | Phenolic acids | 1.47 | 0.025 | 2.75 | up |
| RT2/RC2 | (S)-Mandelate | Phenolic acids | 1.48 | 0.006 | 2.49 | up |
| RT2/RC2 | 3,5-Di-O-caffeoyl-1-O-(4-O-glucosylmaloyl)-quinic acid | Phenolic acids | 1.42 | 0.031 | 1.16 | up |
| RT2/RC2 | Phenylacetylglycerol 2-glucuronide | Phenolic acids | 1.34 | 0.025 | 1.04 | up |
| RT2/RC2 | 1,3-O-Di-p-Coumaroylglycerol | Phenolic acids | 1.48 | 0.001 | 1.04 | up |
| RT2/RC2 | 17beta-hydroxy-3-oxo-19-nor-5alpha-androst-1-ene | Steroids | 1.46 | 0.043 | 3.00 | up |
| RT2/RC2 | Curcumanggoside | Terpenoids | 1.49 | 0.001 | 3.80 | up |
| RT2/RC2 | alpha-Ionone | Terpenoids | 1.48 | 0.013 | 3.28 | up |
| RT2/RC2 | [(4S,5R,9S,10R,13S,14R)-5-methyl-5-pentacyclo[11.2.1.01,10.04,9.012,14]hexadecanyl]methanol | Terpenoids | 1.47 | 0.038 | 3.15 | up |
| RT2/RC2 | Aquilariaene F | Terpenoids | 1.46 | 0.047 | 2.82 | up |
| RT2/RC2 | 6-O-Sinapoylajugol | Terpenoids | 1.48 | 0.005 | 2.60 | up |
| RT2/RC2 | Loliolide | Terpenoids | 1.44 | 0.034 | 1.26 | up |

**Table S5: Upregulated metabolites of DAMs in ST1/SC1 and ST2/SC2**

| Comparison group | Compounds | Class | VIP | P-value | Log2FC | Type |
| --- | --- | --- | --- | --- | --- | --- |
| ST1/SC1 | N-Sinapoylmethylagmatine | Alkaloids | 1.57 | 0.002 | 4.31 | up |
| ST1/SC1 | Menisdaurine | Alkaloids | 1.57 | 0.018 | 3.71 | up |
| ST1/SC1 | 3-Methyldioxyindole | Alkaloids | 1.57 | 0.015 | 3.15 | up |
| ST1/SC1 | Terrestriamide | Alkaloids | 1.56 | 0.024 | 3.13 | up |
| ST1/SC1 | caffeoylputrescine | Alkaloids | 1.57 | 0.014 | 3.11 | up |
| ST1/SC1 | Di-p-coumaroylputrescine | Alkaloids | 1.54 | 0.048 | 2.96 | up |
| ST1/SC1 | tryptamineisovalerate | Alkaloids | 1.56 | 0.026 | 2.84 | up |
| ST1/SC1 | N-trans-cinnamoylphydroxyphenylethylamine | Alkaloids | 1.56 | 0.021 | 2.59 | up |
| ST1/SC1 | dihydro-N-feruloyltyramine | Alkaloids | 1.57 | 0.004 | 2.53 | up |
| ST1/SC1 | N-Isopentenyl-6-hydroxydendroxinium | Alkaloids | 1.57 | 0.005 | 2.50 | up |
| ST1/SC1 | 3-(3-hydroxyphenyl)-N-((E)-2-(5-methoxycyclopenta-1,3-dien-1-yl)vinyl)acrylamide | Alkaloids | 1.55 | 0.006 | 2.48 | up |
| ST1/SC1 | cis-N-p-coumaroyltyramine | Alkaloids | 1.55 | 0.006 | 2.48 | up |
| ST1/SC1 | 3-(4-hydroxyphenyl)-n-[2-(4-hydroxyphenyl)ethyl]prop-2-enimidic acid | Alkaloids | 1.55 | 0.014 | 2.38 | up |
| ST1/SC1 | N-p-Coumaroyltyramine | Alkaloids | 1.56 | 0.001 | 2.35 | up |
| ST1/SC1 | L-Palmitoylcarnitine | Alkaloids | 1.46 | 0.026 | 2.06 | up |
| ST1/SC1 | N-Benzoylanthranilate | Alkaloids | 1.50 | 0.021 | 1.43 | up |
| ST1/SC1 | Feruloylagmatine | Alkaloids | 1.56 | 0.008 | 1.43 | up |
| ST1/SC1 | Tribulusamide A | Alkaloids | 1.54 | 0.010 | 1.19 | up |
| ST1/SC1 | (7z)-N-(4'-hydroxyphenethyl)-3-methoxy-4-hydroxycinnamamide | Alkaloids | 1.50 | 0.005 | 1.06 | up |
| ST1/SC1 | N-Phenethylbenzamide | Alkaloids | 1.53 | 0.014 | 1.00 | up |
| ST1/SC1 | Ser-Ile-Asn | Amino acids and derivatives | 1.57 | 0.013 | 4.32 | up |
| ST1/SC1 | Phe-Gln | Amino acids and derivatives | 1.57 | 0.007 | 4.12 | up |
| ST1/SC1 | Val-Val-Asp | Amino acids and derivatives | 1.57 | 0.013 | 3.88 | up |
| ST1/SC1 | Val-Ile-Asp | Amino acids and derivatives | 1.56 | 0.014 | 2.57 | up |
| ST1/SC1 | N5-(1-Iminoethyl)-L-ornithine | Amino acids and derivatives | 1.57 | 0.009 | 2.52 | up |
| ST1/SC1 | Glucosyl-rhamnazin-3-O-β-D-glucoside | Flavonoids | 1.57 | 0.012 | 11.99 | up |
| ST1/SC1 | Helichrysetin | Flavonoids | 1.57 | 0.005 | 6.49 | up |
| ST1/SC1 | Neosakuranetin | Flavonoids | 1.57 | 0.004 | 6.16 | up |
| ST1/SC1 | 5,4'-Dihydroxy-7,3'-dimethoxyflavanone | Flavonoids | 1.57 | 0.005 | 5.53 | up |
| ST1/SC1 | Tricin-7-O-Glucoside-4'-O-Caffeoylglycerol | Flavonoids | 1.57 | 0.007 | 5.43 | up |
| ST1/SC1 | Sakuranetin | Flavonoids | 1.57 | 0.007 | 5.09 | up |
| ST1/SC1 | 5,7,8-Tetrahydroxy-6-methoxyflavone | Flavonoids | 1.57 | 0.009 | 5.07 | up |
| ST1/SC1 | Persicogenin (5,3'-dihydroxy-7,4'-dimethoxyflavanone) | Flavonoids | 1.57 | 0.018 | 4.88 | up |
| ST1/SC1 | Acacetin-7-O-glucoside (Tilianin) | Flavonoids | 1.57 | 0.007 | 4.28 | up |
| ST1/SC1 | 6,7-Dihydroxyflavone | Flavonoids | 1.57 | 0.015 | 4.13 | up |
| ST1/SC1 | Ferreirin | Flavonoids | 1.57 | 0.005 | 4.10 | up |
| ST1/SC1 | Acacetin-7-O-galactoside | Flavonoids | 1.57 | 0.011 | 3.42 | up |
| ST1/SC1 | Biochanin A-beta-D-glucoside | Flavonoids | 1.57 | 0.011 | 3.42 | up |
| ST1/SC1 | Prunetin-5-O-glucoside | Flavonoids | 1.57 | 0.005 | 3.27 | up |
| ST1/SC1 | Oxygenated Xanthohumol | Flavonoids | 1.57 | 0.002 | 3.21 | up |
| ST1/SC1 | 1-hydroxy-7-{[(2s,3r,4s,5r,6r)-3,4,5-trihydroxy-6-(hydroxymethyl)oxan-2-yl]oxy}xanthen-9-one | Flavonoids | 1.55 | 0.040 | 3.21 | up |
| ST1/SC1 | Petunidin-3-O-arabinoside | Flavonoids | 1.55 | 0.048 | 3.08 | up |
| ST1/SC1 | Dihydrokaempferol | Flavonoids | 1.55 | 0.037 | 2.80 | up |
| ST1/SC1 | 5,2'-Dihydroxy-7-methoxyflavanone | Flavonoids | 1.57 | 0.008 | 2.53 | up |
| ST1/SC1 | 4',5-Dihydroxy-3',5'-dimethoxyflavone | Flavonoids | 1.57 | 0.001 | 2.38 | up |
| ST1/SC1 | Acacetin | Flavonoids | 1.57 | 0.008 | 2.08 | up |
| ST1/SC1 | Apigenin-8-C-Arabinoside | Flavonoids | 1.51 | 0.016 | 1.55 | up |
| ST1/SC1 | Apigenin-6-C-xyloside-8-C-arabinoside | Flavonoids | 1.55 | 0.002 | 1.45 | up |
| ST1/SC1 | Tectochrysin | Flavonoids | 1.42 | 0.008 | 1.28 | up |
| ST1/SC1 | Tricin-7-O-(6'-O-glucoside)glucuronicacid | Flavonoids | 1.54 | 0.002 | 1.23 | up |
| ST1/SC1 | Apigenin-6,8-di-C-arabinoside | Flavonoids | 1.55 | 0.007 | 1.22 | up |
| ST1/SC1 | Apigenin-6-C-arabinoside-8-C-xyloside | Flavonoids | 1.53 | 0.001 | 1.12 | up |
| ST1/SC1 | Galangin-8-C-Arabinoside | Flavonoids | 1.54 | 0.000 | 1.05 | up |
| ST1/SC1 | Apigenin-6-C-arabinoside | Flavonoids | 1.54 | 0.000 | 1.05 | up |
| ST1/SC1 | Diosmetin-7-O-glucuronide | Flavonoids | 1.27 | 0.027 | 1.04 | up |
| ST1/SC1 | Luteolin-8-C-arabinoside | Flavonoids | 1.44 | 0.010 | 1.01 | up |
| ST1/SC1 | Phlorizin | Flavonoids | 1.27 | 0.046 | 1.00 | up |
| ST1/SC1 | Erythro-Guaiacylglycerol-β-Sinapyl Ether | Lignans and Coumarins | 1.57 | 0.009 | 4.60 | up |
| ST1/SC1 | Mandshurin | Lignans and Coumarins | 1.57 | 0.021 | 4.17 | up |
| ST1/SC1 | (+)-Lyoniresinol 9'-O-glucoside | Lignans and Coumarins | 1.54 | 0.000 | 1.41 | up |
| ST1/SC1 | Myristicanol B | Lignans and Coumarins | 1.55 | 0.010 | 1.19 | up |
| ST1/SC1 | Fragransin B1 | Lignans and Coumarins | 1.57 | 0.003 | 1.18 | up |
| ST1/SC1 | Tortoside B | Lignans and Coumarins | 1.55 | 0.006 | 1.13 | up |
| ST1/SC1 | Phenprocoumon | Lignans and Coumarins | 1.34 | 0.028 | 1.09 | up |
| ST1/SC1 | 7-O-Prenyl demethylSuberosin | Lignans and Coumarins | 1.53 | 0.002 | 1.06 | up |
| ST1/SC1 | Tomenin | Lignans and Coumarins | 1.36 | 0.016 | 1.04 | up |
| ST1/SC1 | LysoPC 22:4 | Lipids | 1.57 | 0.012 | 4.43 | up |
| ST1/SC1 | LysoPC 22:5(2n isomer) | Lipids | 1.57 | 0.016 | 3.47 | up |
| ST1/SC1 | LysoPC 18:4 | Lipids | 1.57 | 0.016 | 3.19 | up |
| ST1/SC1 | Phosphatidylethanolaminelysoalkenyl16:0 | Lipids | 1.55 | 0.029 | 2.76 | up |
| ST1/SC1 | Glycidyl oleate | Lipids | 1.56 | 0.018 | 2.68 | up |
| ST1/SC1 | LysoPE 20:4(2n isomer) | Lipids | 1.54 | 0.004 | 1.85 | up |
| ST1/SC1 | (E)-12-hydroxydodec-2-enoic acid | Lipids | 1.57 | 0.002 | 1.57 | up |
| ST1/SC1 | Arachidonate | Lipids | 1.40 | 0.010 | 1.49 | up |
| ST1/SC1 | 5(S)-Hydroperoxyeicosatetraenoic acid | Lipids | 1.57 | 0.000 | 1.23 | up |
| ST1/SC1 | Hydroxyicosanoic Acid | Lipids | 1.55 | 0.006 | 1.20 | up |
| ST1/SC1 | LysoPE 15:1 | Lipids | 1.43 | 0.005 | 1.19 | up |
| ST1/SC1 | Corchorifatty acid B | Lipids | 1.51 | 0.028 | 1.13 | up |
| ST1/SC1 | LysoPC 20:4 | Lipids | 1.44 | 0.024 | 1.03 | up |
| ST1/SC1 | Ethyl isobutyrate | Organic acids | 1.55 | 0.048 | 3.97 | up |
| ST1/SC1 | Pentanoate | Organic acids | 1.55 | 0.036 | 3.02 | up |
| ST1/SC1 | Adipate | Organic acids | 1.54 | 0.020 | 1.30 | up |
| ST1/SC1 | (3E,5E,8Z,11Z)-10,13,15-trimethylheptadeca-3,5,8,11-tetraenoic acid | Others | 1.57 | 0.015 | 4.42 | up |
| ST1/SC1 | N-methylanthraniloyl-beta-D-glucose | Others | 1.57 | 0.018 | 3.71 | up |
| ST1/SC1 | 3-(2-hydroxyethyl)-5,7-dimethoxy-4-methyl-2H-1-benzopyran-2-one | Others | 1.55 | 0.048 | 3.55 | up |
| ST1/SC1 | Benzyl alcohol xylopyranosyl-(1-6)-glucopyranoside | Others | 1.56 | 0.031 | 3.54 | up |
| ST1/SC1 | 3-(1-hydroxyethyl)-4-methylpentane-1,4-diol O-Glucoside | Others | 1.56 | 0.028 | 3.41 | up |
| ST1/SC1 | Pyramidatin F | Others | 1.54 | 0.046 | 3.04 | up |
| ST1/SC1 | 3-Hydroxy-1-(4-Hydroxy-3-Methoxyphenyl)Propan-1-One | Others | 1.56 | 0.026 | 2.96 | up |
| ST1/SC1 | 6,7-dimethoxy-2-[2-(4'-hydroxy-3'-methoxyphenyl)ethyl]chromone | Others | 1.54 | 0.039 | 2.95 | up |
| ST1/SC1 | 2-Ethynylbenzaldehyde | Others | 1.55 | 0.038 | 2.82 | up |
| ST1/SC1 | all-trans-13,14-Dihydroretinol | Others | 1.56 | 0.025 | 2.67 | up |
| ST1/SC1 | (3E,5E,8Z,11Z)-7,10,15-trimethylheptadeca-3,5,8,11-tetraenoic acid | Others | 1.56 | 0.013 | 2.54 | up |
| ST1/SC1 | ent-17-Hydroxykaur-15-en-19-oic acid | Others | 1.57 | 0.000 | 2.00 | up |
| ST1/SC1 | 6-phenyl-hexan-2-ol | Others | 1.55 | 0.000 | 1.53 | up |
| ST1/SC1 | Soraphen O | Others | 1.51 | 0.004 | 1.30 | up |
| ST1/SC1 | 2-Amino-4-methylvaleric acid | Others | 1.28 | 0.050 | 1.03 | up |
| ST1/SC1 | Methyl-[4]-Shogaol | Phenolic acids | 1.57 | 0.008 | 4.58 | up |
| ST1/SC1 | Benzoyl-Beta-D-Glucoside | Phenolic acids | 1.57 | 0.018 | 4.21 | up |
| ST1/SC1 | 4''-O-Acetylverbascoside | Phenolic acids | 1.56 | 0.034 | 3.48 | up |
| ST1/SC1 | 3-hydroxy-5-methoxybenzaldehyde | Phenolic acids | 1.57 | 0.000 | 3.44 | up |
| ST1/SC1 | Methylgallic Acid 3-(6''-Sulfate)Glucoside | Phenolic acids | 1.57 | 0.002 | 3.04 | up |
| ST1/SC1 | Casuarinondiol | Phenolic acids | 1.56 | 0.018 | 2.99 | up |
| ST1/SC1 | 1-Galloyl-6-O-Benzoyl Glucose | Phenolic acids | 1.53 | 0.025 | 1.13 | up |
| ST1/SC1 | Cryptochlorogenic acid (4-O-Caffeoylquinic acid) | Phenolic acids | 1.53 | 0.001 | 1.09 | up |
| ST1/SC1 | 3,4,5-Trimethoxyphenol-1-O-β-D-malonylglucoside | Phenolic acids | 1.30 | 0.022 | 1.03 | up |
| ST1/SC1 | peganoneⅠ | Quinones | 1.55 | 0.003 | 1.45 | up |
| ST1/SC1 | Nudifloid H | Terpenoids | 1.56 | 0.032 | 4.72 | up |
| ST1/SC1 | 13-Hydroxy-9(11),16-Kauradien-19-Oic Acid | Terpenoids | 1.57 | 0.007 | 4.23 | up |
| ST1/SC1 | 6,16-Kauradien-19-Oic Acid | Terpenoids | 1.57 | 0.011 | 4.02 | up |
| ST1/SC1 | 2,10-dihydroxy-11a-methyl-hexadecahydro-1H-cyclopenta[a]phenanthren-7-one | Terpenoids | 1.56 | 0.019 | 3.80 | up |
| ST1/SC1 | taepeenin K | Terpenoids | 1.55 | 0.037 | 3.70 | up |
| ST1/SC1 | 2-Hydroxy-Pimara-5,15-dien-19-oic acid | Terpenoids | 1.56 | 0.032 | 3.70 | up |
| ST1/SC1 | 11-Hydroxyjasmonic acid | Terpenoids | 1.55 | 0.035 | 3.68 | up |
| ST1/SC1 | Oxyphyllenone A | Terpenoids | 1.57 | 0.004 | 3.43 | up |
| ST1/SC1 | Isopimaric acid | Terpenoids | 1.55 | 0.041 | 3.32 | up |
| ST1/SC1 | epishyobunone | Terpenoids | 1.56 | 0.023 | 3.25 | up |
| ST1/SC1 | 3,19-Epoxy-3,22-dihydroxydammara-20,24-dien-26-oic acid δ-lactone (Semialactone) | Terpenoids | 1.57 | 0.014 | 3.22 | up |
| ST1/SC1 | Dehydro-4-Epiabietal | Terpenoids | 1.57 | 0.005 | 3.15 | up |
| ST1/SC1 | 1-(5-hydroxy-4-methylpenta-1,3-dienyl)-1,7-dimethyl-4-methylidene-1a,2,3,4a,5,6,7a,7b-octahydrocyclopropa[h]azulen-7-ol | Terpenoids | 1.57 | 0.006 | 3.10 | up |
| ST1/SC1 | 4-[5-(3-Hydroxypropyl)-7-methoxy-3-methyl-2,3-dihydro-1-benzofuran-2-yl]-2-methoxyphenol | Terpenoids | 1.56 | 0.021 | 3.09 | up |
| ST1/SC1 | [(4aR,5R,8aS)-1-(hydroxymethyl)-4a-methyl-6-methylidene-5-[(2E)-3-methylpenta-2,4-dien-1-yl]-decahydronaphthalen-1-yl]methanol | Terpenoids | 1.54 | 0.046 | 2.74 | up |
| ST1/SC1 | 1-(hydroxymethyl)-1,4a-dimethyl-7-propan-2-yl-2,3,4,9,10,10a-hexahydrophenanthren-9-ol | Terpenoids | 1.55 | 0.030 | 2.73 | up |
| ST1/SC1 | Soyasapogenol E | Terpenoids | 1.57 | 0.011 | 2.60 | up |
| ST1/SC1 | Mililatensol A | Terpenoids | 1.56 | 0.013 | 2.59 | up |
| ST1/SC1 | (2S,3S,5R)-2-[[(1R,4aR,8aR)-5,5,8a-trimethyl-2-methylene-decalin-1-yl]methyl]-5-methoxy-tetrahydrofuran-3-carbaldehyde | Terpenoids | 1.56 | 0.017 | 2.57 | up |
| ST1/SC1 | Abieta-8,11,13-trien-3-one | Terpenoids | 1.57 | 0.003 | 2.46 | up |
| ST1/SC1 | Estriol | Terpenoids | 1.53 | 0.000 | 2.33 | up |
| ST1/SC1 | 1,7-Dioxo-3-hydroxy-Pimara-8,15-dien-19-oic acid | Terpenoids | 1.51 | 0.011 | 2.27 | up |
| ST1/SC1 | Gibberellin A24 | Terpenoids | 1.51 | 0.011 | 2.27 | up |
| ST1/SC1 | 2,7-Dioxo-3-hydroxy-Pimara-8,15-dien-19-oic acid | Terpenoids | 1.54 | 0.001 | 2.26 | up |
| ST1/SC1 | Epirosmanol | Terpenoids | 1.56 | 0.010 | 1.96 | up |
| ST1/SC1 | 2,3-Dihydroxy-6,18-epoxypimara-8,15-dien-7,18-dione | Terpenoids | 1.55 | 0.004 | 1.85 | up |
| ST1/SC1 | Kaur-16-en-18-oic acid | Terpenoids | 1.56 | 0.001 | 1.66 | up |
| ST1/SC1 | Levopimaric acid | Terpenoids | 1.55 | 0.005 | 1.66 | up |
| ST1/SC1 | 3-Oxo-9beta-pimara-8,15-dien-19,6beta-olide | Terpenoids | 1.56 | 0.002 | 1.66 | up |
| ST1/SC1 | Oryzalexin C | Terpenoids | 1.52 | 0.001 | 1.61 | up |
| ST1/SC1 | 2,3,3a,4,5,6-Hexahydro-1,4-dimethylazulen-4-ol | Terpenoids | 1.55 | 0.000 | 1.60 | up |
| ST1/SC1 | 3-Hydroxyabieta-8,11,13-trien-7-one | Terpenoids | 1.52 | 0.001 | 1.55 | up |
| ST1/SC1 | 2,7-Dioxo-pimara-15-en-3,6,19-triol | Terpenoids | 1.45 | 0.003 | 1.55 | up |
| ST1/SC1 | Isorosmanol | Terpenoids | 1.52 | 0.004 | 1.52 | up |
| ST1/SC1 | Epiisorosmanol | Terpenoids | 1.52 | 0.004 | 1.52 | up |
| ST1/SC1 | 8-ethyl-1,1,4a,7-tetramethyl-1,2,3,4,4a,9,10,10a-octahydrophenanthren-2-one | Terpenoids | 1.50 | 0.001 | 1.51 | up |
| ST1/SC1 | (1R,4R,5S,9S,13R)-5,9-Dimethyl-14-Methylidenetetracyclo[11.2.1.01,10.04,9]Hexadec-10-Ene-5-Carboxylic Acid | Terpenoids | 1.55 | 0.000 | 1.42 | up |
| ST1/SC1 | 7-Oxo-3-hydroxypimaric acid | Terpenoids | 1.56 | 0.001 | 1.42 | up |
| ST1/SC1 | sphaeropsidin C | Terpenoids | 1.57 | 0.001 | 1.37 | up |
| ST1/SC1 | 13-Hydroxy-2-Oxo-8,11,13-Totaratrien-19-Oic Acid | Terpenoids | 1.54 | 0.003 | 1.32 | up |
| ST1/SC1 | 2-Hydroxy-6,18-epoxypimara-8,15-dien-7,18-dione | Terpenoids | 1.54 | 0.003 | 1.32 | up |
| ST1/SC1 | Inflexarabdonin J | Terpenoids | 1.31 | 0.032 | 1.29 | up |
| ST1/SC1 | pimara-5,8,15-trien-2,19-diol | Terpenoids | 1.41 | 0.016 | 1.25 | up |
| ST1/SC1 | 7-Oxo-pimara-1,8a,11,15-Tetraen-19-ol | Terpenoids | 1.46 | 0.048 | 1.25 | up |
| ST1/SC1 | Geranyl acetate | Terpenoids | 1.53 | 0.007 | 1.25 | up |
| ST1/SC1 | Aldovibsanin A | Terpenoids | 1.56 | 0.002 | 1.24 | up |
| ST1/SC1 | Siegesbeckic acid | Terpenoids | 1.54 | 0.001 | 1.22 | up |
| ST1/SC1 | Lathyrol | Terpenoids | 1.57 | 0.003 | 1.22 | up |
| ST1/SC1 | 7-Oxo-3-hydroxyabietic acid | Terpenoids | 1.50 | 0.018 | 1.17 | up |
| ST1/SC1 | Hispanone | Terpenoids | 1.48 | 0.011 | 1.16 | up |
| ST1/SC1 | 7-hydroxy-1,4a-dimethyl-3-oxo-8-propan-2-yl-4,9,10,10a-tetrahydro-2H-phenanthrene-1-carboxylic acid | Terpenoids | 1.48 | 0.028 | 1.15 | up |
| ST1/SC1 | ent-7alpha-Hydroxykaur-16-en-19-oic acid | Terpenoids | 1.53 | 0.013 | 1.11 | up |
| ST1/SC1 | 7-isopropyl-1,1,4a-trimethyl-4,9,10,10a-tetrahydro-3h-phenanthren-2-one | Terpenoids | 1.55 | 0.000 | 1.04 | up |
| ST1/SC1 | Aspewentin A | Terpenoids | 1.43 | 0.017 | 1.04 | up |
| ST1/SC1 | 2,5,8-trimethylnona-1,4,7-triene-1,9-diol 9-O-Glyceryllinolenate | Terpenoids | 1.52 | 0.004 | 1.02 | up |
| ST1/SC1 | 18-Hydroxy-8(14),15-Isopimaradien-2-one | Terpenoids | 1.40 | 0.015 | 1.01 | up |
| ST2/SC2 | [8-acetyloxy-11-ethyl-5-hydroxy-6,16,18-trimethoxy-13-(methoxymethyl)-11-azahexacyclo[7.7.2.12,5.01,10.03,8.013,17]nonadecan-4-yl]benzoate | Alkaloids | 1.60 | 0.048 | 3.80 | up |
| ST2/SC2 | Piperettine | Alkaloids | 1.60 | 0.025 | 2.62 | up |
| ST2/SC2 | 4-[2-(1-methylethyl)aminoethyl]phenol | Alkaloids | 1.61 | 0.000 | 1.73 | up |
| ST2/SC2 | 4-O-Methyl-DIBOA-Glucoside | Alkaloids | 1.44 | 0.016 | 1.05 | up |
| ST2/SC2 | Avenanthramide D | Alkaloids | 1.59 | 0.011 | 1.05 | up |
| ST2/SC2 | (4-methyl-2-(tyrosyloxy)pentanoyl)glutamine | Amino acids and derivatives | 1.60 | 0.033 | 3.96 | up |
| ST2/SC2 | Chrysoeriol | Flavonoids | 1.61 | 0.010 | 4.45 | up |
| ST2/SC2 | Kaempferide | Flavonoids | 1.61 | 0.010 | 4.45 | up |
| ST2/SC2 | 4',5,6,7-Tetramethoxyflavone | Flavonoids | 1.61 | 0.010 | 3.49 | up |
| ST2/SC2 | Taxifolin | Flavonoids | 1.61 | 0.012 | 3.13 | up |
| ST2/SC2 | 3',4',7-Trihydroxyflavone | Flavonoids | 1.60 | 0.035 | 2.98 | up |
| ST2/SC2 | 2,6,7,4'-Tetrahydroxyisoflavanone | Flavonoids | 1.59 | 0.043 | 2.93 | up |
| ST2/SC2 | (+)-Lyoniresinol 9'-O-glucoside | Lignans and Coumarins | 1.62 | 0.005 | 4.01 | up |
| ST2/SC2 | Isolariciresinol-9'-O-glucoside | Lignans and Coumarins | 1.61 | 0.010 | 3.10 | up |
| ST2/SC2 | 1,4-Benzodioxin-6-propanol | Lignans and Coumarins | 1.52 | 0.003 | 1.01 | up |
| ST2/SC2 | 1-Palmitoyl-2-acetyl-sn-glycero-3-phosphocholine | Lipids | 1.61 | 0.016 | 4.91 | up |
| ST2/SC2 | 1-Palmitoylglycerol 3-phosphate | Lipids | 1.60 | 0.034 | 3.49 | up |
| ST2/SC2 | LysoPE 18:4 | Lipids | 1.60 | 0.027 | 2.82 | up |
| ST2/SC2 | Arachidonate | Lipids | 1.61 | 0.008 | 2.77 | up |
| ST2/SC2 | (E)-12-hydroxydodec-2-enoic acid | Lipids | 1.60 | 0.010 | 1.87 | up |
| ST2/SC2 | 9-OxoODE | Lipids | 1.60 | 0.002 | 1.01 | up |
| ST2/SC2 | cyclic ADP-ribose | Nucleotides and derivatives | 1.61 | 0.016 | 3.76 | up |
| ST2/SC2 | 2-Phosphoglycolate | Organic acids | 1.60 | 0.021 | 2.83 | up |
| ST2/SC2 | 3'-Norspongiolactone | Others | 1.60 | 0.007 | 4.17 | up |
| ST2/SC2 | 3-(1-hydroxyethyl)-4-methylpentane-1,4-diol O-Glucoside | Others | 1.62 | 0.002 | 3.93 | up |
| ST2/SC2 | Senkyunolide K | Others | 1.61 | 0.009 | 3.57 | up |
| ST2/SC2 | alpha-Cyperone | Others | 1.61 | 0.028 | 3.49 | up |
| ST2/SC2 | 3-Hydroxy-1-(4-Hydroxy-3-Methoxyphenyl)Propan-1-One | Others | 1.60 | 0.024 | 3.28 | up |
| ST2/SC2 | 4-methylbenzenesulfonic octadeca-14,17-dienoic anhydride | Others | 1.58 | 0.041 | 2.98 | up |
| ST2/SC2 | D-Arabinose | Others | 1.59 | 0.037 | 2.93 | up |
| ST2/SC2 | L-Xylose | Others | 1.59 | 0.037 | 2.93 | up |
| ST2/SC2 | Icariside F2 | Others | 1.60 | 0.031 | 2.72 | up |
| ST2/SC2 | 2,4-Dinitrophenol | Others | 1.59 | 0.015 | 1.75 | up |
| ST2/SC2 | 2-Hydroxy-8-carboxy-1-methyl-5-ethenyl-9,10-dihydrophenanthrene | Others | 1.60 | 0.004 | 1.36 | up |
| ST2/SC2 | 6-phenyl-hexan-2-ol | Others | 1.46 | 0.015 | 1.30 | up |
| ST2/SC2 | 2-Hydroxy-7-carboxy-1-methyl-5-ethenyl-9,10-dihydrophenanthrene | Others | 1.60 | 0.013 | 1.30 | up |
| ST2/SC2 | Bilobol | Phenolic acids | 1.62 | 0.004 | 4.94 | up |
| ST2/SC2 | 2-[(3,4,5,6-tetrahydroxyoxan-2-yl)methoxy]benzoic acid | Phenolic acids | 1.61 | 0.020 | 4.52 | up |
| ST2/SC2 | Methyl cinnamate | Phenolic acids | 1.61 | 0.031 | 3.84 | up |
| ST2/SC2 | Phthalic acid,6-ethyl-3-octylbutyl ester | Phenolic acids | 1.62 | 0.005 | 3.83 | up |
| ST2/SC2 | 7-Hydroxycalamenene | Phenolic acids | 1.59 | 0.047 | 3.76 | up |
| ST2/SC2 | 3,6'-Disinapoylsucrose | Phenolic acids | 1.62 | 0.003 | 3.25 | up |
| ST2/SC2 | Dihydroferulic Acid | Phenolic acids | 1.61 | 0.009 | 3.15 | up |
| ST2/SC2 | Asarylaldehyde; 2,4,5-Trimethoxybenzaldehyde | Phenolic acids | 1.61 | 0.017 | 3.08 | up |
| ST2/SC2 | Kelampayoside A[3,4,5-Trimethoxyphenol-β-D-apiosyl-(1→6)-β-D-glucoside] | Phenolic acids | 1.58 | 0.045 | 2.99 | up |
| ST2/SC2 | Trans-Ferulic Acid 1'-O-[Apiosyl-(1→6)-Glucosyl] Ester | Phenolic acids | 1.59 | 0.038 | 2.69 | up |
| ST2/SC2 | Benzoyl-Beta-D-Glucoside | Phenolic acids | 1.60 | 0.021 | 2.52 | up |
| ST2/SC2 | 19-Norpregna-4,17(20)-dien-3-one | Terpenoids | 1.62 | 0.008 | 4.01 | up |
| ST2/SC2 | 10-(hydroxymethyl)-6,12a-dimethyl-3-prop-1-en-2-yl-2,3,3a,4,7,8,11,12-octahydro-1H-cyclopenta[11]annulen-11-ol | Terpenoids | 1.61 | 0.018 | 3.82 | up |
| ST2/SC2 | Aristolone | Terpenoids | 1.60 | 0.029 | 3.54 | up |
| ST2/SC2 | 3,19-Epoxy-3,22-dihydroxydammara-20,24-dien-26-oic acid δ-lactone (Semialactone) | Terpenoids | 1.62 | 0.006 | 3.34 | up |
| ST2/SC2 | 15,16-Dihydroxy-7-Isopimaren-3-one | Terpenoids | 1.60 | 0.041 | 3.14 | up |
| ST2/SC2 | 3-Acetoxy-9,13-epoxy-16-hydroxy-labda-15,16-olide | Terpenoids | 1.61 | 0.006 | 3.11 | up |
| ST2/SC2 | Zerumbetol | Terpenoids | 1.59 | 0.041 | 2.96 | up |
| ST2/SC2 | [(4aR,5R,8aS)-1-(hydroxymethyl)-4a-methyl-6-methylidene-5-[(2E)-3-methylpenta-2,4-dien-1-yl]-decahydronaphthalen-1-yl]methanol | Terpenoids | 1.61 | 0.013 | 2.96 | up |
| ST2/SC2 | tripterifordin | Terpenoids | 1.61 | 0.006 | 2.82 | up |
| ST2/SC2 | 7-Oxoabietic acid | Terpenoids | 1.59 | 0.041 | 2.63 | up |
| ST2/SC2 | 2,3,3a,4,5,6-Hexahydro-1,4-dimethylazulen-4-ol | Terpenoids | 1.59 | 0.001 | 1.79 | up |
| ST2/SC2 | Geranyl acetate | Terpenoids | 1.42 | 0.017 | 1.60 | up |
| ST2/SC2 | (8R)-9-hydroxy-8-(hydroxymethyl)-6-methoxy-8-methylpyrano[2,3-f]chromen-2-one | Terpenoids | 1.50 | 0.042 | 1.06 | up |

**Table S6: DAMs with overlapping** **T0/C0∩T1/C1∩T2/C2**

| **Class** | **Name** | **T1/C1** | | **T2/C2** | | **T0/C0** | |
| --- | --- | --- | --- | --- | --- | --- | --- |
|  |  | P-value | Log2FC | P-value | Log2FC | P-value | Log2FC |
| **Shoot** | | | | | |  |  |
| Alkaloids | 2-Glucosyloxy-4-hydroxybenzeneacetonitrile | 0.004 | -2.44 | 0.001 | -2.91 | 0.001 | 2.98 |
| Flavonoids | 5,2'-Dihydroxy-7-methoxyflavanone | 0.008 | 2.53 | 0.002 | -1.31 | 0.033 | -2.78 |
|  | Andrographidine D aglycone | 0.017 | -2.74 | 0.010 | -1.11 | 0.004 | 5.44 |
|  | Helichrysetin | 0.005 | 6.49 | 0.000 | -1.17 | 0.000 | -2.66 |
|  | Neosakuranetin | 0.004 | 6.16 | 0.000 | -1.19 | 0.001 | -2.66 |
|  | Sakuranetin | 0.007 | 5.09 | 0.001 | -1.15 | 0.002 | -2.59 |
|  | Tricin-7-O-Glucoside-4'-O-Caffeoylglycerol | 0.007 | 5.43 | 0.016 | -6.19 | 0.008 | -2.69 |
| Others | 3-Hydroxy-1-(4-Hydroxy-3-Methoxyphenyl)Propan-1-One | 0.026 | 2.96 | 0.024 | 3.28 | 0.008 | -2.45 |
| Phenolic acids | 7-Hydroxycalamenene | 0.042 | -2.86 | 0.047 | 3.76 | 0.014 | -3.52 |
|  | Caffeate | 0.020 | -1.68 | 0.001 | -4.10 | 0.001 | -1.51 |
| Terpenoids | 10-(hydroxymethyl)-6,12a-dimethyl-3-prop-1-en-2-yl-2,3,3a,4,7,8,11,12-octahydro-1H-cyclopenta[11]annulen-11-ol | 0.037 | -3.08 | 0.018 | 3.82 | 0.049 | -3.64 |
|  | Mililatensol A | 0.013 | 2.59 | 0.002 | -2.98 | 0.021 | -3.11 |
| **Root** | | | | | |  |  |
| Alkaloids | Hexadecyl ethanolamine | 0.017 | -3.88 | 0.022 | -2.74 | 0.001 | -1.15 |
|  | N1-Methyl-2-pyridone-5-carboxamide | 0.039 | 4.48 | 0.006 | 4.57 | 0.006 | -5.53 |
| Flavonoids | 6-Methylflavone | 0.007 | -1.75 | 0.008 | -2.39 | 0.000 | -2.33 |
|  | Chrysin-5-O-glucoside (Toringin) | 0.045 | 3.05 | 0.014 | 2.02 | 0.000 | -2.98 |
|  | Cirsimaritin-8-C-glucoside | 0.024 | -4.19 | 0.041 | -2.76 | 0.003 | -4.39 |
|  | Tricin-4'-O-Vanillicaldehyde | 0.019 | -2.71 | 0.022 | -4.68 | 0.007 | -2.72 |
| Lipids | (2E,4E,6E)-dodeca-2,4,6-trienoic acid | 0.001 | -1.64 | 0.002 | -2.51 | 0.002 | -2.60 |
|  | [(2R)-2-(8-carboxyoctanoyloxy)-3-hexadecanoyloxypropyl] 2-(trimethylazaniumyl)ethyl phosphate | 0.013 | 1.06 | 0.002 | 1.03 | 0.009 | 1.08 |
|  | 2-Aminohexadecane-1,5,15-triol | 0.030 | 1.02 | 0.010 | 1.34 | 0.002 | 1.36 |
|  | 2-Aminohexadecane-1,5,6-triol | 0.000 | 1.37 | 0.002 | 1.12 | 0.001 | 1.04 |
| Nucleotides and derivatives | 3',5'-Cyclic AMP | 0.006 | -1.21 | 0.000 | -1.22 | 0.002 | -2.78 |
|  | Adenosine 2',3'-cyclic phosphate | 0.041 | -1.13 | 0.002 | -1.74 | 0.001 | -2.70 |
| Organic acids | Ibufenac | 0.000 | -1.73 | 0.000 | -2.49 | 0.006 | -2.38 |
| Others | 3,4-dihydroxyallylbenzene | 0.000 | -2.02 | 0.000 | -2.99 | 0.003 | -2.26 |
|  | 4-Phenethylphenol | 0.000 | -1.74 | 0.000 | -2.33 | 0.004 | -2.47 |
|  | 5-methoxy-2-(3-methylbut-2-enyl)benzene-1,3-diol | 0.001 | -1.03 | 0.006 | -2.19 | 0.002 | -1.18 |
|  | Aplidiasphingosine | 0.000 | -1.17 | 0.012 | -1.13 | 0.029 | 1.17 |
|  | Benzhydryl methyl ether | 0.003 | -1.71 | 0.000 | -2.34 | 0.000 | -2.52 |
|  | Butylidenephthalide | 0.008 | -1.12 | 0.000 | -2.08 | 0.000 | -1.37 |
|  | Cnidilide | 0.000 | -1.81 | 0.005 | -2.30 | 0.000 | -2.92 |
|  | Dehydronerolisovalerate | 0.023 | -1.22 | 0.001 | -2.50 | 0.000 | -1.71 |
|  | ethyl 4-phenylbutanoate | 0.001 | -1.65 | 0.000 | -2.27 | 0.001 | -2.48 |
|  | Neocnidilide | 0.000 | -1.81 | 0.005 | -2.33 | 0.004 | -2.66 |
|  | Senkyunolide K | 0.028 | -1.03 | 0.004 | -2.50 | 0.001 | -1.50 |
|  | Senkyunolide | 0.000 | -1.68 | 0.000 | -2.29 | 0.001 | -2.32 |
| Phenolic acids | 3-Spinoyl-6'-acetyl-sucrose | 0.013 | 2.96 | 0.043 | -3.27 | 0.023 | 2.70 |
|  | 4-Isopropylcinnamic acid | 0.000 | -2.00 | 0.001 | -3.12 | 0.001 | -2.44 |
|  | beta-Asarone | 0.002 | -1.69 | 0.000 | -2.47 | 0.000 | -2.08 |
|  | Bilobol | 0.038 | -1.28 | 0.017 | 6.35 | 0.001 | 8.38 |
| Quinones | Anthraquinone | 0.002 | -2.52 | 0.007 | -2.76 | 0.000 | -3.77 |
| Terpenoids | 1-(hydroxymethyl)-1,7-dimethyl-1a,2,3,5,6,7,7a,7b-octahydrocyclopropa[e]azulene-4-carbaldehyde | 0.018 | -4.59 | 0.005 | -1.51 | 0.018 | -5.07 |
|  | 12-Hydroxy-9,11(13)-Eremophiladien-8-one | 0.007 | -1.30 | 0.000 | -1.84 | 0.000 | -2.50 |
|  | 2,6-Dimethyldeca-2,4,6,8-tetraenedial | 0.003 | -2.05 | 0.002 | -3.08 | 0.000 | -2.27 |
|  | 2-[(2β,4αβ,8β,8αβ)-decahydro-4α-hydroxy-8,8α-dimethylnaphthalen-2-yl]prop-2-enal | 0.007 | -1.14 | 0.006 | -2.10 | 0.006 | -1.34 |
|  | 2-Hydroxy-1(10)-Aromadendren-14-al | 0.007 | -1.32 | 0.004 | -1.79 | 0.003 | -2.39 |
|  | Aristelegone A | 0.000 | -1.85 | 0.006 | -3.17 | 0.002 | -2.52 |
|  | Aristolone | 0.000 | -1.84 | 0.000 | -2.44 | 0.000 | -2.54 |
|  | Epirosmanol | 0.021 | -2.73 | 0.035 | -3.21 | 0.023 | -3.34 |
|  | Humulene-6,7-Oxide | 0.001 | -1.19 | 0.002 | -2.02 | 0.008 | -1.73 |
|  | Isocurcumenol | 0.001 | -1.39 | 0.007 | -5.10 | 0.028 | -1.32 |
|  | Menthadienyl acetate | 0.009 | -1.47 | 0.000 | -1.96 | 0.000 | -1.92 |
|  | Neopetasane | 0.031 | -1.26 | 0.001 | -2.29 | 0.010 | -2.13 |
|  | Nootkatone | 0.001 | -1.06 | 0.003 | -1.96 | 0.009 | -1.14 |
|  | Selina-3,7(11)-dien-8-one | 0.002 | -1.34 | 0.002 | -2.14 | 0.000 | -1.84 |
|  | tricyclo[7.3.1.02,7]tridec-2(7)-en-13-ol | 0.004 | -1.48 | 0.001 | -2.18 | 0.001 | -2.53 |

**Table S7：**Correlation between module metabolites and traits

| modules | RC0 | RC1 | RC2 | SC0 | SC1 | SC2 | RT0 | RT1 | RT2 | ST0 | ST1 | ST2 | RC | SC | RT | ST |
| --- | --- | --- | --- | --- | --- | --- | --- | --- | --- | --- | --- | --- | --- | --- | --- | --- |
| black | -0.10 | -0.05 | -0.02 | -0.42 | -0.15 | 0.56 | 0.02 | -0.06 | -0.06 | -0.39 | 0.03 | 0.63 | -0.11 | 0.00 | -0.06 | 0.17 |
| greenyellow | 0.01 | -0.14 | -0.48 | 0.15 | 0.06 | 0.36 | -0.06 | -0.17 | -0.56 | 0.04 | 0.29 | 0.50 | -0.39 | 0.36 | -0.50 | 0.53 |
| turquoise | -0.32 | -0.30 | -0.29 | 0.31 | 0.29 | 0.28 | -0.29 | -0.30 | -0.30 | 0.32 | 0.31 | 0.30 | -0.59 | 0.56 | -0.57 | 0.59 |
| pink | 0.30 | 0.09 | -0.65 | 0.11 | 0.04 | 0.05 | 0.33 | 0.09 | -0.64 | 0.09 | 0.09 | 0.09 | -0.17 | 0.13 | -0.14 | 0.18 |
| red | 0.42 | 0.18 | -0.30 | -0.04 | -0.18 | -0.38 | 0.60 | 0.30 | -0.16 | 0.04 | -0.11 | -0.36 | 0.19 | -0.38 | 0.47 | -0.28 |
| green | 0.42 | 0.22 | -0.06 | -0.21 | -0.46 | 0.32 | 0.05 | 0.13 | -0.14 | -0.56 | -0.09 | 0.39 | 0.36 | -0.22 | 0.02 | -0.16 |
| tan | 0.67 | 0.46 | -0.05 | -0.21 | -0.14 | 0.08 | 0.02 | 0.11 | -0.20 | -0.51 | -0.16 | -0.09 | 0.69 | -0.17 | -0.04 | -0.48 |
| blue | 0.31 | 0.32 | 0.28 | -0.32 | -0.36 | -0.22 | 0.30 | 0.31 | 0.27 | -0.36 | -0.30 | -0.23 | 0.58 | -0.57 | 0.56 | -0.57 |
| purple | 0.36 | 0.37 | 0.59 | 0.14 | -0.29 | -0.36 | -0.09 | 0.03 | 0.06 | -0.29 | -0.30 | -0.23 | 0.84 | -0.32 | 0.00 | -0.52 |

**Table S8：**Module trait Pvalue

| modules | R1 | R2 | R3 | S1 | S2 | S3 | R4 | R5 | R6 | S4 | S5 | S6 | RC | SC | RT | ST |
| --- | --- | --- | --- | --- | --- | --- | --- | --- | --- | --- | --- | --- | --- | --- | --- | --- |
| black | 0.54423 | 0.75941 | 0.92037 | 0.01176 | 0.38250 | 0.00038 | 0.88678 | 0.72351 | 0.73814 | 0.01834 | 0.85411 | 0.00003 | 0.51751 | 0.98502 | 0.72765 | 0.30785 |
| greenyellow | 0.97408 | 0.42903 | 0.00336 | 0.38447 | 0.73770 | 0.03002 | 0.73671 | 0.32474 | 0.00035 | 0.82841 | 0.08446 | 0.00202 | 0.01975 | 0.02939 | 0.00172 | 0.00095 |
| turquoise | 0.05643 | 0.07055 | 0.08137 | 0.06905 | 0.08670 | 0.09360 | 0.08173 | 0.07802 | 0.07954 | 0.06092 | 0.06410 | 0.07471 | 0.00017 | 0.00037 | 0.00032 | 0.00014 |
| pink | 0.07415 | 0.61733 | 0.00002 | 0.53778 | 0.79952 | 0.76986 | 0.05069 | 0.58899 | 0.00002 | 0.59474 | 0.58463 | 0.59019 | 0.33616 | 0.45694 | 0.41236 | 0.29913 |
| red | 0.01181 | 0.29697 | 0.07647 | 0.83313 | 0.29372 | 0.02383 | 0.00011 | 0.07818 | 0.33990 | 0.83705 | 0.50973 | 0.03134 | 0.27163 | 0.02301 | 0.00391 | 0.09917 |
| green | 0.01162 | 0.20272 | 0.71747 | 0.21640 | 0.00519 | 0.05810 | 0.77843 | 0.46296 | 0.41394 | 0.00042 | 0.60029 | 0.01869 | 0.02887 | 0.19244 | 0.89876 | 0.33896 |
| tan | 0.00001 | 0.00465 | 0.75241 | 0.22564 | 0.41348 | 0.64084 | 0.88903 | 0.50763 | 0.25296 | 0.00164 | 0.35530 | 0.59635 | 0.00000 | 0.32004 | 0.83200 | 0.00287 |

**Figure. S1** Total ion current (TIC) overlay of quality control samples from mass spectrometry detection. (a) negative-ion mode, (b) positive-ion mode.

**
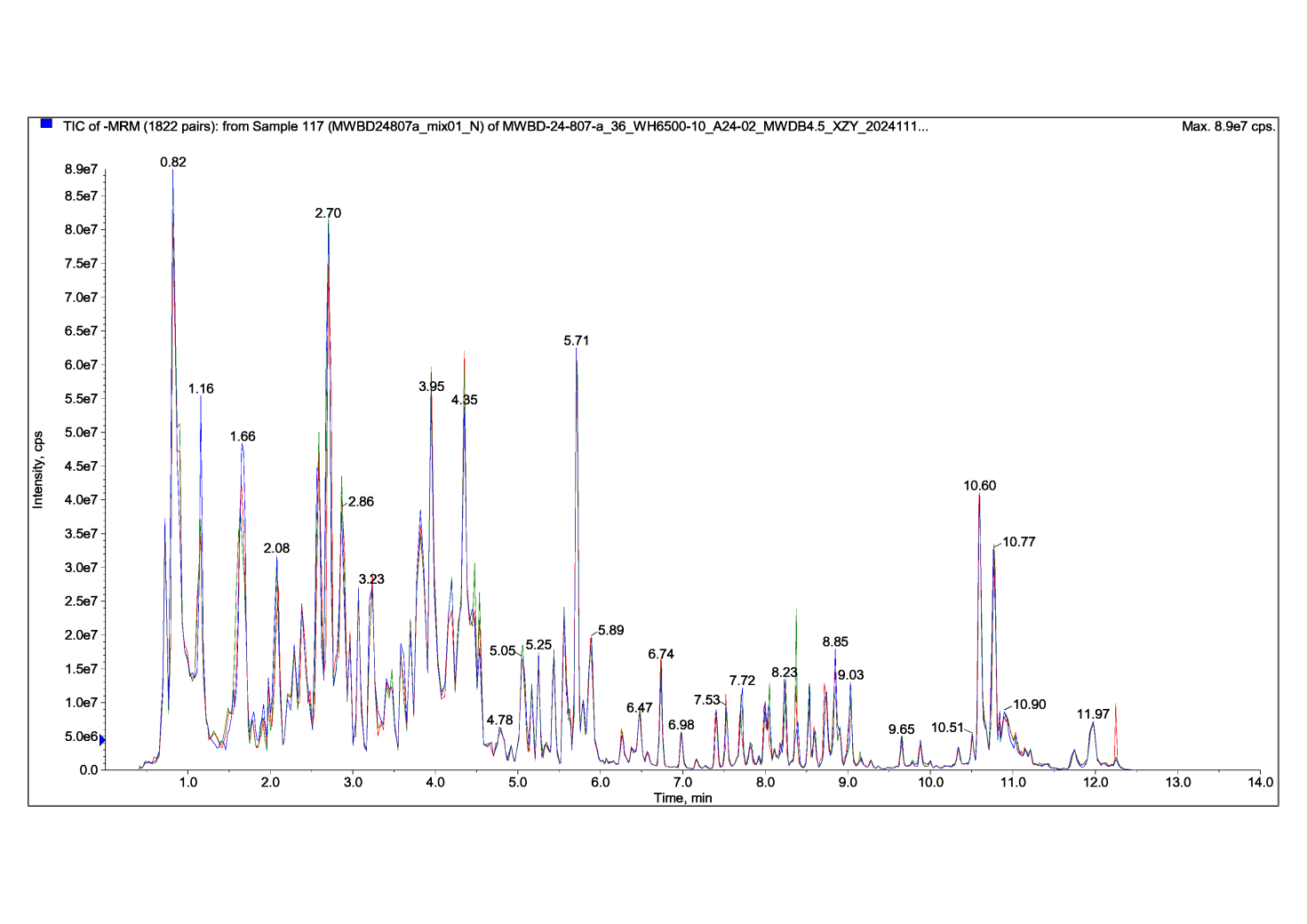
**

1. QC_MS_TIC_overlap-N

**
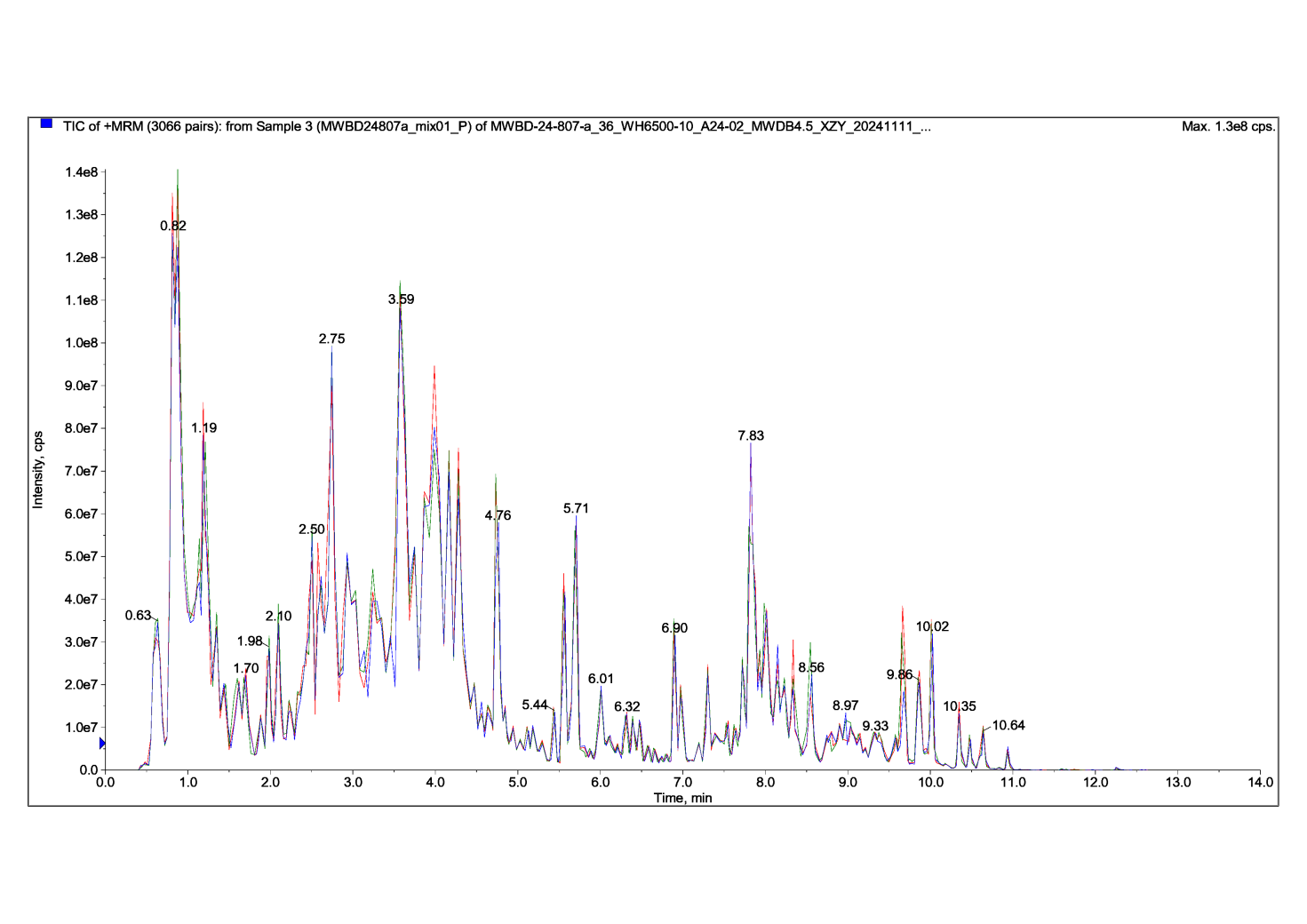
**

1. QC_MS_TIC_overlap-P

Note: Overlay of from QC sample mass spectrometry detection. The results demonstrate high curve overlap in metabolite detection total ion current, with consistent retention times and peak intensities, indicating excellent signal stability when the same samples were analyzed at different time points. The high instrument stability provides essential assurance for data reproducibility and reliability, where N represents negative ion mode and P represents positive ion mode.

**Figure. S2** Multi-peak chromatograms of metabolite detection via MRM. The x-axis represents the retention time (RT) of metabolite detection, and the y-axis represents the ion intensity (in counts per second, cps). (a) negative-ion mode, (b) positive-ion mode.


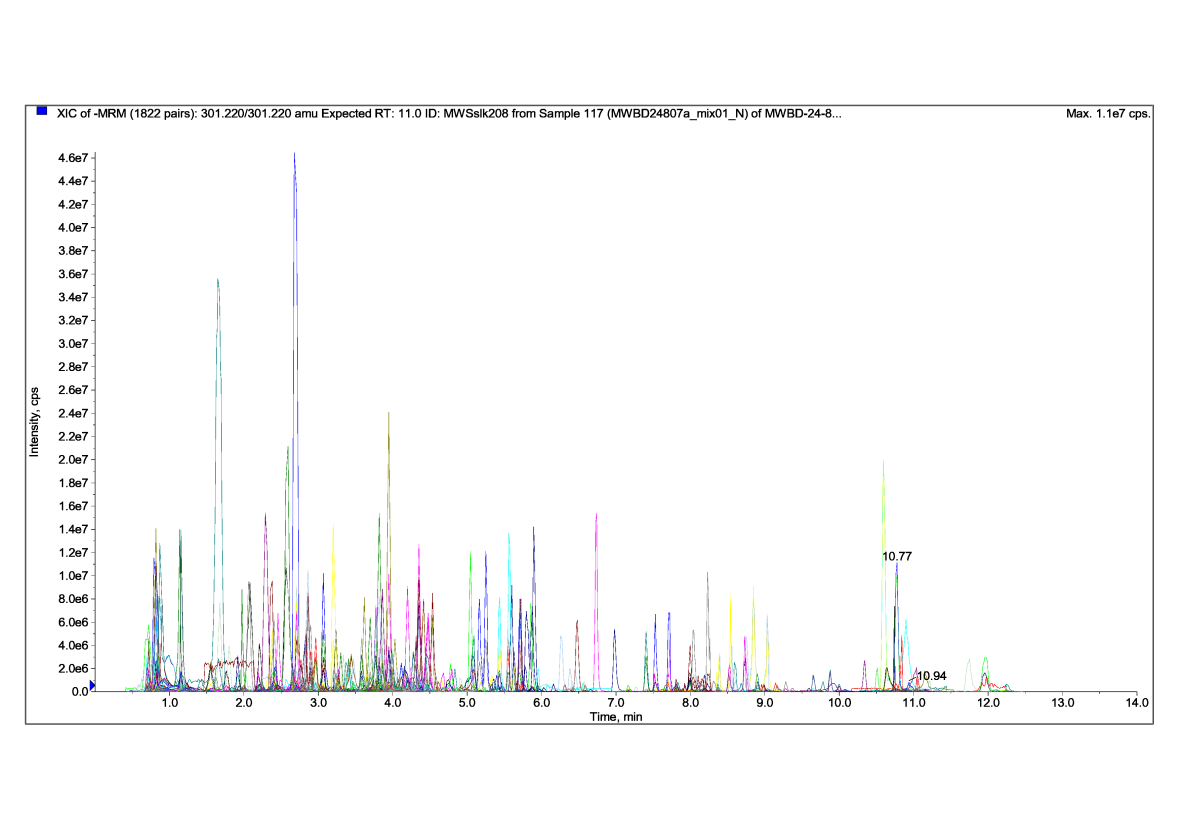


1. MRM_detection_of_multimodal_maps-N


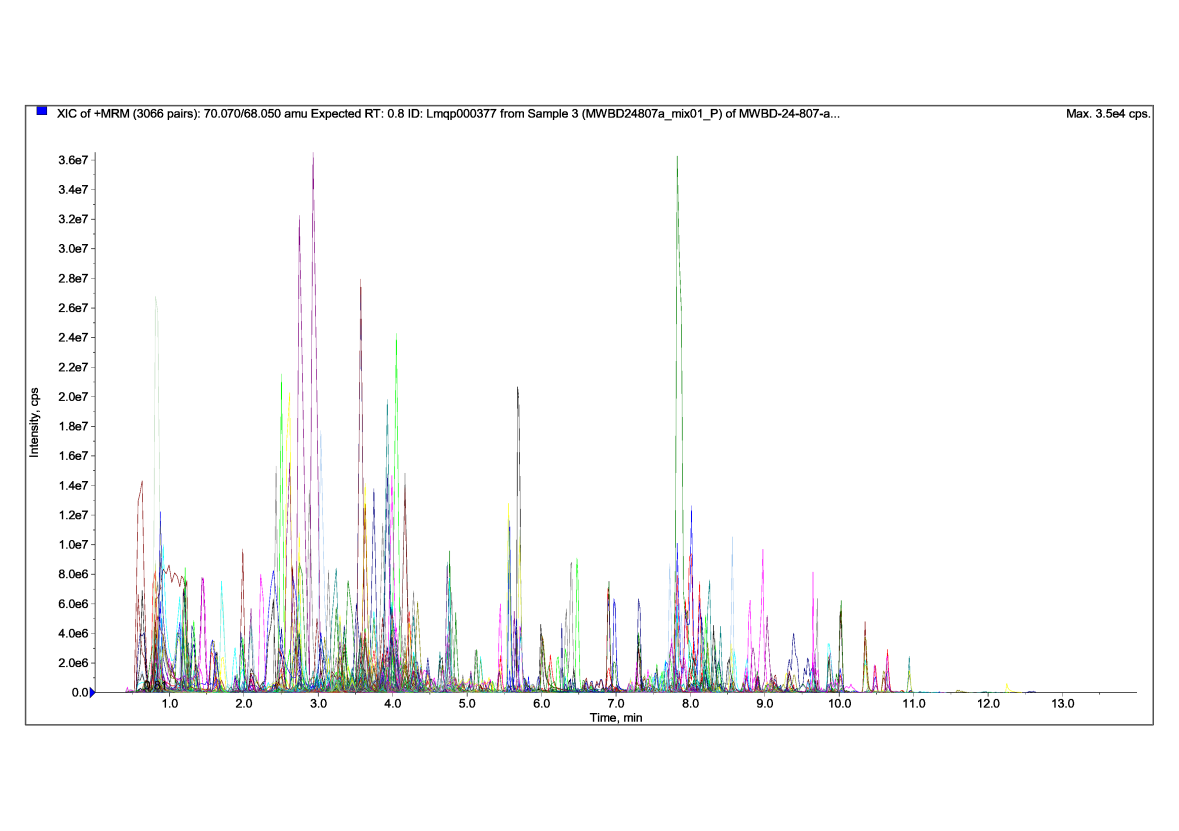


1. MRM_detection_of_multimodal_maps-P

Note: N represents negative ion mode; P represents positive ion mode

**Figure. S3** Metabolite quantification integration correction plot. (a) negative-ion mode, (b) positive-ion mode.


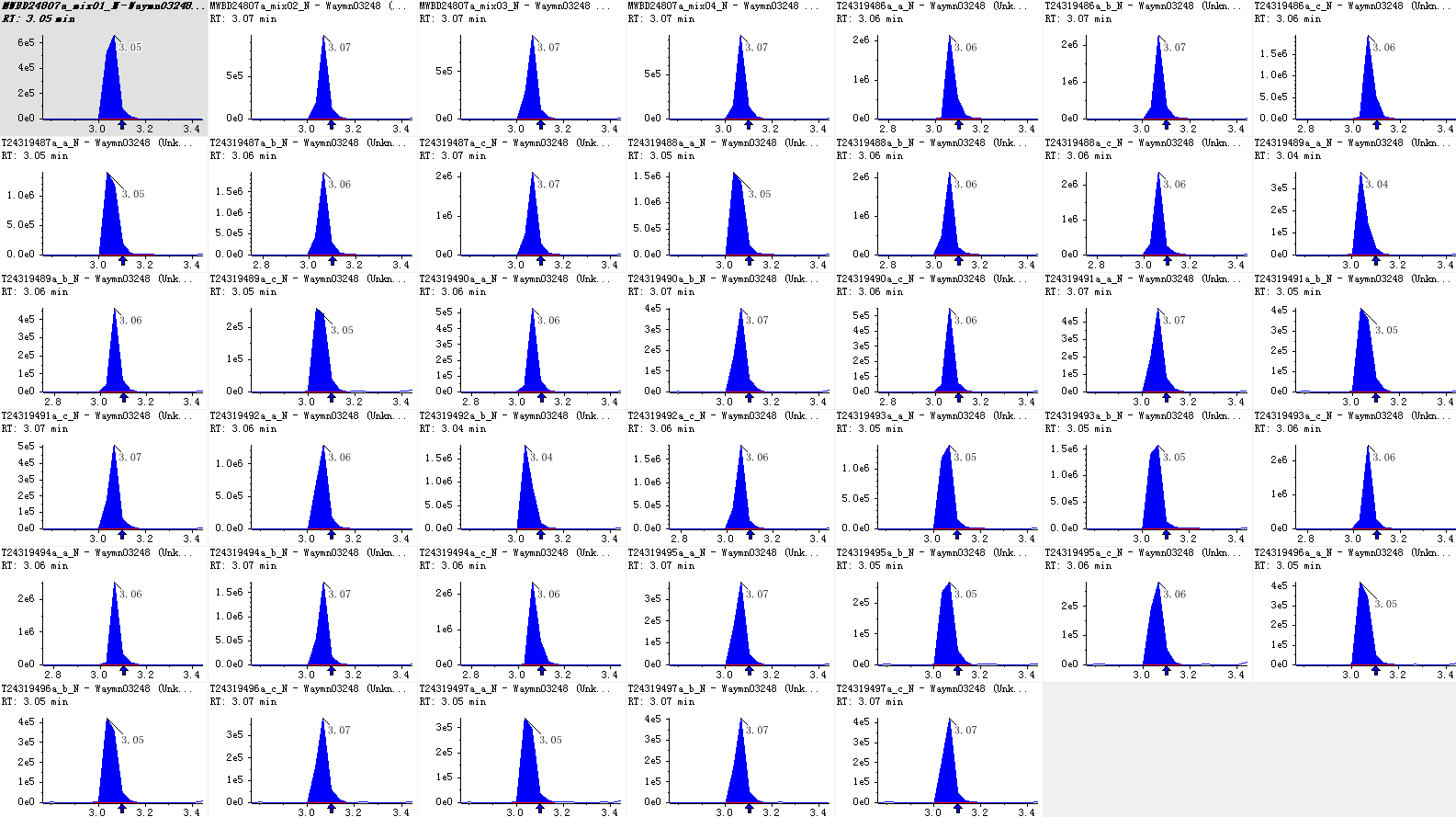


(a) Integral_correction_diagram-N


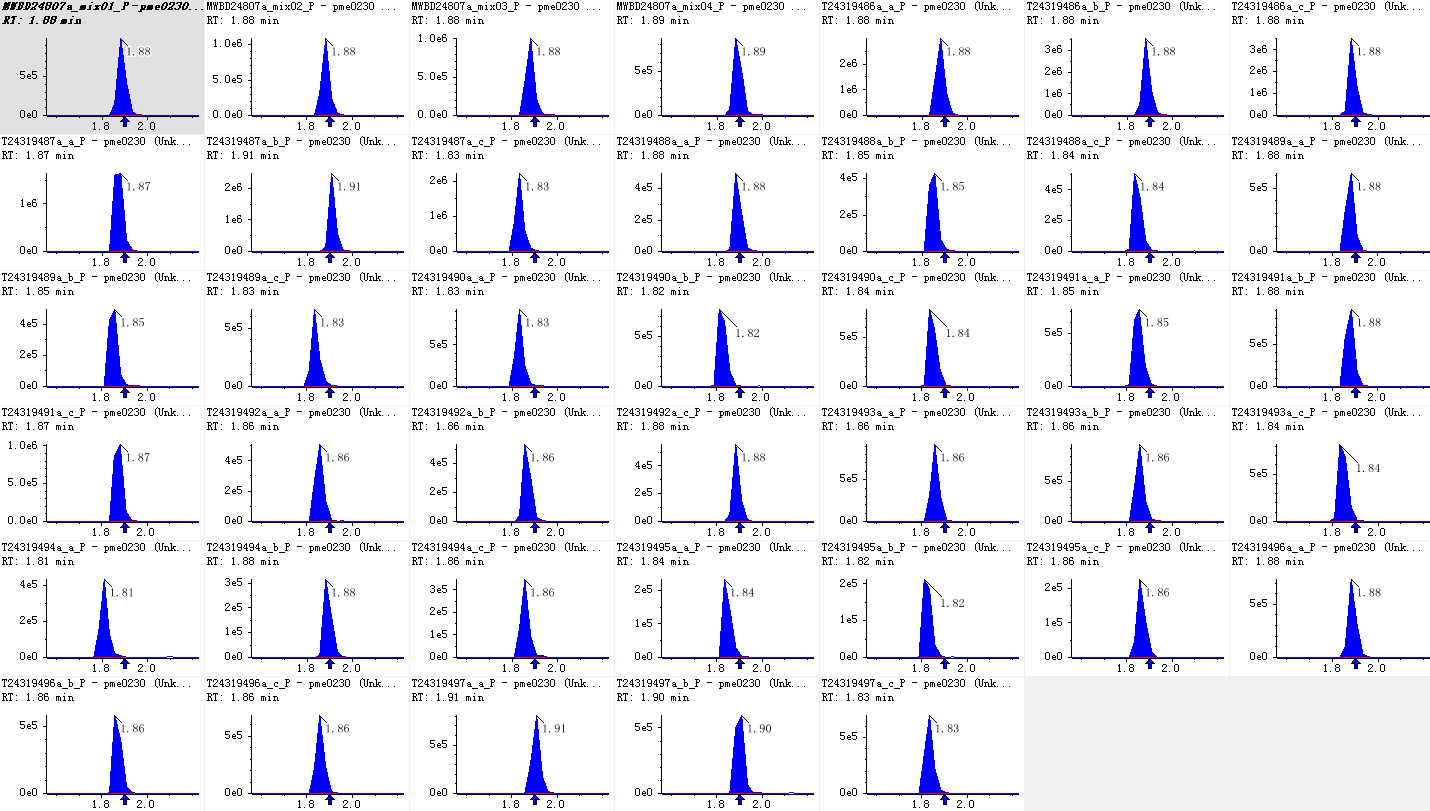


(b) Integral_correction_diagram-P

Note: The figure shows integration correction results for randomly selected metabolites across different samples. The x-axis indicates the retention time of metabolite detection (min), and the y-axis indicates the ion intensity of a given metabolite (cps). Peak area represents the relative abundance of the substance in the sample.

**Figure. S4** CV distribution of samples across all groups.

**
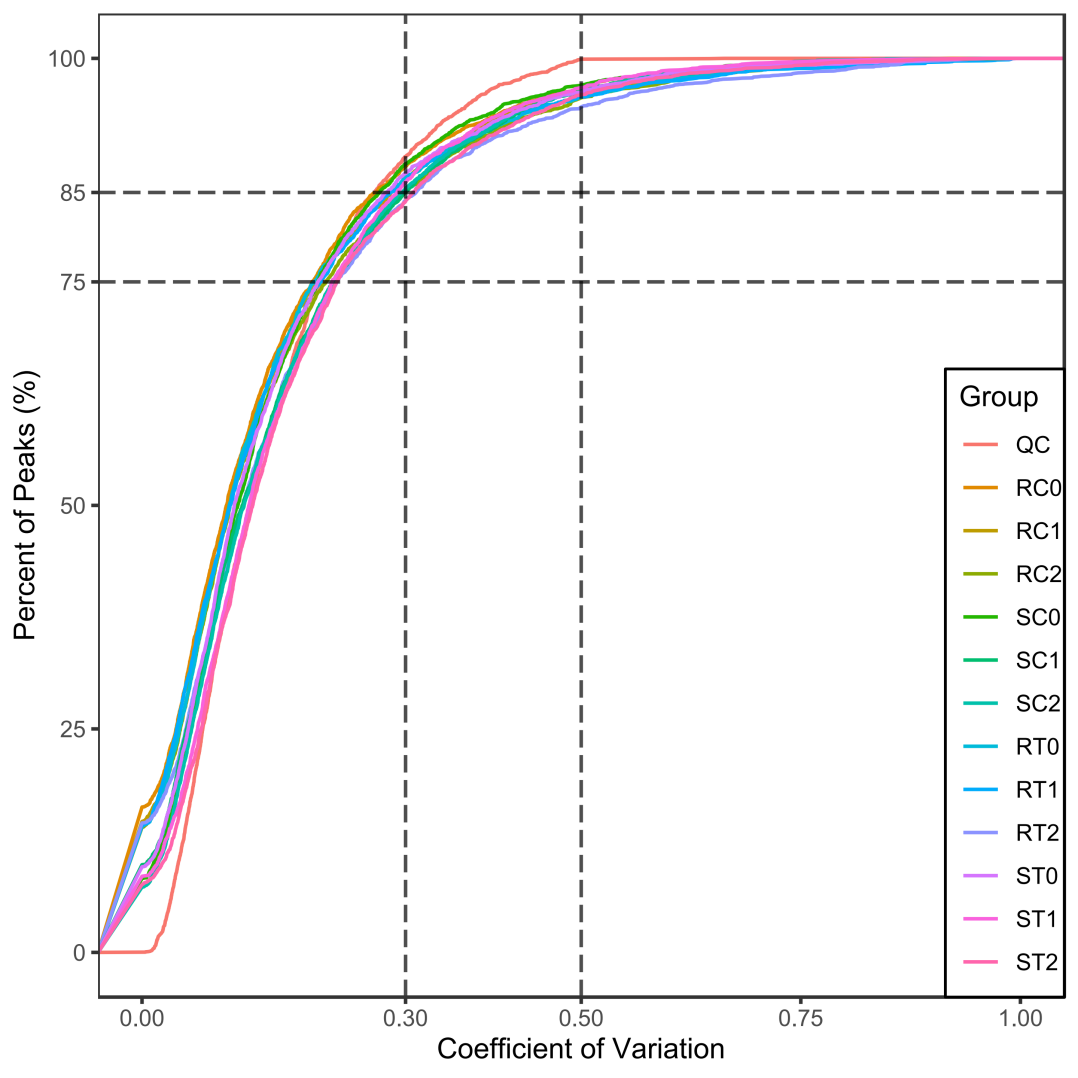
**

**Fige. S4** CV distribution of samples across all groups.

Note: The x-axis represents CV values, and the y-axis indicates the proportion of features with CV values below the corresponding threshold relative to the total number of features. Different colors denote different sample groups; QC indicates quality control samples. The two vertical reference lines correspond to CV values of 0.3 and 0.5, while the two horizontal reference lines correspond to 75% and 85% of the total features, respectively.

**Figure. S5** SelectPower of WGCNA

**
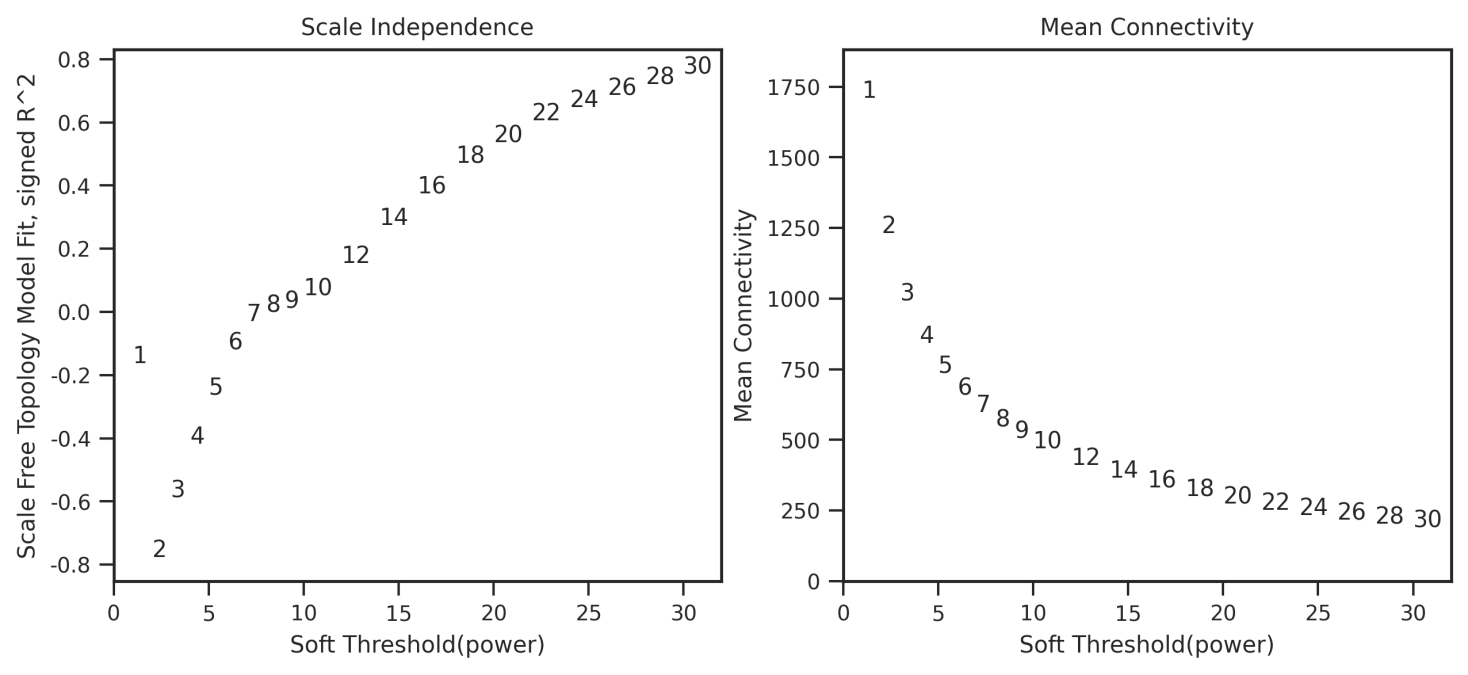
**
